# Highly Enantioselective Addition of Phenylethynylzinc to Aldehydes Catalyzed by Chiral Cyclopropane-Based Amino Alcohols

**DOI:** 10.3390/molecules181215422

**Published:** 2013-12-11

**Authors:** Bing Zheng, Zhiyuan Li, Feipeng Liu, Yanhua Wu, Junjian Shen, Qinghua Bian, Shicong Hou, Ming Wang

**Affiliations:** 1 Department of Applied Chemistry, China Agricultural University, 2 West Yuanmingyuan Road, Beijing 100193, China; E-Mails: bing17609@gmail.com (B.Z.); liufeipeng0302@163.com (F.L.); wyh64964478@sina.com (Y.W.); sjj06137@163.com (J.S.); wangmin@cau.edu.cn (M.W.); 2 Yanlin Agriculture Bureau, 20 Huadu Avenue, Yanlin 461200, Henan, China; E-Mail: caulizhiyuan@163.com

**Keywords:** alkynylation, aldehydes, cyclopropane-based, amino alcohols, asymmetric catalysis

## Abstract

The enantioselective addition of phenylethynylzinc to aldehydes catalyzed by a series of cyclopropane-based amino alcohol ligands **7** was investigated. The reactions afforded chiral propargylic alcohols in high yields (up to 96%) and with excellent enantioselectivities (up to 98% *ee*) under mild conditions. Furthermore, studies on the structural relationship show that the matching of the chiral center configuration is crucial to obtain the high enantioselectivity.

## 1. Introduction

**Figure 1:**
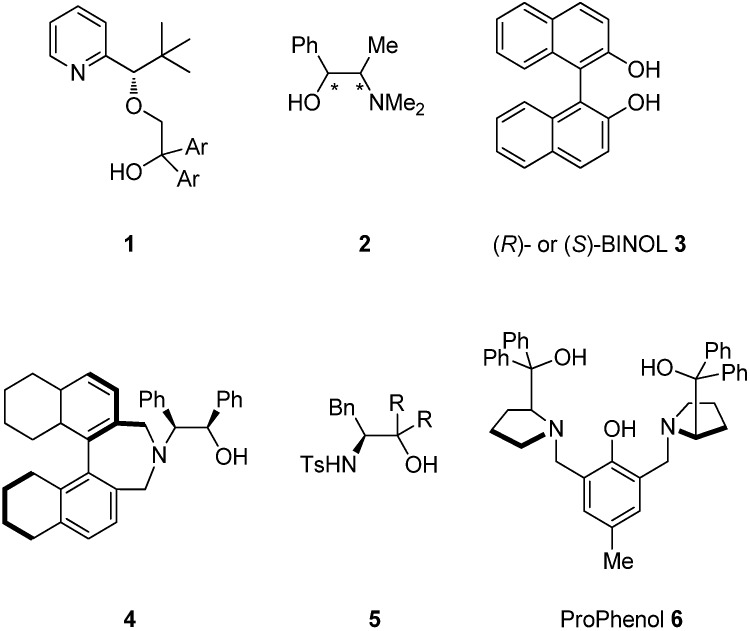
Chiral ligands for the enantioselective addition of alkynylzinc to aldehydes.

The catalytic enantioselective addition of alkynylzinc to aldehydes is one of the most useful carbon-carbon bond-forming reactions because the resulting propargylic alcohols are versatile, useful building blocks and important precursors for fine chemicals, pharmaceuticals, and natural products [1,2,3,4,5,6,7]. In 1994, Hoshino reported the first example of the addition of alkynylzinc reagents to cyclohexanecarbaldehyde and benzaldehyde using the ligand **1**, which afforded the corresponding products with high enantioselectivity [8]. Subsequently, various other catalytic system were reported, including (+)- or (–)-*N*-methylephedrine **2** by Carreira [9,10,11,12,13,14], (*R*)- or (*S*)-BINOL **3** and their derivatives by Pu [15,16,17,18,19,20,21,22,23,24], amino alcohols **4**, and *β*-sulfonamide alcohols by Chan [25,26,27,28], sulfonamide alcohols **5** and a bifunctional catalyst by Wang [29,30,31,32,33,34,35,36], and ProPhenol **6** by Trost [37,38,39,40] (Figure 1).

**Figure 2:**
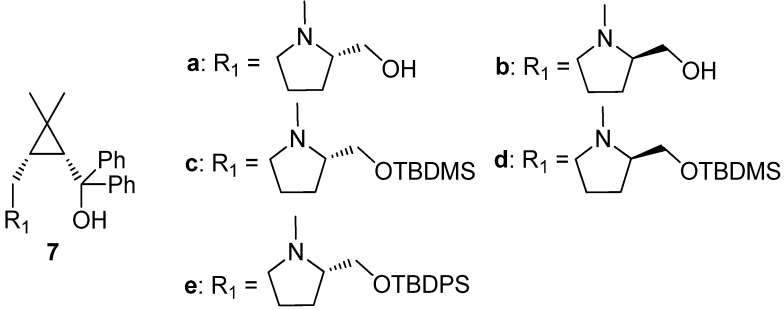
Cyclopropane amino alcohol **7a**–**e**.

Recently, we have developed a series of chiral cyclopropane-based ligands bearing amino alcohols, bisoxazolines, and amide alcohols (Figure 2). These ligands were proven to be very effective in some stereoselective reactions, including dialkylzinc addition to aldehydes and ketoesters, nitroaldol (Henry) reaction, Diels-Alder additions [41,42,43,44,45,46,47]. In this study, we focused on the structural relationship of our cyclopropane-based ligands amino alcohol **7** in the phenylethynylzinc addition to various aldehydes. It is noteworthy that the desired chiral propargylic alcohols were achieved with high to excellent yield (80%–96%). Importantly, high enantioselectivities (84%–98%) and broad substrate tolerance are also observed without any additives.

## 2. Results and Discussion

**Figure 3:**
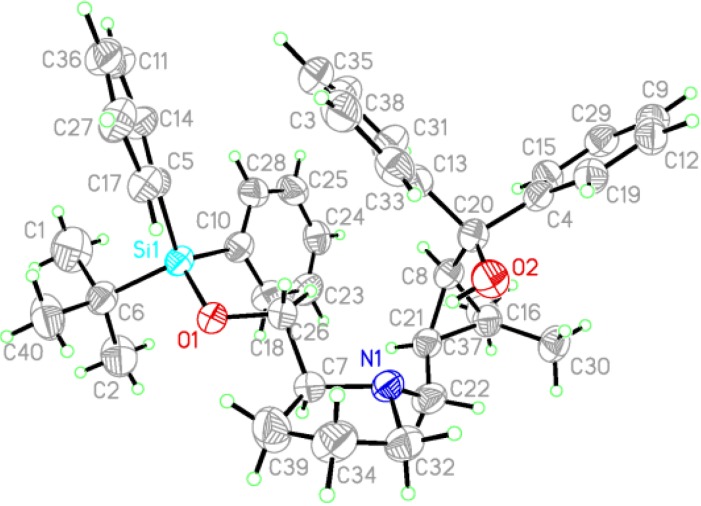
X-ray crystallographic structure of ligand **7e**.

The chiral ligands **7** were easily synthesised from commercially available (+)-*cis*-methyl chrysanthemate and (*R*) or (*S*)-prolinol according to a previously reported procedure [44]. The absolute configuration of ligands **7** was (*1R*, *3S*), as confirmed by X-ray crystallography analysis of ligand **7e** (Figure 3). This configuration is identical to that of the starting material, (+)-*cis*-methyl chrysanthemate.

An initial study on the structural relationship of the cyclopropane-based ligands **7** in the addition of phenylethynylzinc to benzaldehyde was performed (Table 1).

**Table 1:** Ligand survey for the addition of phenylethynylzinc to benzaldehyde *^a^*. 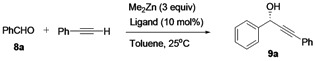

Entry	Ligand	Time (h)	Yield (%) *^b^*	*Ee* (%) *^c^*	Config. *^d^*
1	7a	20	89	10	*S*
2	7b	20	90	16	*S*
3	7c	20	94	80	*S*
4	7d	20	90	22	*S*
5	7e	20	91	79	*S*

*^a^* All reactions were run on a 1 mmol scale; *^b^* Isolated yields after chromatographic purification; *^c^* Enantiomeric excess determined by HPLC on a Chiracel OD-H column; *^d^* Absolute configuration assigned by comparing their specific rotations or the HPLC elution order with literature data.

The results indicated that varying the substitution on the pyrrolidine ring of the ligands significantly affected the enantioselectivity of the reaction. The *ee* value was significantly increased when the hydroxyl group in the prolinol of ligand **7a** was protected with a *tert*-butyldimethylsilyl chloride (TBDMSCl; ligand **7c**) or *tert*-butyldiphenylsilyl chloride (TBDPSCl; ligand **7e**) moiety (entry 1 *vs.* entries 3 and 5). Furthermore, the cyclopropane-based amino alcohol **7d**, which was synthesized from (*R*)-prolinol, afforded the corresponding (*S*)-propargyl alcohol with only 22% *ee*, whereas ligand **7c**, which was prepared from (*S*)-prolinol, exhibited a higher *ee* (80%). This result showed that the match of the cyclopropane configuration with the additional chiral center on the pyrrolidine was crucial to achieve high enantioselectivity. Therefore, the cyclopropane-based amino alcohol **7c** was the ligand of choice, providing the propargylic alcohol product with 80% *ee* (entry 3).

Attempts were made to optimize the reaction conditions by employing the addition of phenylethynylzinc to benzaldehyde. (Table 2). Early optimization showed that temperature significantly affected the *ee* value. A decrease in the reaction temperature from room temperature to 0 °C increased the *ee* values (entry 1 *vs.* entry 2). However, a further decrease in the temperature to −10 and −20 °C reduced both the enantioselectivity and yield (entries 3 and 4). It was interesting to note that the results were almost equally good when the amount of ligand was increased to 20 mol% (entry 6 *vs.* entry 2). Moreover, both the yield and enantioselectivity of the reaction decreased when the amount of ligand was reduced to 5 mol% (entry 7 *vs.* entry 2). Although previous studies [47] showed that the addition of polyethylene glycol dimethyl ether (DiMPEG) can significantly promote asymmetric induction, our additive to this reaction only slightly reduced the enantioselectivity (entry 5 *vs.* entry 2), because the DiMPEG would impact the generation of our unique catalyst system. Finally, the effects of the solvent on this reaction were investigated. Reaction in heptane gave lower enantioselectivity than in toluene (entry 8 *vs.* entry 2), this may be due to the poor solubility of Zn-amino alcohol complexes. Finally, the optimized reaction conditions were considered as following: **8a** (0.5 mmol) with phenylacetylene (1.5 mmol) and Me_2_Zn (1.5 mmol) in toluene at 0 °C for 48 h (Table 2, entry 2).

**Table 2:** Reaction optimization for the addition of phenylethynylzinc to benzaldehyde *^a^*. 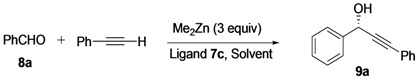

Entry	Ligand (mol %)	Solvent	Time (h)	Temp (°C)	Yield (%) *^b^*	*Ee* (%) *^c^*
1	10	Toluene	20	25	94	80
2	10	Toluene	48	0	91	93
3	10	Toluene	48	−10	83	86
4	10	Toluene	48	−20	40	79
5^d^	10	Toluene	48	0	95	90
6	20	Toluene	48	0	97	94
7	5	Toluene	48	0	75	85
8	10	Heptane	48	0	80	83

*^a^* All reactions were run on a 1 mmol scale; *^b^* Isolated yields after chromatographic purification; *^c^* Enantiomeric excess determined by HPLC on a Chiracel OD-H column; *^d^* With the addition of 10 mol % DiMPEG.

With the optimal condition in hand, we continued to explore the scope of this reaction. The addition of phenylethynylzinc to various aldehydes was investigated (Table 3). The results revealed that ligand **7c** was a highly enantioselective catalyst for the addition of alkynylzinc to aldehydes. *Ortho*-, *meta*-, and *para*-substituted benzaldehydes containing either electron-donating or electron-withdrawing substituents gave uniformly high ee (90%–98%, entries 1 to 13). In particular, the result (98% *ee*) obtained from 2-methylbenzaldehyde was remarkable (entry 5). High enantioselectivity was also observed for the addition to other aromatic aldehydes such as 1-naphthaldehyde and 2-naphthaldehyde (entries 14 and 15). A favorable result (entries 16 and 17) was also obtained when the aliphatic aldehydes **8p** and **8q** were used as substrates. 

**Table 3:** Substrate scope for the addition of phenylethynylzinc to aldehydes *^a^*. 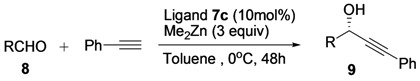

Entry	R	Product	Yield (%) *^b^*	*Ee* (%) *^c^*
1	Ph	**9a**	91	93
2	*p*-FC_6_H_4_	**9b**	90	94
3	*o*-BrC_6_H_4_	**9c**	96	94
4	*p*-NO_2_C_6_H_4_	**9d**	92	93
5	*o*-CH_3_C_6_H_4_	**9e**	91	98
6	*m*-CH_3_C_6_H_4_	**9f**	89	95
7	*p*-CH_3_C_6_H_4_	**9g**	90	95
8	*o*-CH_3_OC_6_H_4_	**9h**	81	96
9	*m*-CH_3_OC_6_H_4_	**9i**	80	94
10	*p*-CH_3_OC_6_H_4_	**9j**	85	97
11	*o*-ClC_6_H_4_	**9k**	95	90
12	*m*-ClC_6_H_4_	**9l**	93	93
13	*p*-ClC_6_H_4_	**9m**	92	93
14	1-Naphthyl	**9n**	91	98
15	2-Naphthyl	**9o**	80	92
16	Cyclohexyl	**9p**	92	84
17	Isopropyl	**9q**	91	88

*^a^* All reactions were run on a 0.5 mmol scale; *^b^* Isolated yields after chromatographic purification; *^c^* Enantiomeric excess determined by HPLC on a Chiracel OD-H column.

## 3. Experimental

### 3.1. General Methods and Materials

All reactions were performed under a nitrogen atmosphere. Solvents were dried according to standard procedures and were then distilled prior to use. All reagents were purchased commercially and used without further purification, unless stated otherwise. ^1^H- and ^13^C-NMR spectra were recorded using a Bruker DP-X300 MHz spectrometer (Bruker, Fallanden, Switzerland), and referenced internally to Me_4_Si. High-resolution mass spectra were obtained on an Agilent MS using the time-of-flight mass spectrometry technique (Agilent Technologies, Waldbroon, Germany). The optical rotations were determined on a Perkin-Elmer PE-341 polarimeter (Perkin-Elmer, Waltham, MA, USA). Crystallographic data were obtained using a Rigaku RAPID-S image plate X-Ray diffractometer (Rigaku Denki Co., Ltd, Tokyo, Japan). Enantiomeric excesses (*ee*) were determined on an Agilent 1100 HPLC system using a chiral Chiralcel OD-H column (Daicel Chiral Technologies (China) Co., Ltd., Shanghai, China) and isopropanol-hexanes as the eluent.

### 3.2. X-Ray Crystallographic Data of the Ligand **7e**

CCDC 808539 contains the supplementary crystallographic data for this paper. These data can be obtained free of charge via http://www.ccdc.cam.ac.uk/conts/retrieving.html (or from the CCDC, 12 Union Road, Cambridge CB2 1EZ, UK; Fax: +44 1223 336033; E-mail: deposit@ccdc.cam.ac.uk). This text may be included in the General subsection of the Experimental or as a suitably referenced endnote.

### 3.3. General Procedure for the Asymmetric Alkynylation of Aldehydes

Phenylacetylene (0.165 mL, 1.5 mmol, 3 equiv) was added to a solution of Me_2_Zn (1.25 mL, 1.2 M in toluene, 1.5 mmol, 3 equiv) in dry toluene (1.75 mL) at room temperature under a nitrogen atmosphere. The mixture was stirred for 30 min, then was transferred via syringe to another Schlenk tube containing neat ligand **7** (0.05 mmol, 0.1 equiv). After stirring for 30 min, an aldehyde (0.5 mmol) was added at 0 °C. The reaction mixture was stirred at 0 °C for 48 h and then quenched with saturated aqueous NH_4_Cl (5 mL). The organic phase was separated, and the aqueous phase was extracted with Et_2_O. The combined organic layers were dried over anhydrous Na_2_SO_4_. The solvents were removed under reduced pressure. Flash chromatography (silica gel, 10% ether in hexanes) afforded the pure propargylic alcohols. The enantiomeric excess was determined by HPLC on a Chiralcel OD-H column. The absolute configurations of the products were assigned by comparing their specific rotations or their HPLC elution order with literature data.

*(S)-1,3-Diphenylprop-2-yn-1-ol* (**9a**). 91% yield. [α]_D_^20^ = −3.8 (*c* = 1.52, CHCl_3_). ^1^H-NMR (CDCl_3_): δ 7.80–7.76 (m, 2H), 7.65–7.62 (m, 2H), 7.57–7.46 (m, 6H), 5.85 (d, *J* = 5.8 Hz, 1H), 2.47 (d, *J* = 5.9 Hz, 1H). ^13^C-NMR (CDCl_3_): δ 140.6, 131.7, 128.6, 128.5, 128.3, 128.2, 126.7, 122.3, 88.7, 86.6, 65.0. HRMS (TOF) calcd. for C_15_H_12_NaO [M+Na]^+^: 231.0786; found: 231.0776. 93% *ee* (80:20 *n*-hexane-2-propanol, 1.0 mL/min, 254 nm). Retention time: t_minor_ = 8.13 min, t_major_ = 10.27 min.

*(S)-1-(4-Fluorophenyl)-3-phenylprop-2-yn-1-ol* (**9b**). 90% yield. [α]_D_^20^ = −4.0 (*c* = 1.50, CHCl_3_). ^1^H-NMR (CDCl_3_): δ 7.62–7.57 (m, 2H), 7.49–7.45 (m, 2H), 7.35–7.32 (m, 3H), 7.11–7.05 (m, 2H), 5.67 (d, *J* = 6.0 Hz, 1H), 2.34 (d, *J* = 6.1 Hz, 1H). ^13^C-NMR (CDCl_3_): δ 164.2, 136.4, 131.6, 128.6, 128.4, 128.3, 122.1, 115.4 88.5, 86.7, 64.2. HRMS (TOF) calcd. for C_15_H_11_FNaO [M+Na]^+^: 249.0692; found: 249.0685. 94% *ee* (80:20 *n*-hexane-2-propanol, 1.0 mL/min, 254 nm). Retention time: t_minor_ = 6.45 min, t_major_ = 12.47 min.

*(S)-1-(2-Bromophenyl)-3-phenylprop-2-yn-1-ol* (**9c**). 96% yield. [α]_D_^20^ = +71.9 (*c* = 1.01, CHCl_3_). ^1^H-NMR (CDCl_3_): δ 7.85 (dd, *J* = 1.7, 7.7 Hz, 1H), 7.59 (dd, *J* =1.2, 8.0 Hz, 1H), 7.49–7.21 (m, 7H), 6.02 (d, *J* = 5.5 Hz, 1H), 2.56 (d, *J* = 5.6 Hz, 1H). ^13^C-NMR (CDCl_3_): δ 139.4, 132.9, 131.7, 129.8, 128.6, 128.2, 127.8, 122.7, 122.2, 87.6, 86.6, 64.5. HRMS (TOF) calcd. for C_15_H_11_BrNaO [M+Na]^+^: 308.9891; found: 308.9894. 94% *ee* (80:20 *n*-hexane-2-propanol, 1.0 mL/min, 254 nm). Retention time: t_major_ = 6.44 min, t_minor_ = 6.92 min.

*(S)-1-(4-Nitrophenyl)-3-phenylprop-2-yn-1-ol* (**9d**). 92% yield. [α]_D_^20^ = −12.8 (*c* = 1.00, CHCl_3_). ^1^H-NMR (CDCl_3_): δ 8.27 (dd, *J* = 2.0, 6.8 Hz, 1H), 7.82–7.78 (m, 2H), 7.49–7.45 (m, 2H), 7.37–7.34 (m, 3H), 5.80 (d, *J* = 5.6 Hz, 1H), 2.45 (d, *J* = 5.7 Hz, 1H). ^13^C-NMR (CDCl_3_): δ 147.9, 147.4, 131.8, 129.1, 128.4, 127.4, 123.8, 121.7, 87.7, 87.4, 64.1. HRMS (TOF) calcd for C_15_H_12_NO_3_ [M+H]^+^: 254.0817; found: 254.0813. 93% *ee* (80:20 *n*-hexane-2-propanol, 1.0 mL/min, 254 nm). Retention time: t_minor_ = 9.50 min, t_major_ = 27.68 min. 

*(S)-1-(2-Methylphenyl)-3-phenylprop-2-yn-1-ol* (**9e**). 91% yield. [α]_D_^20^ = +13.6 (*c* = 0.73, CHCl_3_). ^1^H-NMR (CDCl_3_): δ 7.74–7.71 (m, 1H), 7.48–7.45 (m, 2H), 7.33–7.30 (m, 3H), 7.27–7.20 (m, 3H), 5.84 (s, 1H), 2.50 (s, 3H), 2.18 (br, 1H). ^13^C-NMR (CDCl_3_): δ 138.3, 136.0, 131.7, 130.8, 128.5, 128.4, 128.2, 126.5, 126.2, 122.5, 88.5, 86.4, 62.9, 19.0. HRMS (TOF) calcd. for C_16_H_14_NaO [M+Na]^+^: 245.0942; found: 245.0938. 98% *ee* (80:20 *n*-hexane-2-propanol, 1.0 mL/min, 254 nm). Retention time: t_minor_ = 6.29 min, t_major_ = 9.72 min.

*(S)-1-(3-Methylphenyl)-3-phenylprop-2-yn-1-ol* (**9f**). 89% yield. [α]_D_^20^ = −6.8 (*c* = 1.11, CHCl_3_). ^1^H-NMR (CDCl_3_): δ 7.49–7.40 (m, 4H), 7.33–7.30 (m, 4H), 7.17 (d, *J* = 7.6 Hz, 1H), 5.66 (d, *J* = 5.8 Hz, 1H), 2.39 (s, 3H), 2.23 (d, *J* = 6.1 Hz, 1H). ^13^C-NMR (CDCl_3_): δ 140.5, 138.4, 131.7, 129.2, 128.55, 128.53, 128.3, 127.4, 123.7, 122.4, 88.8, 86.5, 65.1, 21.4. HRMS (TOF) calcd. for C_16_H_14_NaO [M+Na]^+^: 245.0942; found: 245.0938. 95% *ee* (80:20 *n*-hexane-2-propanol, 1.0 mL/min, 254 nm). Retention time: t_minor_ = 7.12 min, t_major_ = 11.27 min.

*(S)-1-(4-Methylphenyl)-3-phenylprop-2-yn-1-ol* (**9g**). 90% yield. [α]_D_^20^ = −5.9 (*c* = 0.76, CHCl_3_). ^1^H-NMR (CDCl_3_): δ 7.52–7.45 (m, 4H), 7.33–7.20 (m, 5H), 5.66 (s, 1H), 2.37 (s, 3H), 2.26 (br, 1H). ^13^C-NMR (CDCl_3_): δ 138.2, 137.8, 131.7, 129.3, 128.5, 128.3, 126.7, 122.5, 88.9, 86.4, 64.9, 21.1. HRMS (TOF) calcd. for C_16_H_14_NaO [M+Na]^+^: 245.0942; found: 245.0948. 95% *ee* (80:20 *n*-hexane-2-propanol, 1.0 mL/min, 254 nm). Retention time: t_minor_ = 6.53 min, t_major_ = 9.69 min. 

*(S)-1-(2-Methoxyphenyl)-3-phenylprop-2-yn-1-ol* (**9h**). 81% yield. [α]_D_^20^ = +12.3 (*c* = 2.03, CHCl_3_). ^1^H-NMR (CDCl_3_): δ 7.65 (dd, *J* = 1.8, 7.6 Hz, 1H), 7.49–7.46 (m, 2H), 7.33–7.29 (m, 4H), 7.00–6.92 (m, 2H), 5.93 (s, 1H), 3.91(s, 3H), 3.07 (br, 1H). ^13^C-NMR (CDCl_3_): δ 156.8, 131.7, 129.6, 128.9, 128.3, 128.1, 127.9, 122.7, 120.8, 110.9, 88.5, 85.9, 61.5, 55.5. HRMS (TOF) calcd. for C_16_H_14_NaO_2_ [M+Na]^+^: 261.0891; found: 261.0895. 96% *ee* (80:20 *n*-hexane-2-propanol, 1.0 mL/min, 254 nm). Retention time: t_minor_ = 9.19 min, t_major_ = 10.16 min.

*(S)-1-(3-Methoxyphenyl)-3-phenylprop-2-yn-1-ol* (**9i**). 80% yield. [α]_D_^20^ = −12.9 (*c* = 1.04, CHCl_3_). ^1^H-NMR (CDCl_3_): δ 7.49–7.45 (m, 2H), 7.33–7.29 (m, 4H), 7.21–7.18 (m, 2H), 6.91–6.88 (m, 1H), 5.66 (s, 1H), 3.83 (s, 3H), 2.27 (br, 1H). ^13^C-NMR (CDCl_3_): δ 159.7, 142.2, 131.7, 129.6, 128.5, 128.2, 122.3, 118.9, 114.0, 112.1, 88.7, 86.4, 64.8, 55.2. HRMS (TOF) calcd. for C_16_H_14_NaO_2_ [M+Na]^+^: 261.0891; found: 261.0885. 94% *ee* (80:20*n*-hexane-2-propanol, 1.0 mL/min, 254 nm). Retention time: t_minor_ = 11.43 min, t_major_ = 14.51 min. 

*(S)-1-(4-Methoxyphenyl)-3-phenylprop-2-yn-1-ol* (**9j**). 85% yield. [α]_D_^20^ = −5.3 (*c* = 1.16, CHCl_3_). ^1^H-NMR (CDCl_3_): δ 7.55 (dd, *J* = 2.1, 6.7 Hz, 2H), 7.49–7.46 (m, 2H), 7.33–7.31 (m, 3H), 6.93 (dd, *J* = 2.0, 6.7 Hz, 2H), 5.65 (d, *J* = 6.0 Hz, 1H), 3.83 (s, 3H), 2.18 (d, *J* = 6.2 Hz, 1H). ^13^C-NMR (CDCl_3_): δ 159.7, 133.0, 131.7, 128.5, 128.3, 128.1, 122.5, 114.0, 89.0, 86.5, 64.7, 55.3. HRMS (TOF) calcd. for C_16_H_14_NaO_2_ [M+Na]^+^: 261.0891; found: 261.0887. 97% *ee* (80:20 *n*-hexane-2-propanol, 1.0 mL/min, 254 nm). Retention time: t_minor_ = 10.05 min, t_major_ = 14.41 min. 

*(S)-1-(2-Chlorophenyl)-3-phenylprop-2-yn-1-ol* (**9k**). 95% yield. [α]_D_^20^ = +12.1 (*c* = 1.20, CHCl_3_). ^1^H-NMR (CDCl_3_): δ 7.85–7.82 (m, 1H), 7.49–7.26 (m, 8H), 6.05 (d, *J* = 4.5Hz, 1H), 2.53 (d, *J* = 5.1Hz, 1H). ^13^C-NMR (CDCl_3_): δ 137.9, 132.8, 131.7, 129.75, 129.67, 128.6, 128.4, 128.3, 127.2, 122.3, 87.6, 86.6, 62.4. HRMS (TOF) calcd. for C_15_H_11_ClNaO [M+Na]^+^: 265.0396; found: 265.0396. 90% *ee* (97:3 *n*-hexane-2-propanol, 1.0 mL/min, 254 nm). Retention time: t_minor_ = 29.93 min, t_major_ = 34.49 min.

*(S)-1-(3-Chlorophenyl)-3-phenylprop-2-yn-1-ol* (**9l**). 93% yield. [α]_D_^20^ = −8.6 (*c* = 1.54, CHCl_3_). ^1^H-NMR (CDCl_3_): δ 7.56 (t, *J* = 0.5 Hz, 1H), 7.44–7.41 (m, 3H), 7.29–7.24 (m, 5H), 5.60 (s, 1H), 3.10 (s, 1H). ^13^C-NMR (CDCl_3_): δ 142.4, 134.3, 131.7, 129.8, 128.7, 128.4, 128.2, 126.8, 124.7, 122.0, 88.0, 86.9, 64.2. HRMS (TOF) calcd. for C_15_H_11_ClNaO [M+Na]^+^: 265.0396; found: 265.0393. 93% *ee* (80:20 *n*-hexane-2-propanol, 1.0 mL/min, 254 nm). Retention time: t_minor_ = 6.05 min, t_major_ = 13.35 min.

*(S)-1-(4-Chlorophenyl)-3-phenylprop-2-yn-1-ol* (**9m**). 92% yield. [α]_D_^20^ = −9.0 (*c* = 1.01, CHCl_3_). ^1^H-NMR (CDCl_3_): δ 7.57–7.32 (m, 9H), 5.67 (s, 1H), 2.30 (br, 1H). ^13^C-NMR (CDCl_3_): δ 139.0, 134.1, 131.7, 128.7, 128.6, 128.3, 128.0, 122.0, 88.2, 86.8, 64.2. HRMS (TOF) calcd. for C_15_H_11_ClNaO [M+Na]^+^: 265.0396; found: 265.0390. 93% *ee* (80:20 *n*-hexane-2-propanol, 1.0 mL/min, 254 nm). Retention time: t_minor_ = 5.96 min, t_major_ = 12.43 min.

*(S)-1-(1-Naphthyl)-3-phenylprop-2-yl-1-ol* (**9n**). 91% yield. [α]_D_^20^ = +35.3 (*c* = 1.00, CHCl_3_). ^1^H-NMR (CDCl_3_): δ 8.36 (d, *J* = 8.4, 2H), 7.92–7.84 (m, 3H), 7.58–7.46 (m, 5H), 7.32–7.29 (m, 3H), 6.34 (s, 1H), 2.45 (br, 1H). ^13^C-NMR (CDCl_3_): δ 135.5, 133.8, 131.6, 130.4, 129.2, 128.6, 128.4, 128.1, 126.3, 125.7, 125.1, 124.5, 123.9, 122.3, 88.6, 87.1, 63.1. HRMS (TOF) calcd. for C_19_H_14_NaO [M+Na]^+^: 281.0942; found: 281.0938. 98% *ee* (80:20 *n*-hexane-2-propanol, 1.0 mL/min, 254 nm). Retention time: t_minor_ = 9.46 min, t_major_ = 15.77 min. 

*(S)-1-(2-Naphthyl)-3-phenylprop-2-yl-1-ol* (**9o**). 80% yield. [α]_D_^20^ = +8.6 (*c* = 0.70, CHCl_3_). ^1^H-NMR (CDCl_3_): δ 8.06 (s, 1H), 7.91–7.87 (m, 3H), 7.73 (dd, *J* = 1.7, 8.4 Hz, 1H), 7.52–7.49 (m, 4H), 7.35–7.33 (m, 3H), 5.87 (d, *J* = 6.2 Hz, 1H), 2.35 (d, *J* = 6.2 Hz, 1H). ^13^C-NMR (CDCl_3_): δ 138.0, 133.3, 133.2, 131.8, 128.7, 128.6, 128.3, 128.2, 127.7, 126.3, 125.5, 124.6, 122.4, 88.7, 87.0, 65.3. HRMS (TOF) calcd. for C_19_H_14_NaO [M+Na]^+^: 281.0942; found: 281.0938. 92% *ee* (80:20 *n*-hexane-2-propanol, 1.0 mL/min, 254 nm). Retention time: t_minor_ = 9.34 min, t_major_ = 21.95 min.

*(S)-1-Cyclohexyl-3-phenylprop-2-yn-1-ol* (**9p**). 92% yield. [α]_D_^20^ = +7.9 (*c* = 0.71, CHCl_3_). ^1^H-NMR (CDCl_3_): δ 7.45–7.41 (m, 2H), 7.33–7.28 (m, 3H), 4.38 (t, *J* = 5.9, 1H), 1.95–1.90 (m, 2H), 1.86–1.78 (m, 3H), 1.72–1.64 (m, 2H), 1.32–1.11 (m, 5H). ^13^C-NMR (CDCl_3_): δ 131.7, 128.27, 128.24, 122.8, 89.3, 85.7, 67.7, 44.3, 28.6, 28.2, 26.4, 25.92, 25.90. HRMS (TOF) calcd. for C_15_H_18_NaO [M+Na]^+^: 237.1255; found: 237.1250. 84% *ee* (80:20 *n*-hexane-2-propanol, 1.0 mL/min, 254 nm). Retention time: t_minor_ = 4.35 min, t_major_ = 6.32 min.

*(S)-4-Methyl-1-phenylpent-1-yl-3-ol* (**9q**). 91% yield. [α]_D_^20^ = +1.6 (*c* = 1.35, CHCl_3_). ^1^H-NMR (CDCl_3_): δ 7.45–7.42 (m, 2H), 7.33–7.28 (m, 3H), 4.40 (d, *J* = 5.6, 1H), 2.01–1.95 (m, 1H), 1.87 (br, 1H), 1.07 (t, *J* = 6.7, 6H). ^13^C-NMR (CDCl_3_): δ 131.7, 128.28, 128.23, 122.7, 88.9, 85.6, 68.4, 34.7, 18.1, 17.5. HRMS (TOF) calcd. for C_12_H_14_NaO [M+Na]^+^: 197.0942; found: 197.0941. 88% *ee* (80:20 *n*-hexane-2-propanol, 1.0 mL/min, 254 nm). Retention time: t_minor_ = 4.22 min, t_major_ = 5.71 min.

## 4. Conclusions

The cyclopropane-based amino alcohol **7c** successfully promotes the enantioselective alkynylation of aldehydes and affords chiral propargylic alcohols in high yields and high enantiomeric excess (up to 98% *ee*) without requiring any additives. In addition, studies on the structural relationship show that the matching of the cyclopropane configuration with the additional chiral center on pyrrolidine is crucial to obtain high enantioselectivity.

## Supplementary Materials

Supplementary materials can be accessed at: http://www.mdpi.com/1420-3049/18/12/15422/s1.
