# Supplementary material for: Highly Enantioselective Addition of Phenylethynylzinc to Aldehydes Catalyzed by Chiral Cyclopropane-Based Amino Alcohols
Source: Molecules. 2013 Dec 11;18(12):15422–33. doi: 10.3390/molecules181215422 (PMC6270052; doi:10.3390/molecules181215422)
Supplement: Supplementary file 1 [file molecules-18-15422-s001.pdf]

Supplementary Materials for
Highly Enantioselective Addition of Phenylethynylzinc to Aldehydes Catalyzed by Chiral Cyclopropane-Based Amino Alcohols

Table Content

1. X-ray crystallographic data of the ligand **7e** S1
2. NMR Spectra for the Propargylic Alcohols Products S2–S18
3. HPLC Spectra of the Propargylic Alcohols Products S19–S35

Table S1. Crystal Data and Structure Refinement for **7e**.

**Empirical Formula**	**$C_{40}H_{49}NO_2Si$**	
**Formula weight**	603.89	
**Temperature**	173(2) K	
**Wavelength**	0.71073 A	
**Crystal system, space group**	Orthorhombic, P2(1)2(1)2(1)	
**Unit cell dimensions**	*a* = 9.3798(19) Å	alpha = 90 deg.
	b = 13.079(3) Å	beta = 90 deg.
	c = 28.827(6) Å	gamma = 90 deg.
**Volume**	3536.5(12) $Å^3$	
***Z*, Calculated density**	4, 1.134 Mg/m^3	
**Absorption coefficient**	0.100 mm^{-1}	
***F* (000)**	1304	
**Crystal size**	0.80 × 0.70 × 0.60 mm	
**Theta range for data collection**	2.10 to 26.35 deg.	
**Limiting indices**	−11 <= h<=11, −14 <= k<=16, −34 <= l <= 36	
**Reflections collected/unique**	24411/7202 [*R* (int) = 0.0523]	
**Completeness to theta = 26.35**	99.9%	
**Absorption correction**	Semi-empirical from equivalents	
**Max. and min. transmission**	0.9424 and 0.9242	
**Refinement method**	Full-matrix least-squares on F^2	
**Data/restraints/parameters**	7202/0/397	
**Goodness-of-fit on F^2**	1.175	
**Final *R* indices [*I* > 2sigma (*I*)]**	R_1 = 0.0597, wR_2 = 0.1444	
***R* indices (all data)**	R_1 = 0.0630, wR_2 = 0.1465	
**Absolute structure parameter**	−0.13(15)	
**Largest diff. peak and hole**	0.219 and −0.345 $e.A^{-3}$	

Figure S1. NMR Spectra for the Propargylic Alcohols Products.

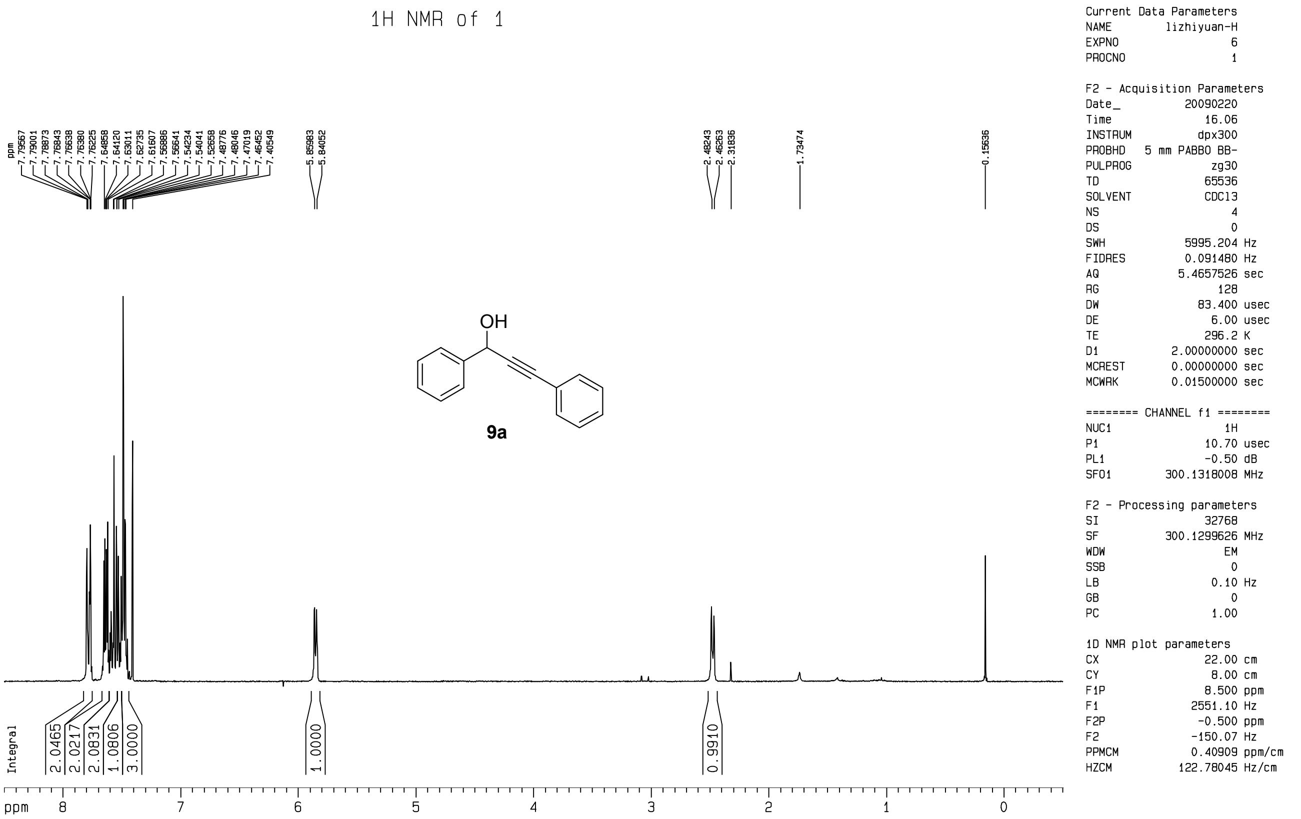

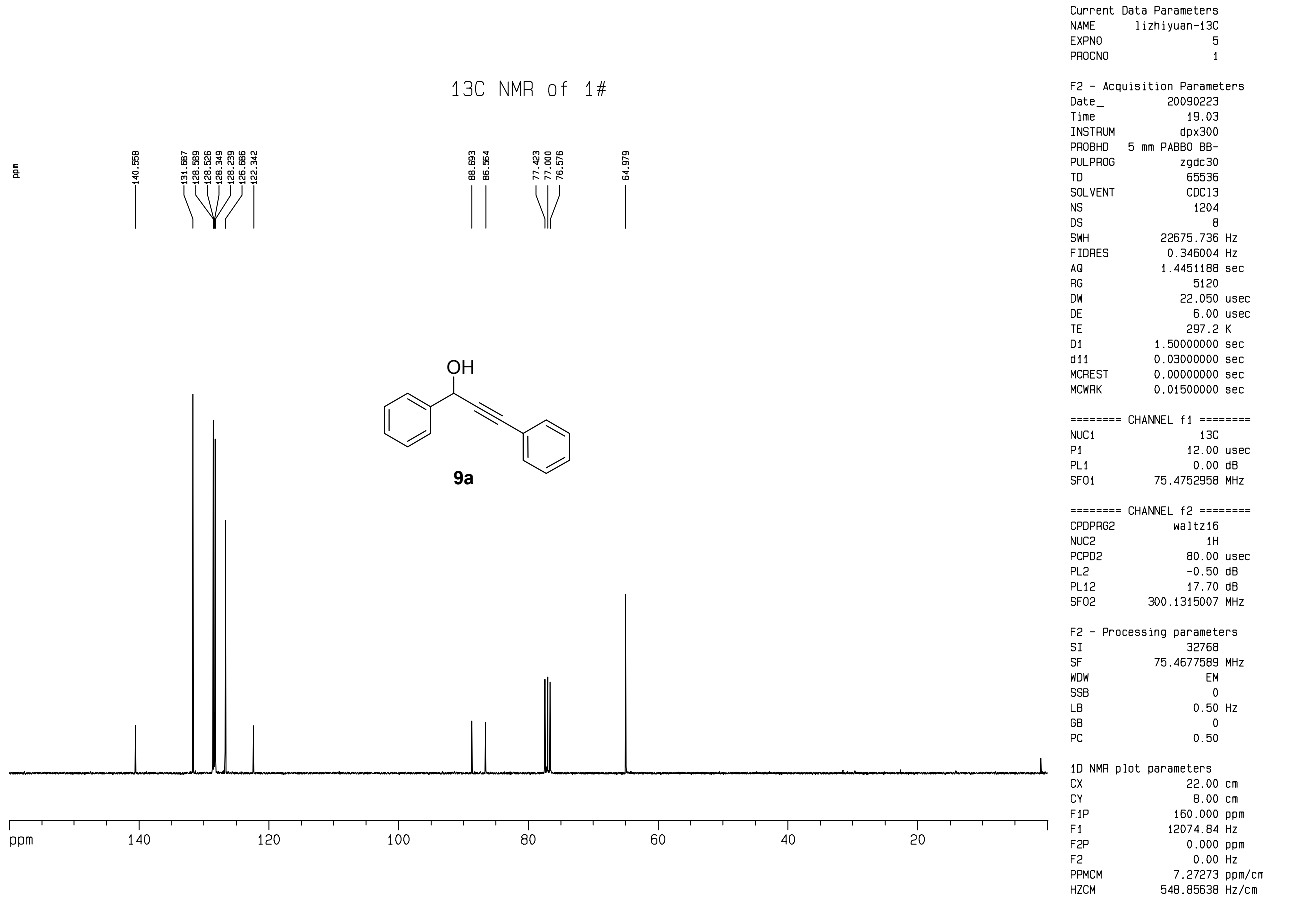

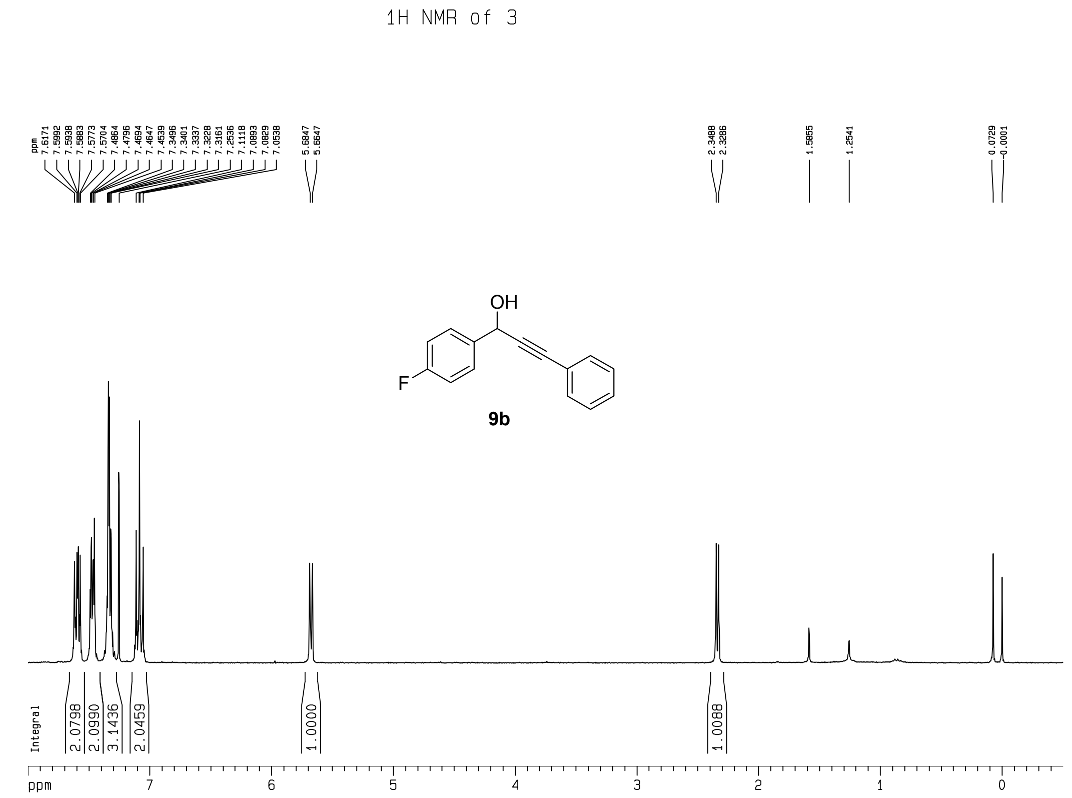

Current Data Parameters
NAME lizhiyuan-H
EXPNO 12
PROCNO 1

F2 - Acquisition Parameters
Date_ 20090226
Time 10.55
INSTRUM dpx300
PROBHD 5 mm PABBO BB-
PULPROG zg30
TD 65536
SOLVENT CDCl3
NS 6
DS 0
SWH 5995.204 Hz
FIDRES 0.091480 Hz
AQ 5.4657526 sec
RG 258
DW 83.400 usec
DE 6.00 usec
TE 295.2 K
D1 2.00000000 sec
MCREST 0.00000000 sec
MCWRK 0.01500000 sec

======== CHANNEL f1 ========
NUC1 1H
P1 10.70 usec
PL1 -0.50 dB
SFO1 300.1318008 MHz

F2 - Processing parameters
SI 32768
SF 300.1300081 MHz
WDW EM
SSB 0
LB 0.10 Hz
GB 0
PC 1.00

1D NMR plot parameters
CX 22.00 cm
CY 6.00 cm
F1P 8.000 ppm
F1 2401.04 Hz
F2P -0.500 ppm
F2 -150.06 Hz
PPMCM 0.38636 ppm/cm
HZCM 115.95932 Hz/cm

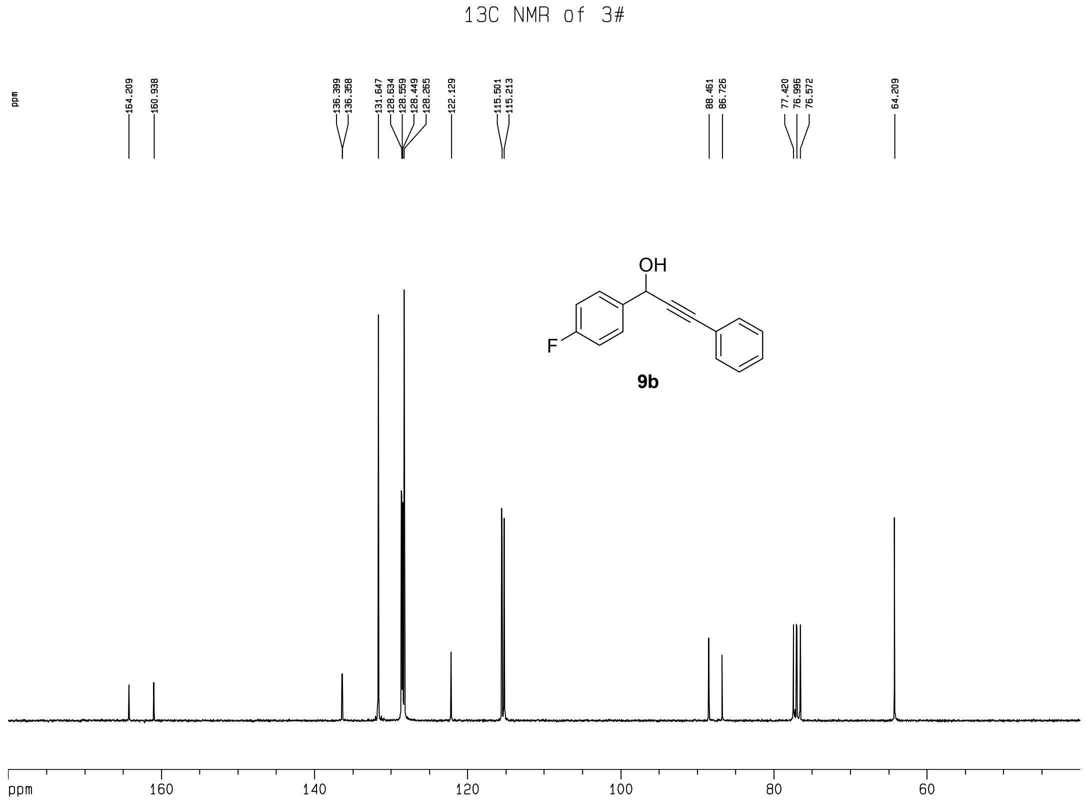

Current Data Parameters
NAME lizhiyuan-13C
EXPNO 8
PROCNO 1

F2 - Acquisition Parameters
Date_ 20090226
Time 17.44
INSTRUM dpx300
PROBHD 5 mm PABBO BB-
PULPROG zgdc30
TD 65536
SOLVENT CDCl3
NS 1342
DS 8
SWH 22675.736 Hz
FIDRES 0.346004 Hz
AQ 1.4451188 sec
RG 5120
DW 22.050 usec
DE 6.00 usec
TE 297.2 K
D1 1.50000000 sec
d11 0.03000000 sec
MCREST 0.00000000 sec
MCWRK 0.01500000 sec

======== CHANNEL f1 ========
NUC1 13C
P1 12.00 usec
PL1 0.00 dB
SFO1 75.4752958 MHz

======== CHANNEL f2 ========
CPDPRG2 waltz16
NUC2 1H
PCPD2 80.00 usec
PL2 -0.50 dB
PL12 17.70 dB
SFO2 300.1315007 MHz

F2 - Processing parameters
SI 32768
SF 75.4677603 MHz
WDW EM
SSB 0
LB 1.00 Hz
GB 0
PC 0.50

1D NMR plot parameters
CX 22.00 cm
CY 9.00 cm
F1P 180.000 ppm
F1 13584.20 Hz
F2P 40.000 ppm
F2 3018.71 Hz
PPMCM 6.36364 ppm/cm
HZCM 480.24936 Hz/cm

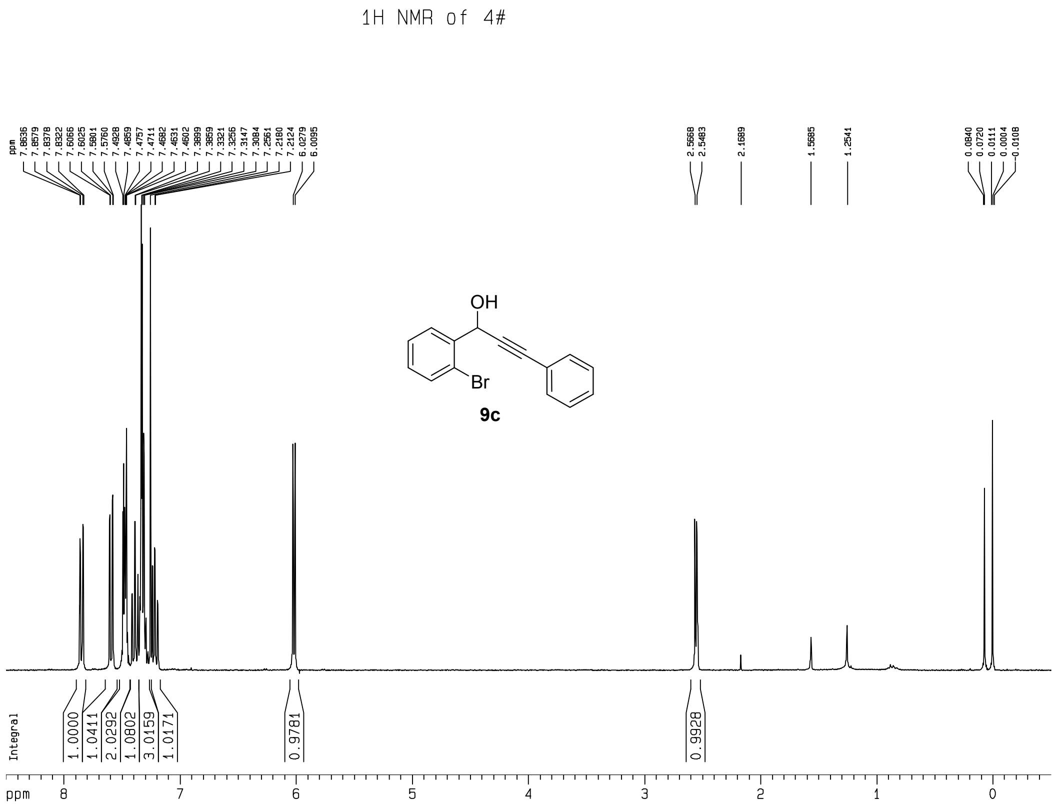

Current Data Parameters
NAME lizhiyuan-H
EXPNO 17
PROCNO 1

F2 - Acquisition Parameters
Date_ 20090306
Time 12.04
INSTRUM dpx300
PROBHD 5 mm PABBO BB-
PULPROG zg30
TD 65536
SOLVENT CDCl3
NS 20
DS 0
SWH 5995.204 Hz
FIDRES 0.091480 Hz
AQ 5.4657526 sec
RG 258
DW 83.400 usec
DE 6.00 usec
TE 296.2 K
D1 2.00000000 sec
MCREST 0.00000000 sec
MCWRK 0.01500000 sec

======== CHANNEL f1 ========
NUC1 1H
P1 10.70 usec
PL1 -0.50 dB
SFO1 300.1318008 MHz

F2 - Processing parameters
SI 32768
SF 300.1300074 MHz
WDW EM
SSB 0
LB 0.10 Hz
GB 0
PC 1.00

1D NMR plot parameters
CX 22.00 cm
CY 10.00 cm
F1P 8.500 ppm
F1 2551.10 Hz
F2P -0.500 ppm
F2 -150.07 Hz
PPMCM 0.40909 ppm/cm
HZCM 122.78046 Hz/cm

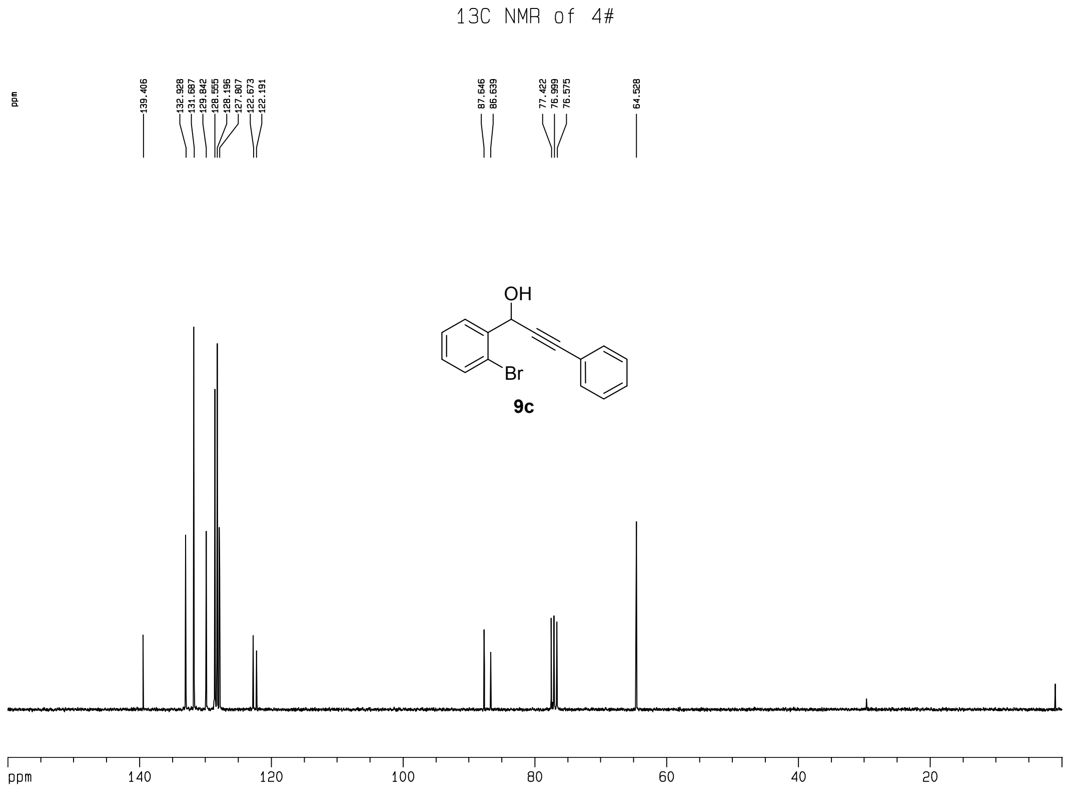

Current Data Parameters
NAME lizhiyuan-13C
EXPNO 18
PROCNO 1

F2 - Acquisition Parameters
Date_ 20090307
Time 15.50
INSTRUM dpx300
PROBHD 5 mm PABBO BB-
PULPROG zgdc30
TD 65536
SOLVENT CDCl3
NS 326
DS 8
SWH 22675.736 Hz
FIDRES 0.346004 Hz
AQ 1.4451188 sec
RG 7168
DW 22.050 usec
DE 6.00 usec
TE 297.2 K
D1 1.50000000 sec
d11 0.03000000 sec
MCREST 0.00000000 sec
MCWRK 0.01500000 sec

======== CHANNEL f1 ========
NUC1 13C
P1 12.00 usec
PL1 0.00 dB
SFO1 75.4752958 MHz

======== CHANNEL f2 ========
CPDPRG2 waltz16
NUC2 1H
PCPD2 80.00 usec
PL2 -0.50 dB
PL12 17.70 dB
SFO2 300.1315007 MHz

F2 - Processing parameters
SI 32768
SF 75.4677610 MHz
WDW EM
SSB 0
LB 1.00 Hz
GB 0
PC 0.50

1D NMR plot parameters
CX 22.00 cm
CY 8.00 cm
F1P 160.000 ppm
F1 12074.84 Hz
F2P 0.000 ppm
F2 0.00 Hz
PPMCM 7.27273 ppm/cm
HZCM 548.85638 Hz/cm

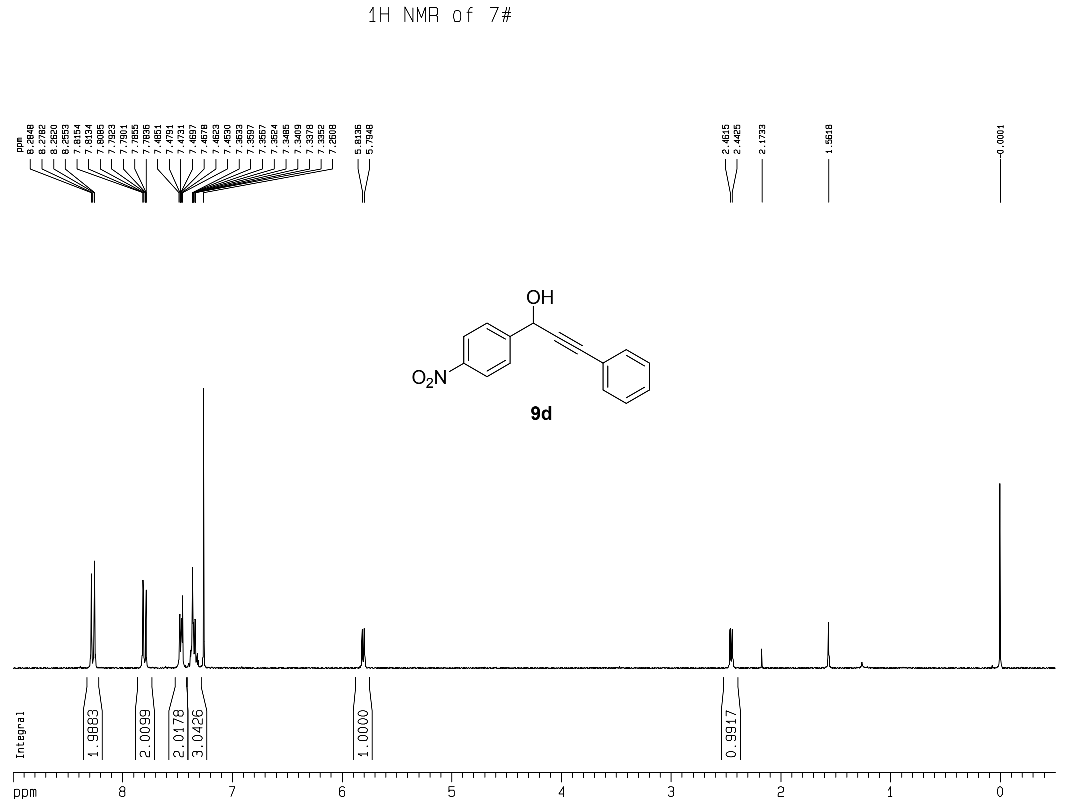

Current Data Parameters
NAME lizhiyuan-H
EXPNO 35
PROCNO 1

F2 - Acquisition Parameters
Date_ 20090327
Time 10.25
INSTRUM dpx300
PROBHD 5 mm PABBO BB-
PULPROG zg30
TD 65536
SOLVENT CDCl3
NS 4
DS 0
SWH 5995.204 Hz
FIDRES 0.091480 Hz
AQ 5.4657526 sec
RG 258
DW 83.400 usec
DE 6.00 usec
TE 296.2 K
D1 2.00000000 sec
MCREST 0.00000000 sec
MCWRK 0.01500000 sec

======== CHANNEL f1 ========
NUC1 1H
P1 10.70 usec
PL1 -0.50 dB
SFO1 300.1318008 MHz

F2 - Processing parameters
SI 32768
SF 300.1300060 MHz
WDW EM
SSB 0
LB 0.10 Hz
GB 0
PC 1.00

1D NMR plot parameters
CX 22.00 cm
CY 6.00 cm
F1P 9.000 ppm
F1 2701.17 Hz
F2P -0.500 ppm
F2 -150.07 Hz
PPMCM 0.43182 ppm/cm
HZCM 129.60159 Hz/cm

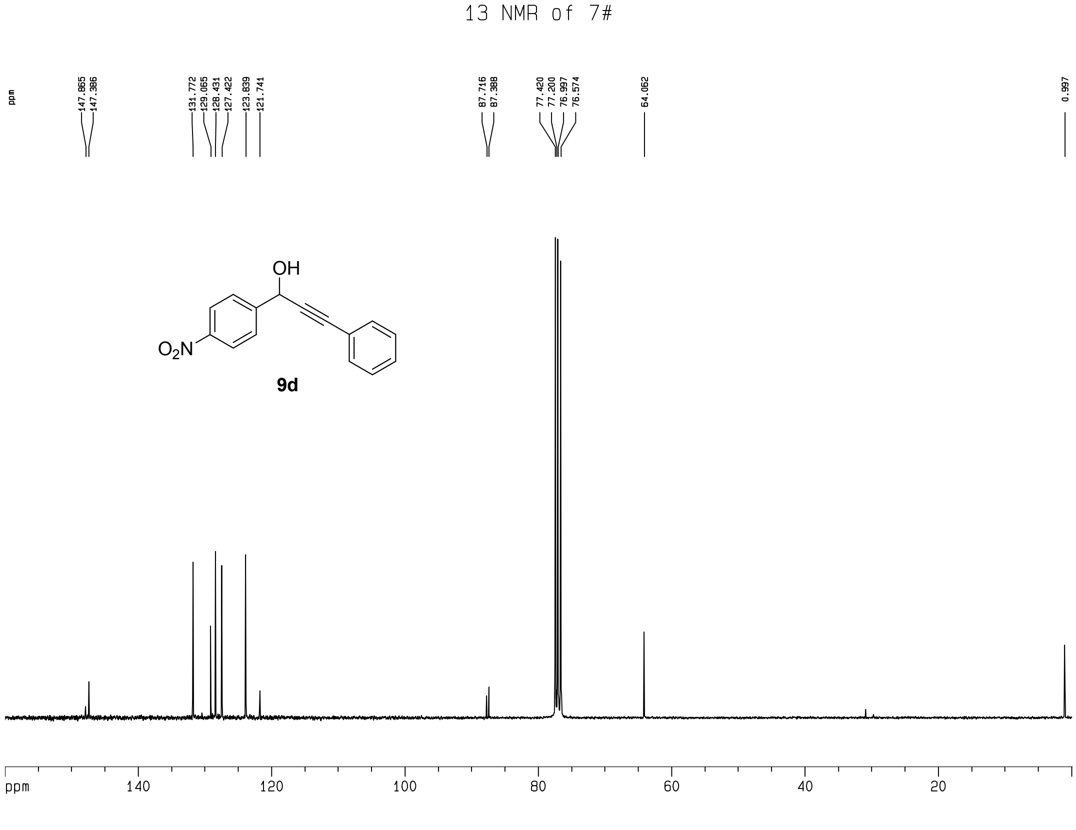

Current Data Parameters
NAME lizhiyuan-13C
EXPNO 24
PROCNO 1

F2 - Acquisition Parameters
Date_ 20090410
Time 7.46
INSTRUM dpx300
PROBHD 5 mm PABBO BB-
PULPROG zgdc30
TD 65536
SOLVENT CDCl3
NS 10240
DS 8
SWH 22675.736 Hz
FIDRES 0.346004 Hz
AQ 1.4451188 sec
RG 1024
DW 22.050 usec
DE 6.00 usec
TE 300.2 K
D1 1.50000000 sec
d11 0.03000000 sec
MCREST 0.00000000 sec
MCWRK 0.01500000 sec

======== CHANNEL f1 ========
NUC1 13C
P1 12.00 usec
PL1 0.90 dB
SFO1 75.4752958 MHz

======== CHANNEL f2 ========
CPDPRG2 waltz16
NUC2 1H
PCPD2 80.00 usec
PL2 -2.50 dB
PL12 18.00 dB
SFO2 300.1315007 MHz

F2 - Processing parameters
SI 32768
SF 75.4677492 MHz
WDW EM
SSB 0
LB 1.00 Hz
GB 0
PC 0.50

1D NMR plot parameters
CX 22.00 cm
CY 10.00 cm
F1P 160.000 ppm
F1 12074.84 Hz
F2P 0.000 ppm
F2 0.00 Hz
PPMCM 7.27273 ppm/cm
HZCM 548.85638 Hz/cm

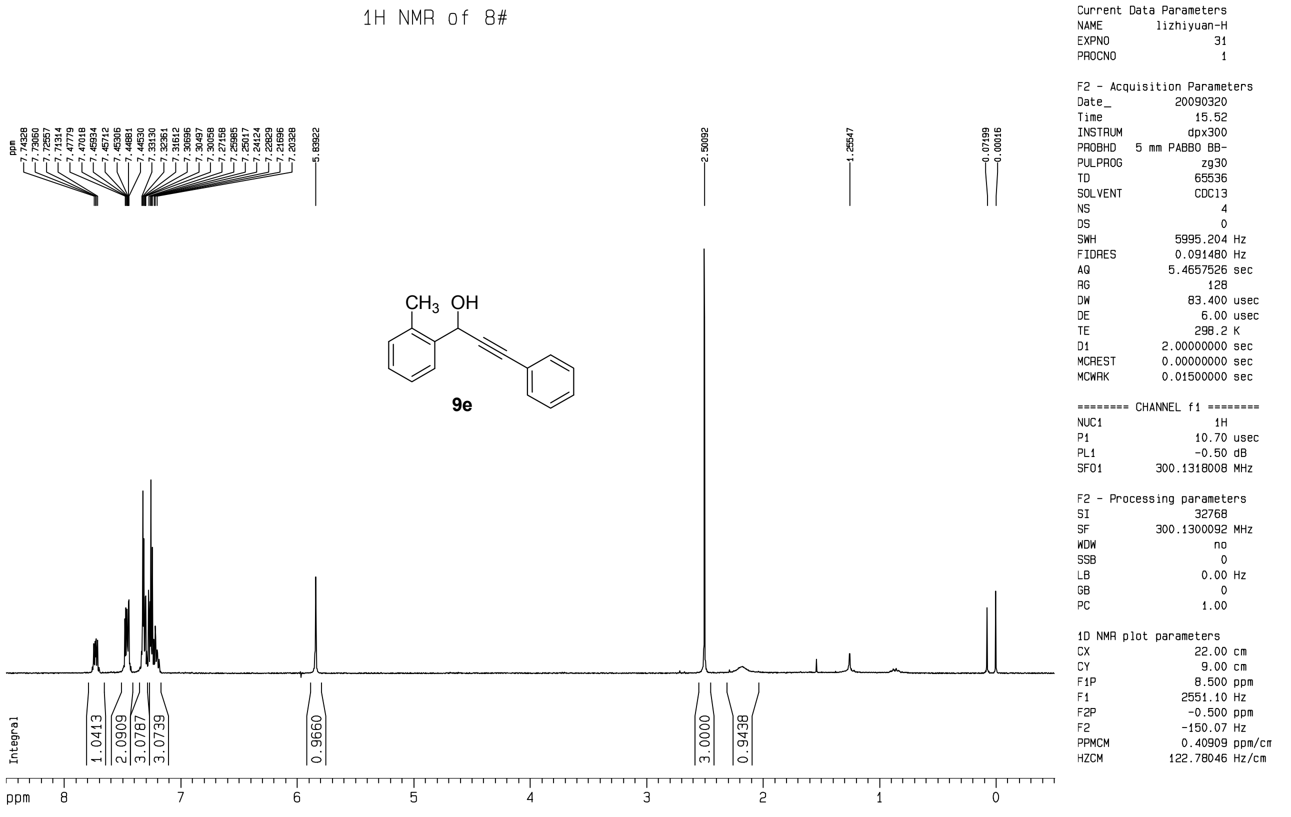
1H NMR of 8#
ppm
7.74328
7.73060
7.72957
7.71314
7.47779
7.47018
7.45934
7.45712
7.45306
7.44881
7.44530
7.33130
7.32361
7.31612
7.30696
7.30497
7.30058
7.27158
7.25985
7.25017
7.24124
7.22829
7.21696
7.20328
5.83922
2.50092
1.25547
0.07199
0.00016
CH3 OH
9e
Integral
1.0413
2.0909
3.0787
3.0739
0.9660
3.0000
0.9438
ppm 8 7 6 5 4 3 2 1 0
Current Data Parameters
NAME lizhiyuan-H
EXPNO 31
PROCNO 1
F2 - Acquisition Parameters
Date_ 20090320
Time 15.52
INSTRUM dpx300
PROBHD 5 mm PABBO BB-
PULPROG zg30
TD 65536
SOLVENT CDCl3
NS 4
DS 0
SWH 5995.204 Hz
FIDRES 0.091480 Hz
AQ 5.4657526 sec
RG 128
DW 83.400 usec
DE 6.00 usec
TE 298.2 K
D1 2.00000000 sec
MCREST 0.00000000 sec
MCWRK 0.01500000 sec
======== CHANNEL f1 ========
NUC1 1H
P1 10.70 usec
PL1 -0.50 dB
SFO1 300.1318008 MHz
F2 - Processing parameters
SI 32768
SF 300.1300092 MHz
WDW no
SSB 0
LB 0.00 Hz
GB 0
PC 1.00
1D NMR plot parameters
CX 22.00 cm
CY 9.00 cm
F1P 8.500 ppm
F1 2551.10 Hz
F2P -0.500 ppm
F2 -150.07 Hz
PPMCM 0.40909 ppm/cm
HZCM 122.78046 Hz/cm

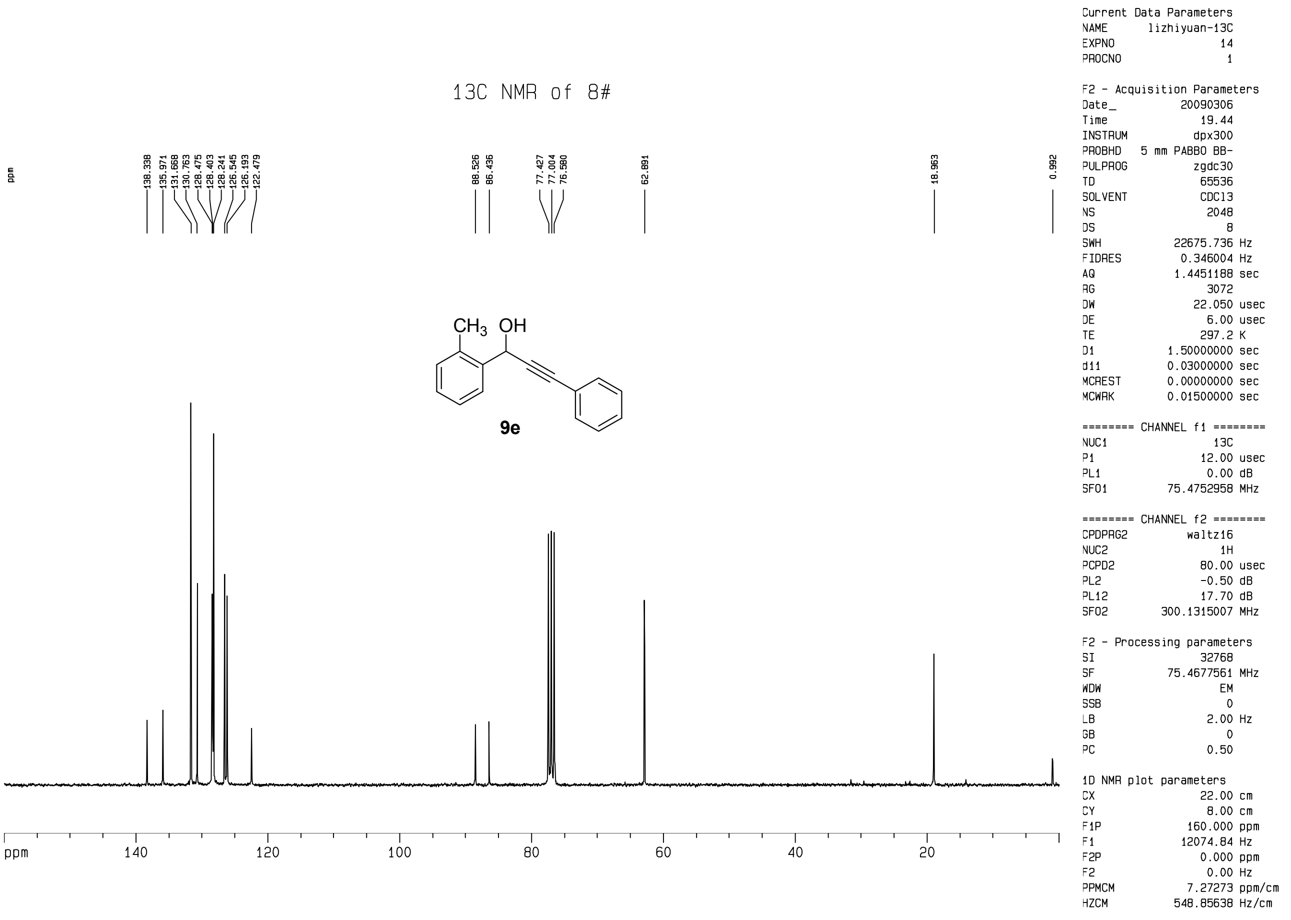
13C NMR of 8#
ppm
138.338
135.971
131.668
130.763
128.475
128.403
128.241
126.545
126.193
122.479
88.526
86.436
77.427
77.004
76.580
62.891
18.963
0.992
CH3 OH
9e
ppm 140 120 100 80 60 40 20
Current Data Parameters
NAME lizhiyuan-13C
EXPNO 14
PROCNO 1
F2 - Acquisition Parameters
Date_ 20090306
Time 19.44
INSTRUM dpx300
PROBHD 5 mm PABBO BB-
PULPROG zgdc30
TD 65536
SOLVENT CDCl3
NS 2048
DS 8
SWH 22675.736 Hz
FIDRES 0.346004 Hz
AQ 1.4451188 sec
RG 3072
DW 22.050 usec
DE 6.00 usec
TE 297.2 K
D1 1.50000000 sec
d11 0.03000000 sec
MCREST 0.00000000 sec
MCWRK 0.01500000 sec
======== CHANNEL f1 ========
NUC1 13C
P1 12.00 usec
PL1 0.00 dB
SFO1 75.4752958 MHz
======== CHANNEL f2 ========
CPDPRG2 waltz16
NUC2 1H
PCPD2 80.00 usec
PL2 -0.50 dB
PL12 17.70 dB
SFO2 300.1315007 MHz
F2 - Processing parameters
SI 32768
SF 75.4677561 MHz
WDW EM
SSB 0
LB 2.00 Hz
GB 0
PC 0.50
1D NMR plot parameters
CX 22.00 cm
CY 8.00 cm
F1P 160.000 ppm
F1 12074.84 Hz
F2P 0.000 ppm
F2 0.00 Hz
PPMCM 7.27273 ppm/cm
HZCM 548.85638 Hz/cm

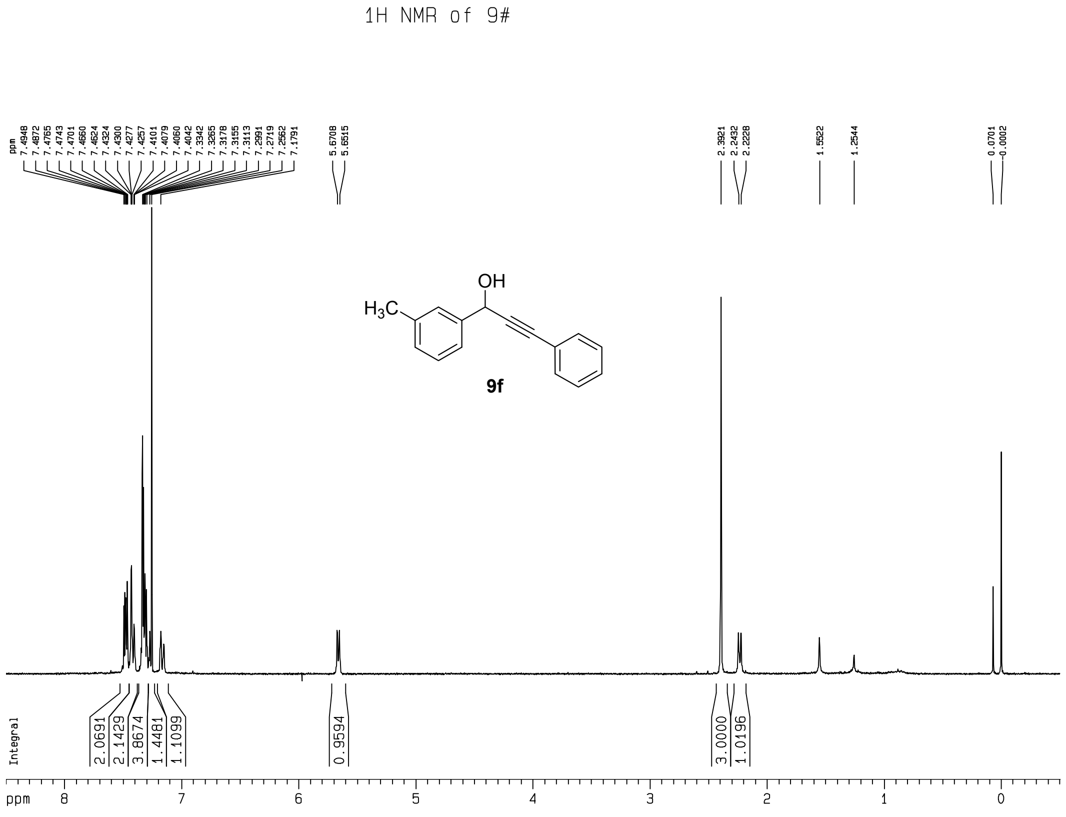

Current Data Parameters
NAME lizhiyuan-H
EXPNO 33
PROCNO 1

F2 - Acquisition Parameters
Date_ 20090323
Time 13.12
INSTRUM dpx300
PROBHD 5 mm PABBO BB-
PULPROG zg30
TD 65536
SOLVENT CDCl3
NS 8
DS 0
SWH 5995.204 Hz
FIDRES 0.091480 Hz
AQ 5.4657526 sec
RG 256
DW 83.400 usec
DE 6.00 usec
TE 0.0 K
D1 2.00000000 sec
MCREST 0.00000000 sec
MCWRK 0.01500000 sec

======== CHANNEL f1 ========
NUC1 1H
P1 10.70 usec
PL1 -0.50 dB
SFO1 300.1318008 MHz

F2 - Processing parameters
SI 32768
SF 300.1300074 MHz
WDW no
SSB 0
LB 0.00 Hz
GB 0
PC 1.00

1D NMR plot parameters
CX 22.00 cm
CY 10.00 cm
F1P 8.500 ppm
F1 2551.10 Hz
F2P -0.500 ppm
F2 -150.07 Hz
PPMCM 0.40909 ppm/cm
HZCM 122.78046 Hz/cm

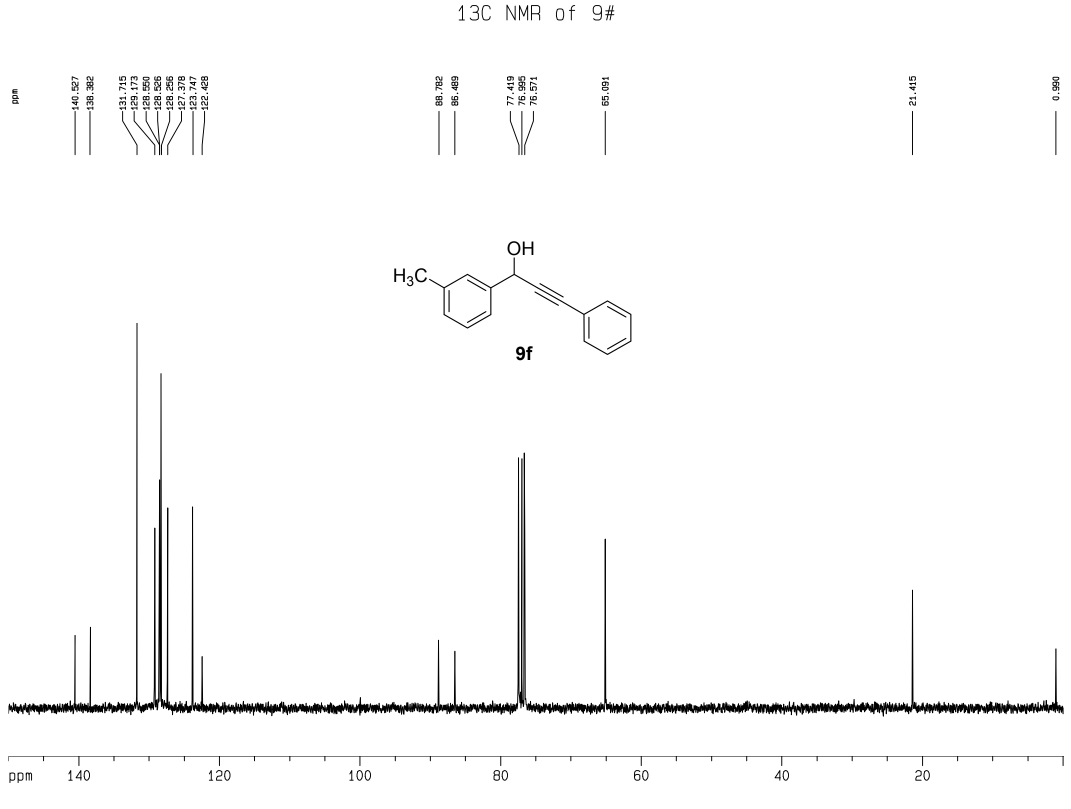

Current Data Parameters
NAME lizhiyuan-13C
EXPNO 21
PROCNO 1

F2 - Acquisition Parameters
Date_ 20090324
Time 17.54
INSTRUM dpx300
PROBHD 5 mm PABBO BB-
PULPROG zgdc30
TD 65536
SOLVENT CDCl3
NS 258
DS 8
SWH 22675.736 Hz
FIDRES 0.346004 Hz
AQ 1.4451188 sec
RG 2048
DW 22.050 usec
DE 6.00 usec
TE 297.2 K
D1 1.50000000 sec
d11 0.03000000 sec
MCREST 0.00000000 sec
MCWRK 0.01500000 sec

======== CHANNEL f1 ========
NUC1 13C
P1 12.00 usec
PL1 0.00 dB
SFO1 75.4752958 MHz

======== CHANNEL f2 ========
CPDPRG2 waltz16
NUC2 1H
PCPD2 80.00 usec
PL2 -0.50 dB
PL12 17.70 dB
SFO2 300.1315007 MHz

F2 - Processing parameters
SI 32768
SF 75.4677547 MHz
WDW EM
SSB 0
LB 1.00 Hz
GB 0
PC 0.50

1D NMR plot parameters
CX 22.00 cm
CY 8.00 cm
F1P 150.000 ppm
F1 11320.16 Hz
F2P -0.000 ppm
F2 -0.00 Hz
PPMCM 6.81818 ppm/cm
HZCM 514.55292 Hz/cm

1H NMR of 10#

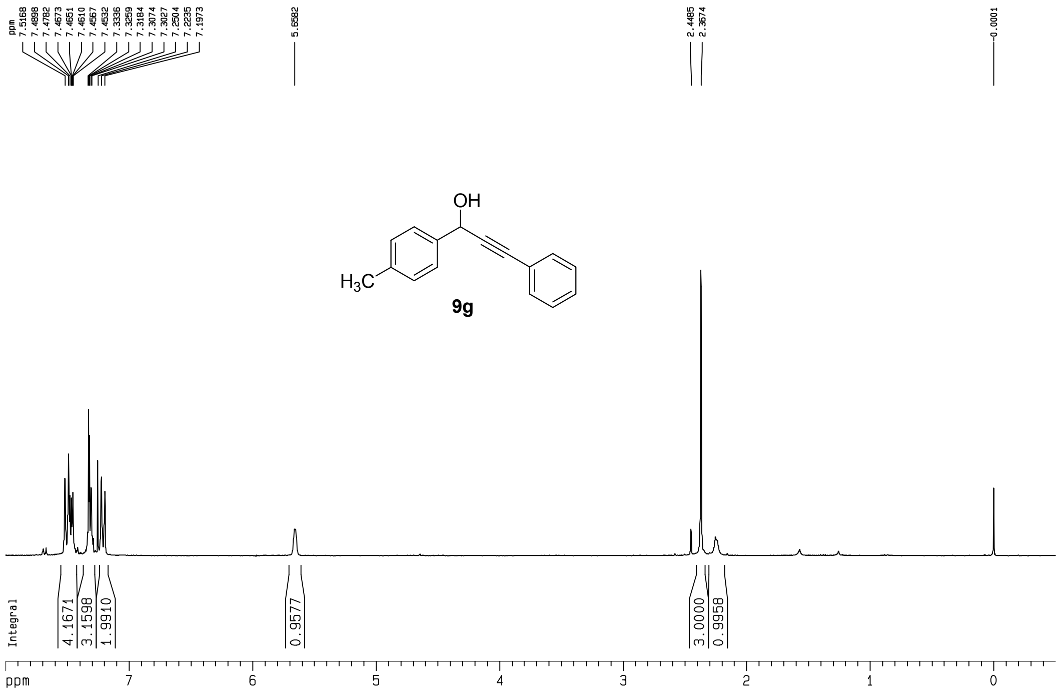

Current Data Parameters
NAME lizhiyuan-H
EXPNO 37
PROCNO 1

F2 - Acquisition Parameters
Date_ 20090407
Time 17.20
INSTRUM dpx300
PROBHD 5 mm PABBO BB-
PULPROG zg30
TD 65536
SOLVENT CDCl3
NS 4
DS 0
SWH 5995.204 Hz
FIDRES 0.091480 Hz
AQ 5.4657526 sec
RG 258
DW 83.400 usec
DE 6.00 usec
TE 297.2 K
D1 2.00000000 sec
MCREST 0.00000000 sec
MCWRK 0.01500000 sec

======== CHANNEL f1 ========
NUC1 1H
P1 10.00 usec
PL1 -2.50 dB
SFO1 300.1318008 MHz

F2 - Processing parameters
SI 32768
SF 300.1300085 MHz
WDW EM
SSB 0
LB 0.10 Hz
GB 0
PC 1.00

1D NMR plot parameters
CX 22.00 cm
CY 6.00 cm
F1P 8.000 ppm
F1 2401.04 Hz
F2P -0.500 ppm
F2 -150.06 Hz
PPMCM 0.38636 ppm/cm
HZCM 115.95932 Hz/cm

13C NMR of 10#

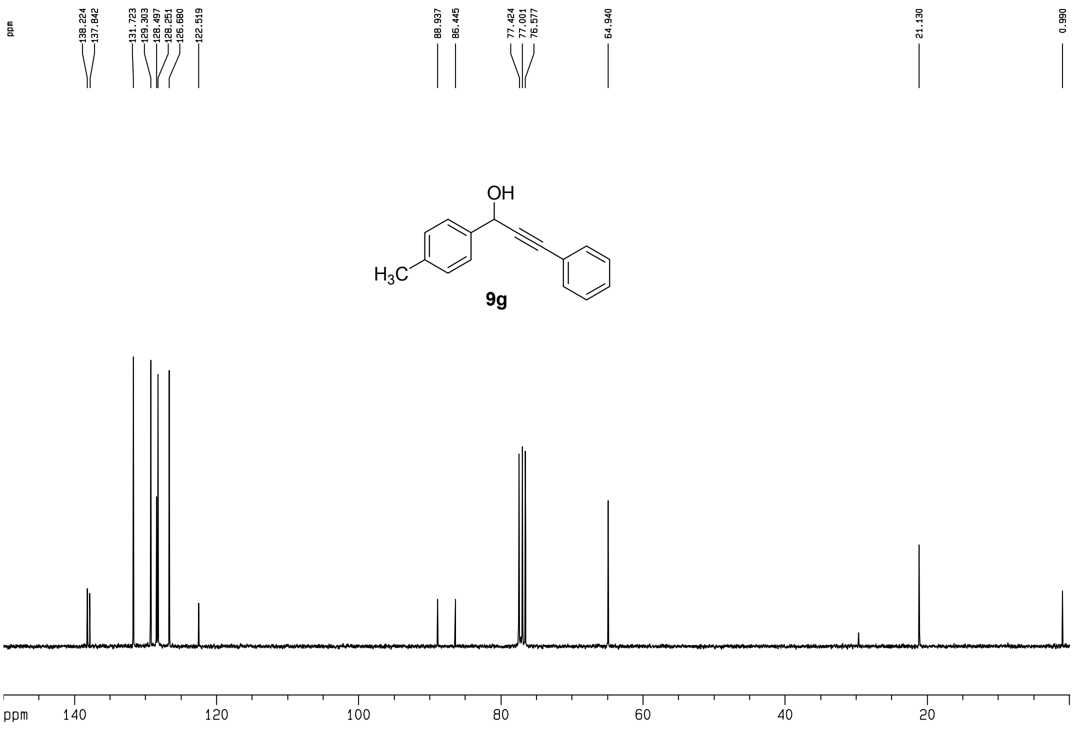

Current Data Parameters
NAME lizhiyuan-13C
EXPNO 27
PROCNO 1

F2 - Acquisition Parameters
Date_ 20090411
Time 18.35
INSTRUM dpx300
PROBHD 5 mm PABBO BB-
PULPROG zgdc30
TD 65536
SOLVENT CDCl3
NS 566
DS 8
SWH 22675.736 Hz
FIDRES 0.346004 Hz
AQ 1.4451188 sec
RG 6144
DW 22.050 usec
DE 6.00 usec
TE 300.2 K
D1 1.50000000 sec
d11 0.03000000 sec
MCREST 0.00000000 sec
MCWRK 0.01500000 sec

======== CHANNEL f1 ========
NUC1 13C
P1 12.00 usec
PL1 0.90 dB
SFO1 75.4752958 MHz

======== CHANNEL f2 ========
CPDPRG2 waltz16
NUC2 1H
PCPD2 80.00 usec
PL2 -2.50 dB
PL12 18.00 dB
SFO2 300.1315007 MHz

F2 - Processing parameters
SI 32768
SF 75.4677527 MHz
WDW EM
SSB 0
LB 1.00 Hz
GB 0
PC 0.50

1D NMR plot parameters
CX 22.00 cm
CY 6.00 cm
F1P 150.000 ppm
F1 11320.16 Hz
F2P -0.000 ppm
F2 -0.00 Hz
PPMCM 6.81818 ppm/cm
HZCM 514.55286 Hz/cm

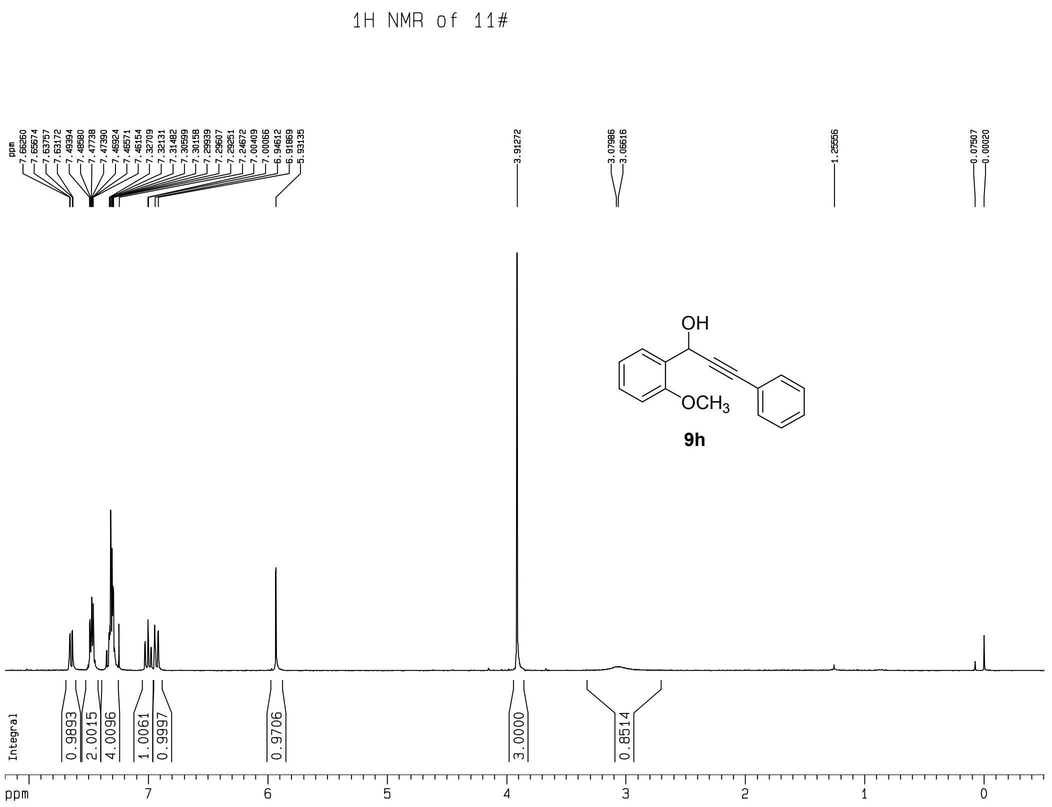

Current Data Parameters	
NAME	lizhiyuan-H
EXPNO	39
PROCNO	1
**F2 - Acquisition Parameters**	
Date_	20090409
Time	11.00
INSTRUM	dpx300
PROBHD	5 mm PABBO BB-
PULPROG	zg30
TD	65536
SOLVENT	CDCl3
NS	4
DS	0
SWH	5995.204 Hz
FIDRES	0.091480 Hz
AQ	5.4657526 sec
RG	128
DW	83.400 usec
DE	6.00 usec
TE	297.2 K
D1	2.00000000 sec
MCREST	0.00000000 sec
MCWRK	0.01500000 sec
======== CHANNEL f1 ========	
NUC1	1H
P1	10.00 usec
PL1	-2.50 dB
SFO1	300.1318008 MHz
**F2 - Processing parameters**	
SI	32768
SF	300.1300102 MHz
WDW	no
SSB	0
LB	0.00 Hz
GB	0
PC	1.00
**1D NMR plot parameters**	
CX	22.00 cm
CY	9.00 cm
F1P	8.200 ppm
F1	2461.07 Hz
F2P	-0.500 ppm
F2	-150.07 Hz
PPMCM	0.39545 ppm/cm
HZCM	118.68777 Hz/cm

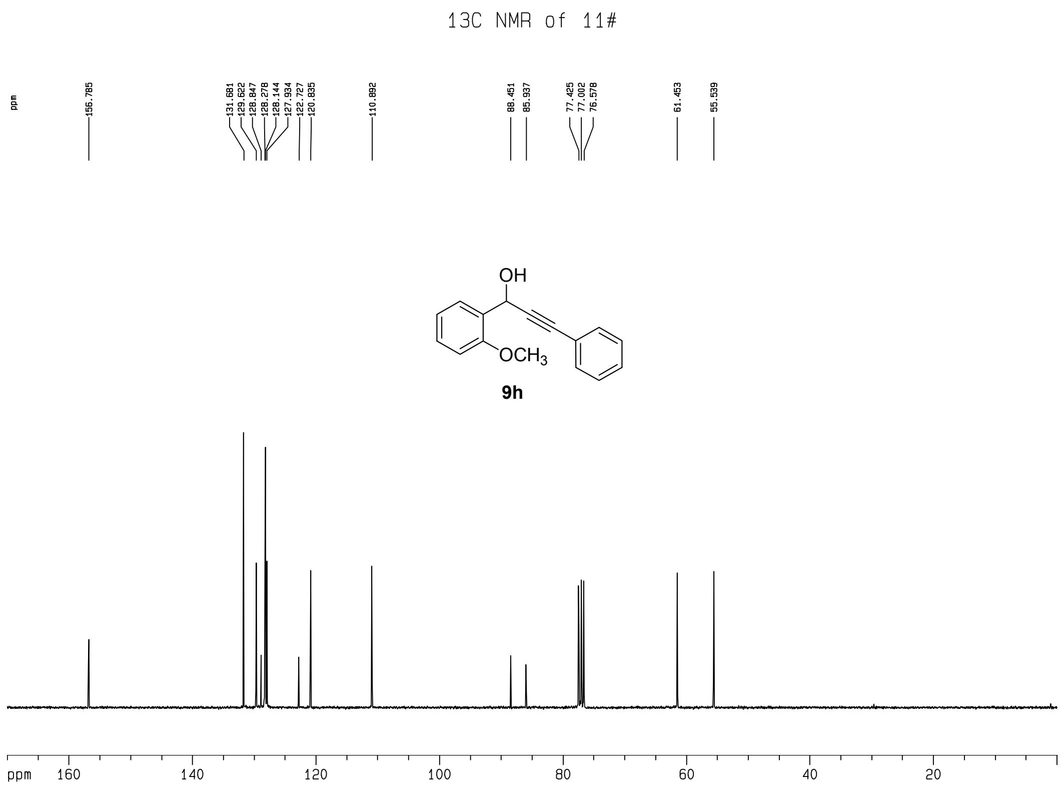

Current Data Parameters	
NAME	lizhiyuan-13C
EXPNO	26
PROCNO	1
**F2 - Acquisition Parameters**	
Date_	20090411
Time	17.47
INSTRUM	dpx300
PROBHD	5 mm PABBO BB-
PULPROG	zgdc30
TD	65536
SOLVENT	CDCl3
NS	802
DS	8
SWH	22675.736 Hz
FIDRES	0.346004 Hz
AQ	1.4451188 sec
RG	9216
DW	22.050 usec
DE	6.00 usec
TE	300.2 K
D1	1.50000000 sec
d11	0.03000000 sec
MCREST	0.00000000 sec
MCWRK	0.01500000 sec
======== CHANNEL f1 ========	
NUC1	13C
P1	12.00 usec
PL1	0.90 dB
SFO1	75.4752958 MHz
======== CHANNEL f2 ========	
CPDPRG2	waltz16
NUC2	1H
PCPD2	80.00 usec
PL2	-2.50 dB
PL12	18.00 dB
SFO2	300.1315007 MHz
**F2 - Processing parameters**	
SI	32768
SF	75.4677582 MHz
WDW	EM
SSB	0
LB	1.00 Hz
GB	0
PC	0.50
**1D NMR plot parameters**	
CX	22.00 cm
CY	6.00 cm
F1P	170.000 ppm
F1	12829.52 Hz
F2P	-0.000 ppm
F2	-0.00 Hz
PPMCM	7.72727 ppm/cm
HZCM	583.15991 Hz/cm

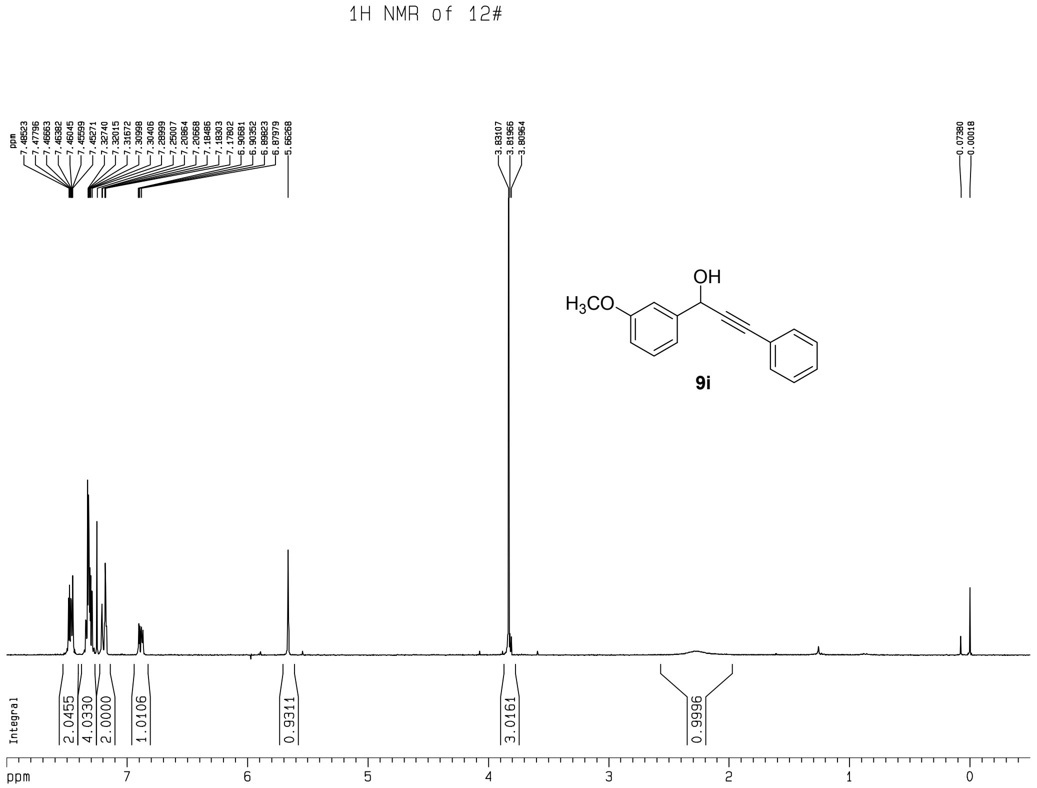

Current Data Parameters
NAME lizhiyuan-H
EXPNO 23
PROCNO 1

F2 - Acquisition Parameters
Date_ 20090319
Time 20.41
INSTRUM dpx300
PROBHD 5 mm PABBO BB-
PULPROG zg30
TD 65536
SOLVENT CDCl3
NS 4
DS 0
SWH 5995.204 Hz
FIDRES 0.091480 Hz
AQ 5.4657526 sec
RG 128
DW 83.400 usec
DE 6.00 usec
TE 298.2 K
D1 2.00000000 sec
MCREST 0.00000000 sec
MCWRK 0.01500000 sec

======== CHANNEL f1 ========
NUC1 1H
P1 10.70 usec
PL1 -0.50 dB
SFO1 300.1318008 MHz

F2 - Processing parameters
SI 32768
SF 300.1300092 MHz
WDW no
SSB 0
LB 0.00 Hz
GB 0
PC 1.00

1D NMR plot parameters
CX 22.00 cm
CY 16.00 cm
F1P 8.000 ppm
F1 2401.04 Hz
F2P -0.500 ppm
F2 -150.06 Hz
PPMCM 0.38636 ppm/cm
HZCM 115.95932 Hz/cm

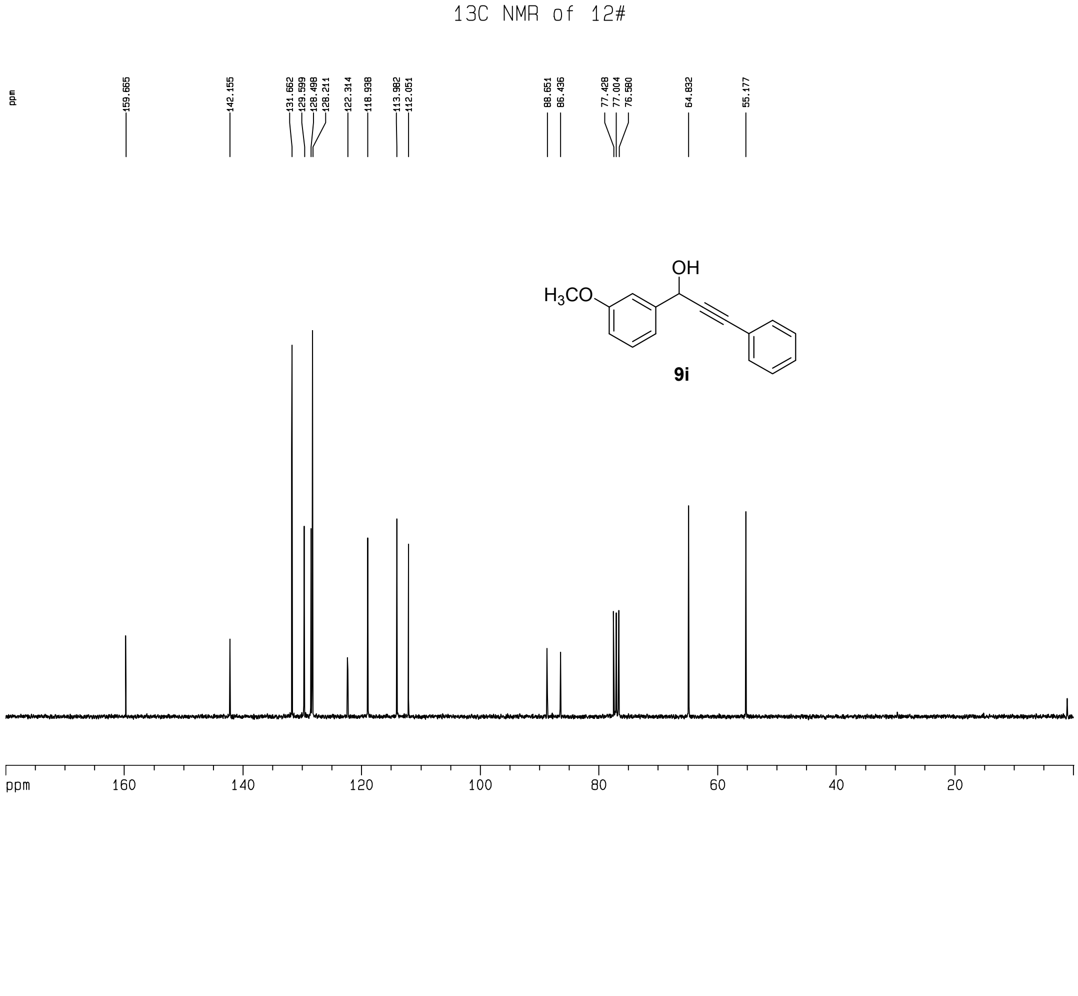

Current Data Parameters
NAME lizhiyuan-13C
EXPNO 13
PROCNO 1

F2 - Acquisition Parameters
Date_ 20090306
Time 19.27
INSTRUM dpx300
PROBHD 5 mm PABBO BB-
PULPROG zgdc30
TD 65536
SOLVENT CDCl3
NS 236
DS 8
SWH 22675.736 Hz
FIDRES 0.346004 Hz
AQ 1.4451188 sec
RG 6144
DW 22.050 usec
DE 6.00 usec
TE 297.2 K
D1 1.50000000 sec
d11 0.03000000 sec
MCREST 0.00000000 sec
MCWRK 0.01500000 sec

======== CHANNEL f1 ========
NUC1 13C
P1 12.00 usec
PL1 0.00 dB
SFO1 75.4752958 MHz

======== CHANNEL f2 ========
CPDPRG2 waltz16
NUC2 1H
PCPD2 80.00 usec
PL2 -0.50 dB
PL12 17.70 dB
SFO2 300.1315007 MHz

F2 - Processing parameters
SI 32768
SF 75.4677603 MHz
WDW EM
SSB 0
LB 1.00 Hz
GB 0
PC 0.50

1D NMR plot parameters
CX 22.00 cm
CY 8.00 cm
F1P 180.000 ppm
F1 13584.20 Hz
F2P 0.000 ppm
F2 0.00 Hz
PPMCM 8.18182 ppm/cm
HZCM 617.46350 Hz/cm

1H NMR of 13#

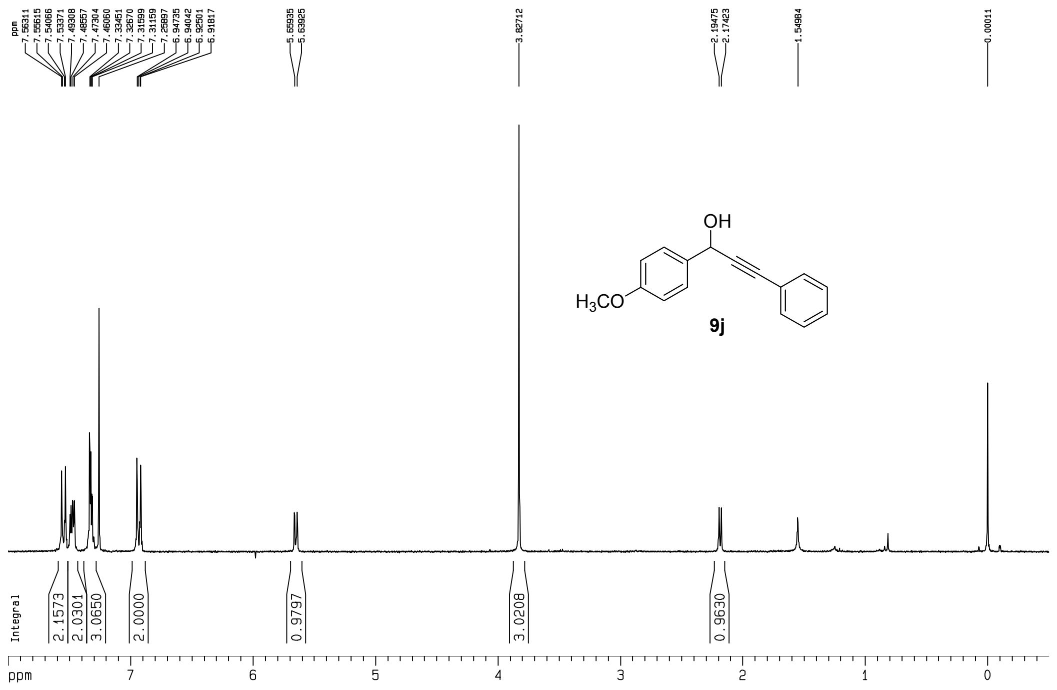

Current Data Parameters
NAME lizhiyuan-H
EXPNO 38
PROCNO 1

F2 - Acquisition Parameters
Date_ 20090407
Time 17.25
INSTRUM dpx300
PROBHD 5 mm PABBO BB-
PULPROG zg30
TD 65536
SOLVENT CDCl3
NS 4
DS 0
SWH 5995.204 Hz
FIDRES 0.091480 Hz
AQ 5.4657526 sec
RG 258
DW 83.400 usec
DE 6.00 usec
TE 297.2 K
D1 2.00000000 sec
MCREST 0.00000000 sec
MCWRK 0.01500000 sec

======== CHANNEL f1 ========
NUC1 1H
P1 10.00 usec
PL1 -2.50 dB
SFO1 300.1318008 MHz

F2 - Processing parameters
SI 32768
SF 300.1300060 MHz
WDW EM
SSB 0
LB 0.10 Hz
GB 0
PC 1.00

1D NMR plot parameters
CX 22.00 cm
CY 9.00 cm
F1P 8.000 ppm
F1 2401.04 Hz
F2P -0.500 ppm
F2 -150.06 Hz
PPMCM 0.38636 ppm/cm
HZCM 115.95932 Hz/cm

13C NMR of 13#

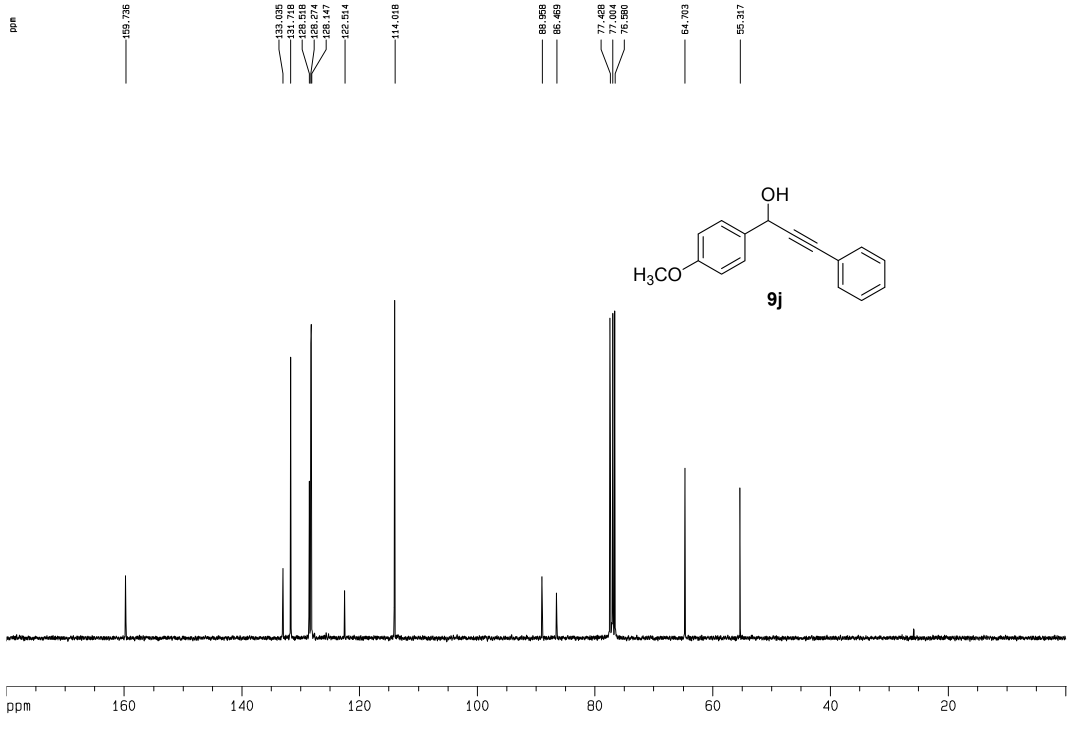

Current Data Parameters
NAME lizhiyuan-13C
EXPNO 23
PROCNO 1

F2 - Acquisition Parameters
Date_ 20090408
Time 17.43
INSTRUM dpx300
PROBHD 5 mm PABBO BB-
PULPROG zgdc30
TD 65536
SOLVENT CDCl3
NS 1370
DS 8
SWH 22675.736 Hz
FIDRES 0.346004 Hz
AQ 1.4451188 sec
RG 3072
DW 22.050 usec
DE 6.00 usec
TE 299.2 K
D1 1.50000000 sec
d11 0.03000000 sec
MCREST 0.00000000 sec
MCWRK 0.01500000 sec

======== CHANNEL f1 ========
NUC1 13C
P1 12.00 usec
PL1 0.90 dB
SFO1 75.4752958 MHz

======== CHANNEL f2 ========
CPDPRG2 waltz16
NUC2 1H
PCPD2 80.00 usec
PL2 -2.50 dB
PL12 18.00 dB
SFO2 300.1315007 MHz

F2 - Processing parameters
SI 32768
SF 75.4677513 MHz
WDW EM
SSB 0
LB 1.00 Hz
GB 0
PC 0.50

1D NMR plot parameters
CX 22.00 cm
CY 7.00 cm
F1P 180.000 ppm
F1 13584.20 Hz
F2P 0.000 ppm
F2 0.00 Hz
PPMCM 8.18182 ppm/cm
HZCM 617.46344 Hz/cm

1H NMR of 15#

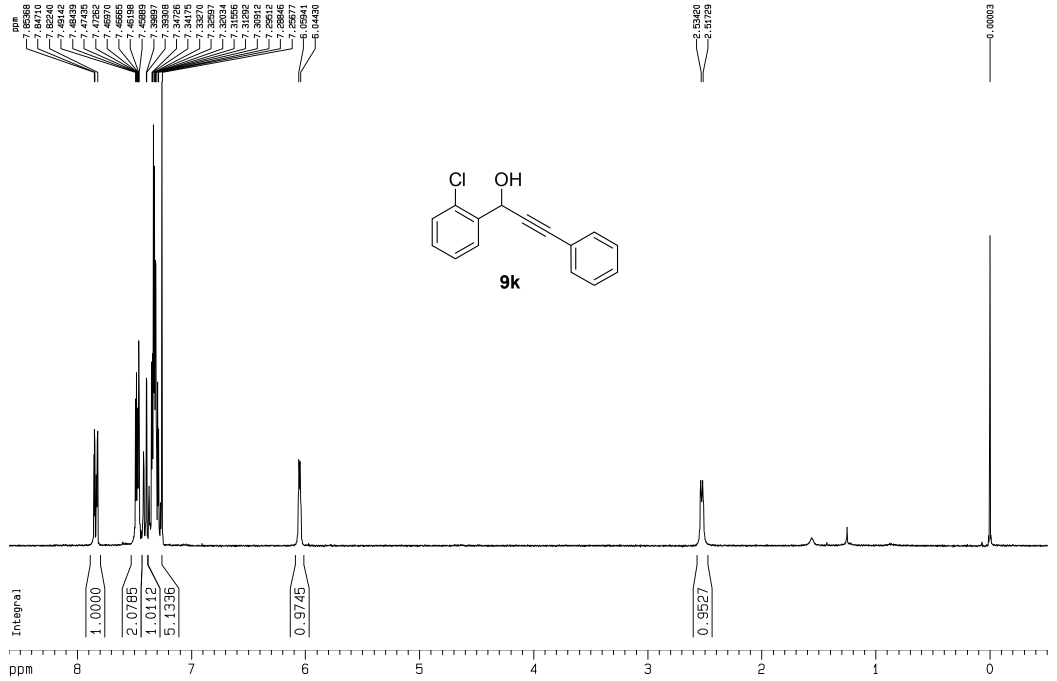

Current Data Parameters
NAME lizhiyuan-H
EXPNO 19
PROCNO 1

F2 - Acquisition Parameters
Date_ 20090306
Time 12.57
INSTRUM dpx300
PROBHD 5 mm PABBO BB-
PULPROG zg30
TD 65536
SOLVENT CDCl3
NS 4
DS 0
SWH 5995.204 Hz
FIDRES 0.091480 Hz
AQ 5.4657526 sec
RG 258
DW 83.400 usec
DE 6.00 usec
TE 296.2 K
D1 2.00000000 sec
MCREST 0.00000000 sec
MCWRK 0.01500000 sec

======== CHANNEL f1 ========
NUC1 1H
P1 10.70 usec
PL1 -0.50 dB
SFO1 300.1318008 MHz

F2 - Processing parameters
SI 32768
SF 300.1300072 MHz
WDW EM
SSB 0
LB 0.10 Hz
GB 0
PC 1.00

1D NMR plot parameters
CX 22.00 cm
CY 10.00 cm
F1P 8.600 ppm
F1 2581.12 Hz
F2P -0.500 ppm
F2 -150.07 Hz
PPMCM 0.41364 ppm/cm
HZCM 124.14469 Hz/cm

13C NMR of 15#

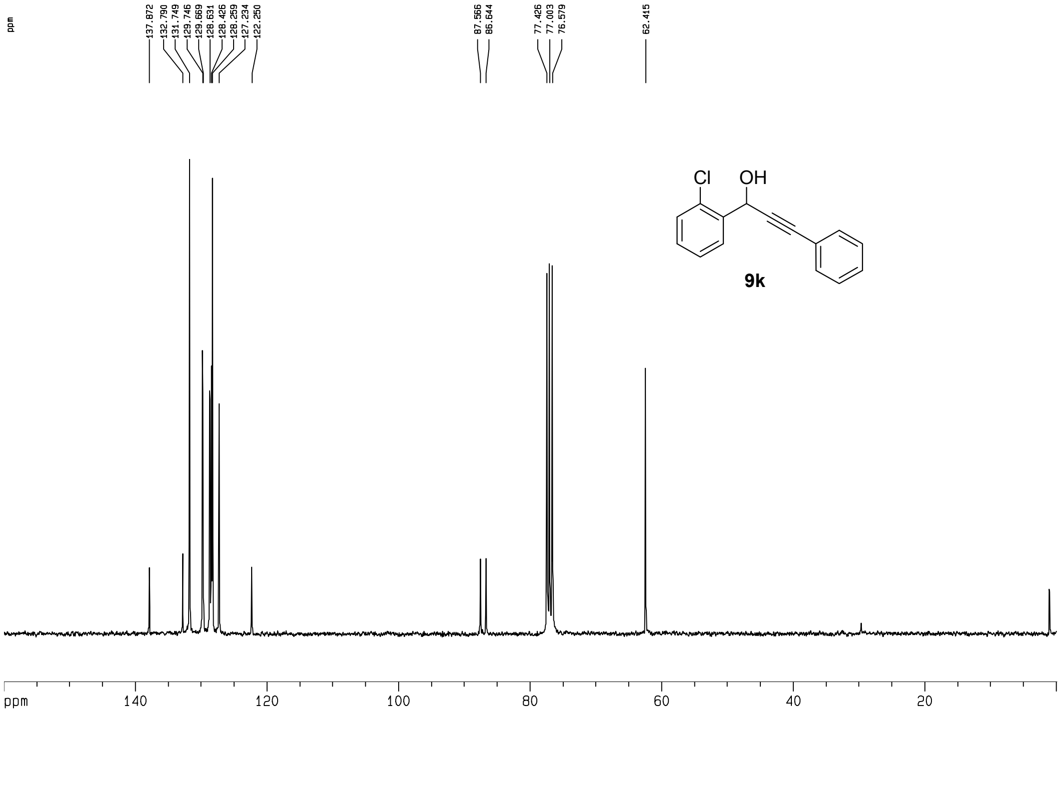

Current Data Parameters
NAME lizhiyuan-13C
EXPNO 16
PROCNO 1

F2 - Acquisition Parameters
Date_ 20090307
Time 11.54
INSTRUM dpx300
PROBHD 5 mm PABBO BB-
PULPROG zgdc30
TD 65536
SOLVENT CDCl3
NS 2048
DS 8
SWH 22675.736 Hz
FIDRES 0.346004 Hz
AQ 1.4451188 sec
RG 8192
DW 22.050 usec
DE 6.00 usec
TE 297.2 K
D1 1.50000000 sec
d11 0.03000000 sec
MCREST 0.00000000 sec
MCWRK 0.01500000 sec

======== CHANNEL f1 ========
NUC1 13C
P1 12.00 usec
PL1 0.00 dB
SFO1 75.4752958 MHz

======== CHANNEL f2 ========
CPDPRG2 waltz16
NUC2 1H
PCPD2 80.00 usec
PL2 -0.50 dB
PL12 17.70 dB
SFO2 300.1315007 MHz

F2 - Processing parameters
SI 32768
SF 75.4677540 MHz
WDW EM
SSB 0
LB 3.00 Hz
GB 0
PC 0.50

1D NMR plot parameters
CX 22.00 cm
CY 10.00 cm
F1P 160.000 ppm
F1 12074.84 Hz
F2P 0.000 ppm
F2 0.00 Hz
PPMCM 7.27273 ppm/cm
HZCM 548.85638 Hz/cm

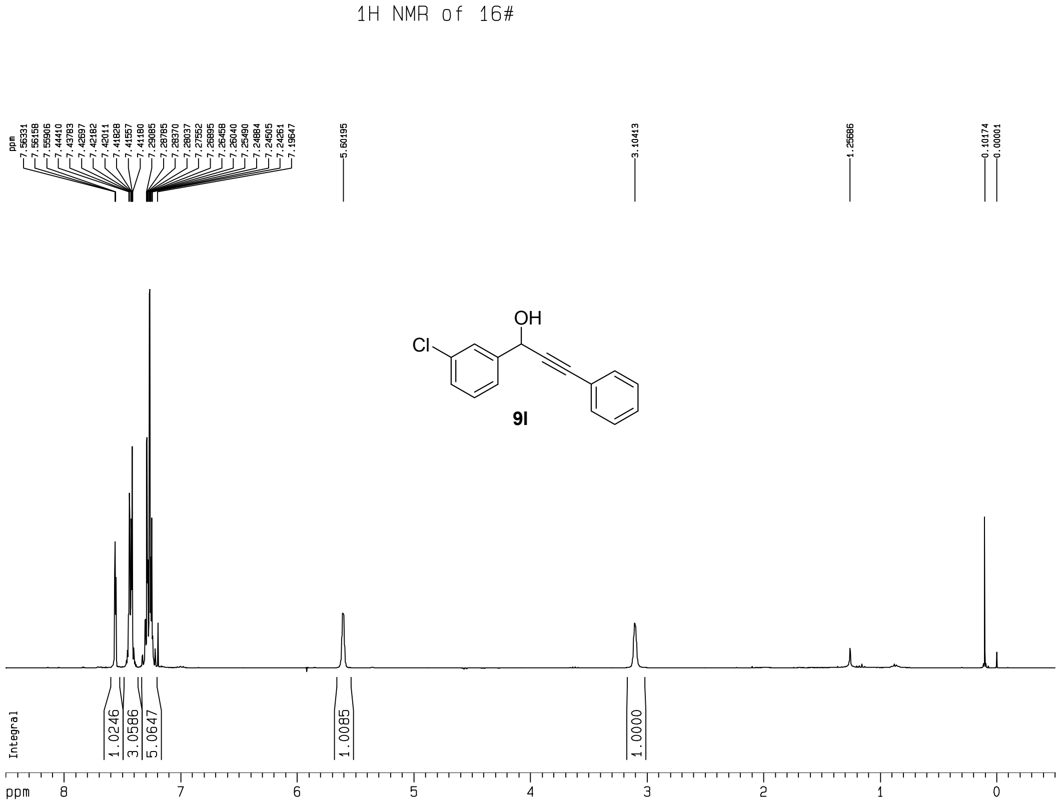

Current Data Parameters
NAME lizhiyuan-H
EXPNO 34
PROCNO 1

F2 - Acquisition Parameters
Date_ 20090324
Time 14.18
INSTRUM dpx300
PROBHD 5 mm PABBO BB-
PULPROG zg30
TD 65536
SOLVENT CDCl3
NS 4
DS 0
SWH 5995.204 Hz
FIDRES 0.091480 Hz
AQ 5.4657526 sec
RG 32
DW 83.400 usec
DE 6.00 usec
TE 297.2 K
D1 2.00000000 sec
MCREST 0.00000000 sec
MCWRK 0.01500000 sec

======== CHANNEL f1 ========
NUC1 1H
P1 10.70 usec
PL1 -0.50 dB
SFO1 300.1318008 MHz

F2 - Processing parameters
SI 32768
SF 300.1300252 MHz
WDW no
SSB 0
LB 0.00 Hz
GB 0
PC 1.00

1D NMR plot parameters
CX 22.00 cm
CY 8.00 cm
F1P 8.500 ppm
F1 2551.11 Hz
F2P -0.500 ppm
F2 -150.07 Hz
PPMCM 0.40909 ppm/cm
HZCM 122.78047 Hz/cm

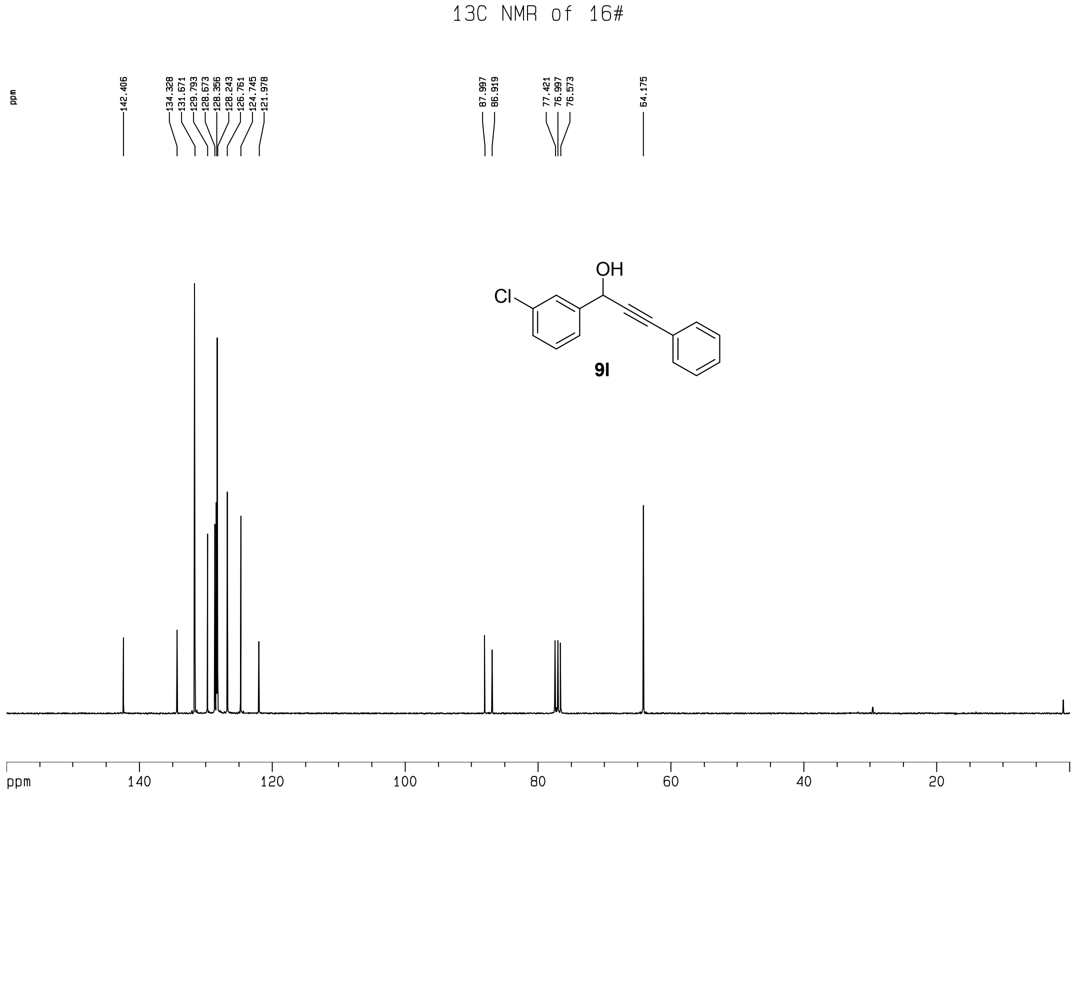

Current Data Parameters
NAME lizhiyuan-13C
EXPNO 20
PROCNO 1

F2 - Acquisition Parameters
Date_ 20090324
Time 11.45
INSTRUM dpx300
PROBHD 5 mm PABBO BB-
PULPROG zgdc30
TD 65536
SOLVENT CDCl3
NS 2024
DS 8
SWH 22675.736 Hz
FIDRES 0.346004 Hz
AQ 1.4451188 sec
RG 2048
DW 22.050 usec
DE 6.00 usec
TE 297.2 K
D1 1.50000000 sec
d11 0.03000000 sec
MCREST 0.00000000 sec
MCWRK 0.01500000 sec

======== CHANNEL f1 ========
NUC1 13C
P1 12.00 usec
PL1 0.00 dB
SFO1 75.4752958 MHz

======== CHANNEL f2 ========
CPDPRG2 waltz16
NUC2 1H
PCPD2 80.00 usec
PL2 -0.50 dB
PL12 17.70 dB
SFO2 300.1315007 MHz

F2 - Processing parameters
SI 32768
SF 75.4677630 MHz
WDW EM
SSB 0
LB 1.00 Hz
GB 0
PC 0.50

1D NMR plot parameters
CX 22.00 cm
CY 9.00 cm
F1P 160.000 ppm
F1 12074.84 Hz
F2P 0.000 ppm
F2 0.00 Hz
PPMCM 7.27273 ppm/cm
HZCM 548.85645 Hz/cm

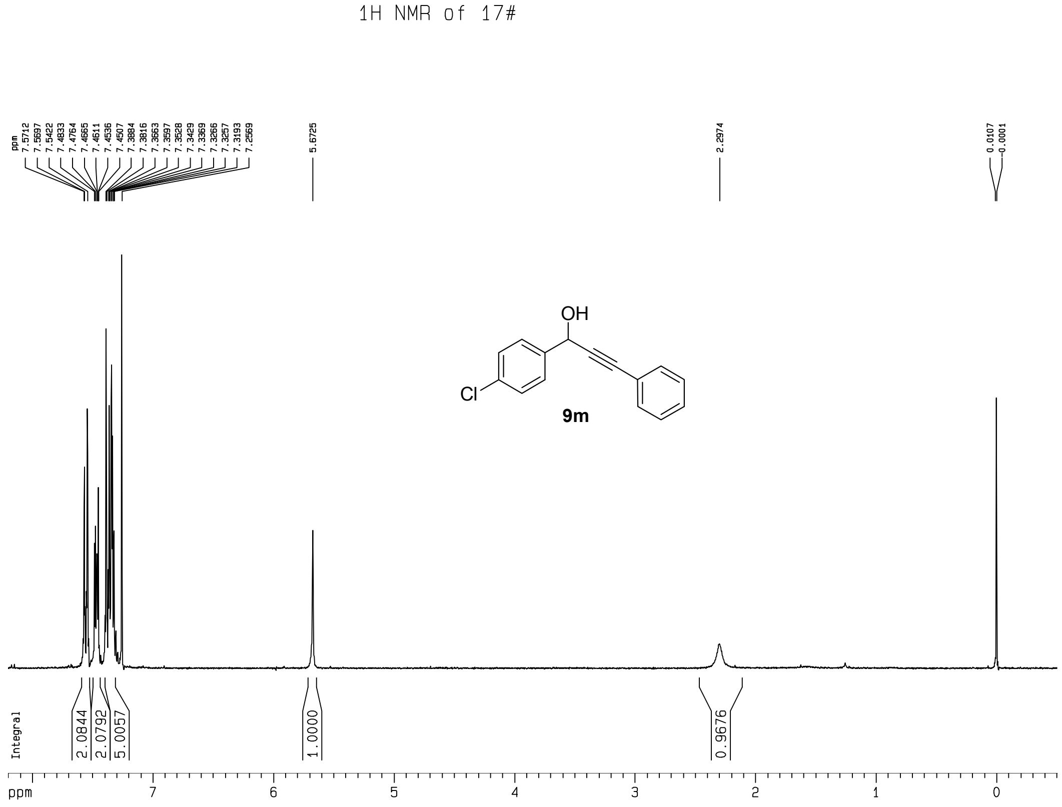

Current Data Parameters
NAME lizhiyuan-H
EXPNO 22
PROCNO 1

F2 - Acquisition Parameters
Date_ 20090306
Time 13.17
INSTRUM dpx300
PROBHD 5 mm PABBO BB-
PULPROG zg30
TD 65536
SOLVENT CDCl3
NS 4
DS 0
SWH 5995.204 Hz
FIDRES 0.091480 Hz
AQ 5.4657526 sec
RG 258
DW 83.400 usec
DE 6.00 usec
TE 295.2 K
D1 2.00000000 sec
MCREST 0.00000000 sec
MCWRK 0.01500000 sec

======== CHANNEL f1 ========
NUC1 1H
P1 10.70 usec
PL1 -0.50 dB
SFO1 300.1318008 MHz

F2 - Processing parameters
SI 32768
SF 300.1300072 MHz
WDW EM
SSB 0
LB 0.10 Hz
GB 0
PC 1.00

1D NMR plot parameters
CX 22.00 cm
CY 9.00 cm
F1P 8.200 ppm
F1 2461.07 Hz
F2P -0.500 ppm
F2 -150.07 Hz
PPMCM 0.39545 ppm/cm
HZCM 118.68777 Hz/cm

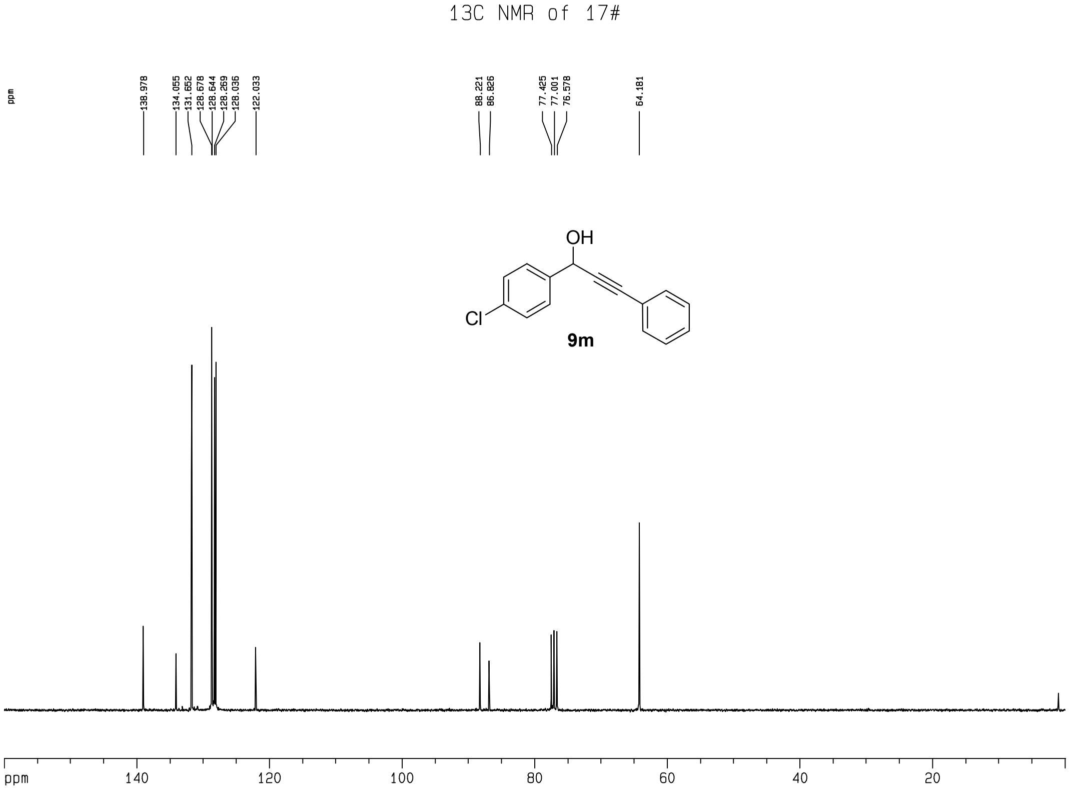

Current Data Parameters
NAME lizhiyuan-13C
EXPNO 17
PROCNO 1

F2 - Acquisition Parameters
Date_ 20090307
Time 15.21
INSTRUM dpx300
PROBHD 5 mm PABBO BB-
PULPROG zgdc30
TD 65536
SOLVENT CDCl3
NS 482
DS 8
SWH 22675.736 Hz
FIDRES 0.346004 Hz
AQ 1.4451188 sec
RG 7168
DW 22.050 usec
DE 6.00 usec
TE 297.2 K
D1 1.50000000 sec
d11 0.03000000 sec
MCREST 0.00000000 sec
MCWRK 0.01500000 sec

======== CHANNEL f1 ========
NUC1 13C
P1 12.00 usec
PL1 0.00 dB
SFO1 75.4752958 MHz

======== CHANNEL f2 ========
CPDPRG2 waltz16
NUC2 1H
PCPD2 80.00 usec
PL2 -0.50 dB
PL12 17.70 dB
SFO2 300.1315007 MHz

F2 - Processing parameters
SI 32768
SF 75.4677610 MHz
WDW EM
SSB 0
LB 1.00 Hz
GB 0
PC 0.50

1D NMR plot parameters
CX 22.00 cm
CY 8.00 cm
F1P 160.000 ppm
F1 12074.84 Hz
F2P 0.000 ppm
F2 0.00 Hz
PPMCM 7.27273 ppm/cm
HZCM 548.85638 Hz/cm

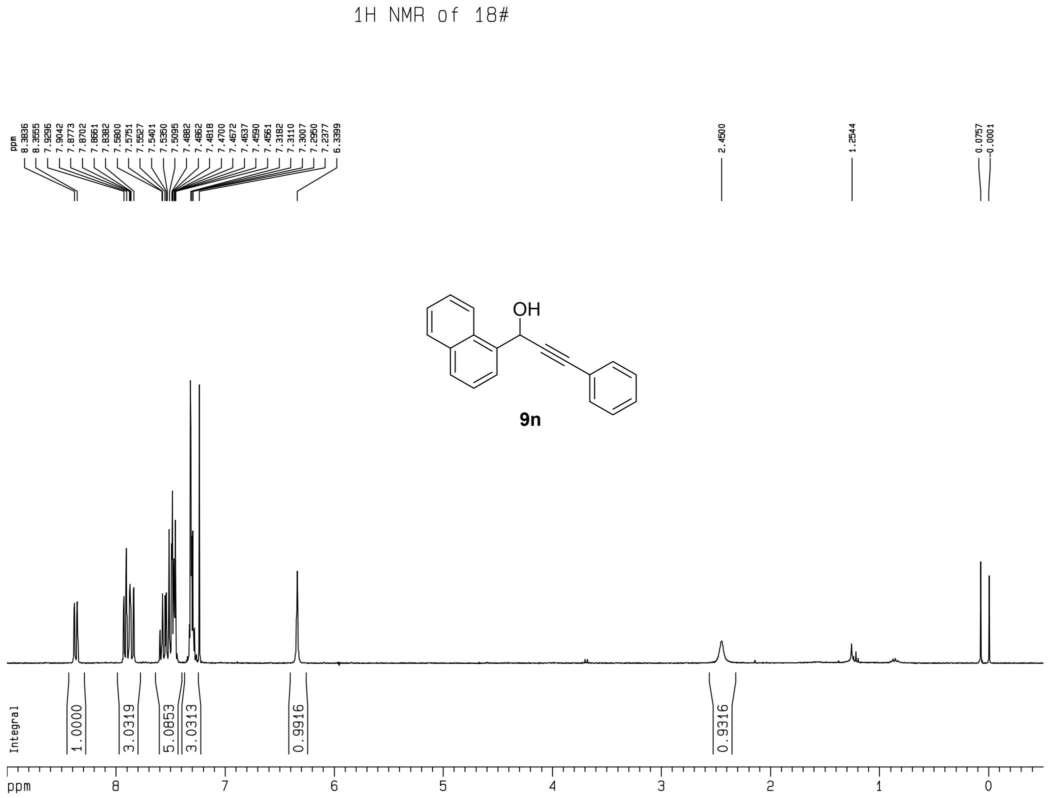

Current Data Parameters
NAME lizhiyuan-H
EXPNO 28
PROCNO 1

F2 - Acquisition Parameters
Date_ 20090320
Time 15.40
INSTRUM dpx300
PROBHD 5 mm PABBO BB-
PULPROG zg30
TD 65536
SOLVENT CDCl3
NS 4
DS 0
SWH 5995.204 Hz
FIDRES 0.091480 Hz
AQ 5.4657526 sec
RG 128
DW 83.400 usec
DE 6.00 usec
TE 298.2 K
D1 2.00000000 sec
MCREST 0.00000000 sec
MCWRK 0.01500000 sec

======== CHANNEL f1 ========
NUC1 1H
P1 10.70 usec
PL1 -0.50 dB
SFO1 300.1318008 MHz

F2 - Processing parameters
SI 32768
SF 300.1300129 MHz
WDW no
SSB 0
LB 0.00 Hz
GB 0
PC 1.00

1D NMR plot parameters
CX 22.00 cm
CY 6.00 cm
F1P 9.000 ppm
F1 2701.17 Hz
F2P -0.500 ppm
F2 -150.07 Hz
PPMCM 0.43182 ppm/cm
HZCM 129.60159 Hz/cm

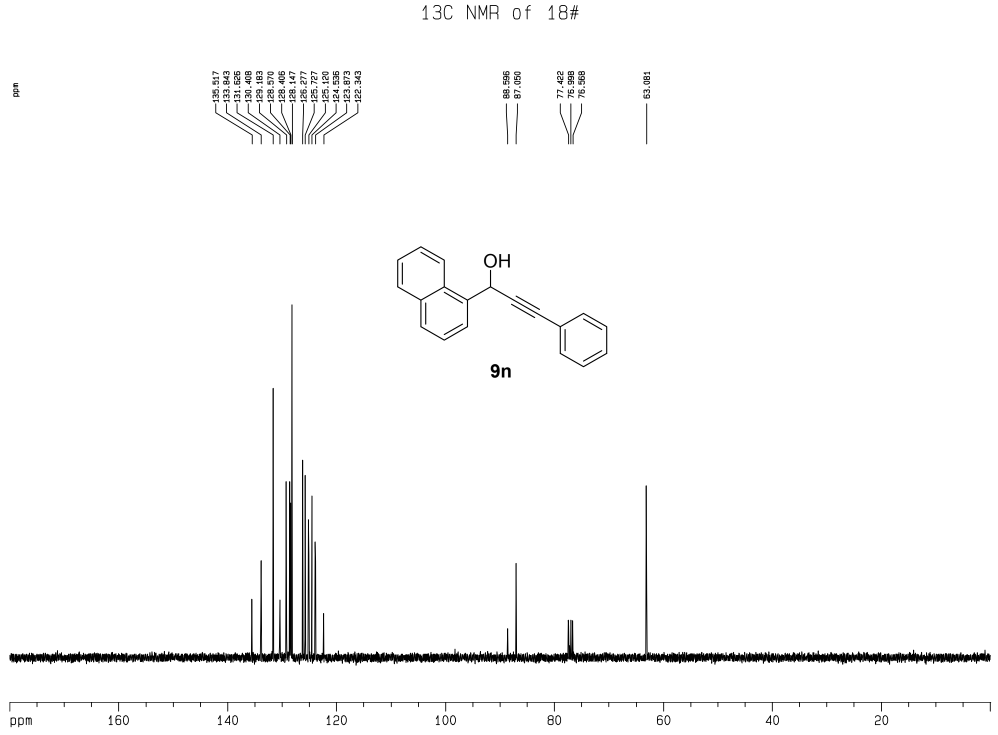

Current Data Parameters
NAME lizhiyuan-13C
EXPNO 12
PROCNO 1

F2 - Acquisition Parameters
Date_ 20090227
Time 21.09
INSTRUM dpx300
PROBHD 5 mm PABBO BB-
PULPROG zgdc30
TD 65536
SOLVENT CDCl3
NS 2048
DS 8
SWH 22675.736 Hz
FIDRES 0.346004 Hz
AQ 1.4451188 sec
RG 8192
DW 22.050 usec
DE 6.00 usec
TE 295.2 K
D1 1.50000000 sec
d11 0.03000000 sec
MCREST 0.00000000 sec
MCWRK 0.01500000 sec

======== CHANNEL f1 ========
NUC1 13C
P1 12.00 usec
PL1 0.00 dB
SFO1 75.4752958 MHz

======== CHANNEL f2 ========
CPDPRG2 waltz16
NUC2 1H
PCPD2 80.00 usec
PL2 -0.50 dB
PL12 17.70 dB
SFO2 300.1315007 MHz

F2 - Processing parameters
SI 32768
SF 75.4677700 MHz
WDW EM
SSB 0
LB 0.20 Hz
GB 0
PC 0.50

1D NMR plot parameters
CX 22.00 cm
CY 8.00 cm
F1P 180.000 ppm
F1 13584.20 Hz
F2P 0.000 ppm
F2 0.00 Hz
PPMCM 8.18182 ppm/cm
HZCM 617.46362 Hz/cm

1H NMR of 19

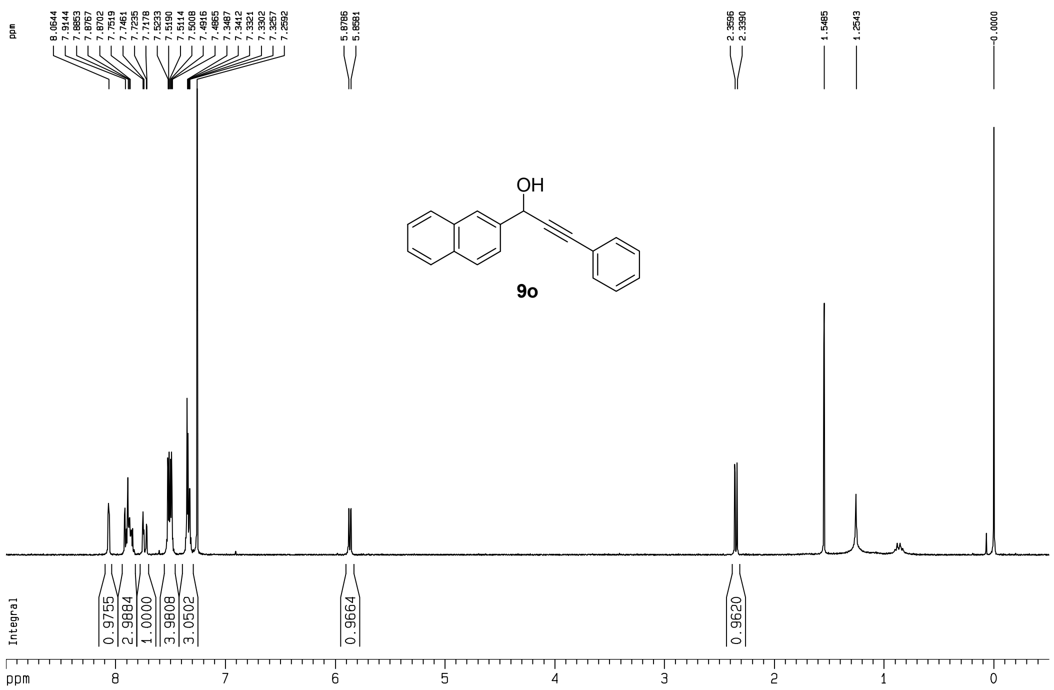

Current Data Parameters	
NAME	lizhiyuan-H
EXPNO	11
PROCNO	1
**F2 - Acquisition Parameters**	
Date_	20090226
Time	10.49
INSTRUM	dpx300
PROBHD	5 mm PABBO BB-
PULPROG	zg30
TD	65536
SOLVENT	CDCl3
NS	12
DS	0
SWH	5995.204 Hz
FIDRES	0.091480 Hz
AQ	5.4657526 sec
RG	258
DW	83.400 usec
DE	6.00 usec
TE	295.2 K
D1	2.00000000 sec
MCREST	0.00000000 sec
MCWRK	0.01500000 sec
**======== CHANNEL f1 ========**	
NUC1	1H
P1	10.70 usec
PL1	-0.50 dB
SFO1	300.1318008 MHz
**F2 - Processing parameters**	
SI	32768
SF	300.1300065 MHz
WDW	EM
SSB	0
LB	0.10 Hz
GB	0
PC	1.00
**1D NMR plot parameters**	
CX	22.00 cm
CY	14.00 cm
F1P	9.000 ppm
F1	2701.17 Hz
F2P	-0.500 ppm
F2	-150.07 Hz
PPMCM	0.43182 ppm/cm
HZCM	129.60159 Hz/cm

13C NMR of 19#

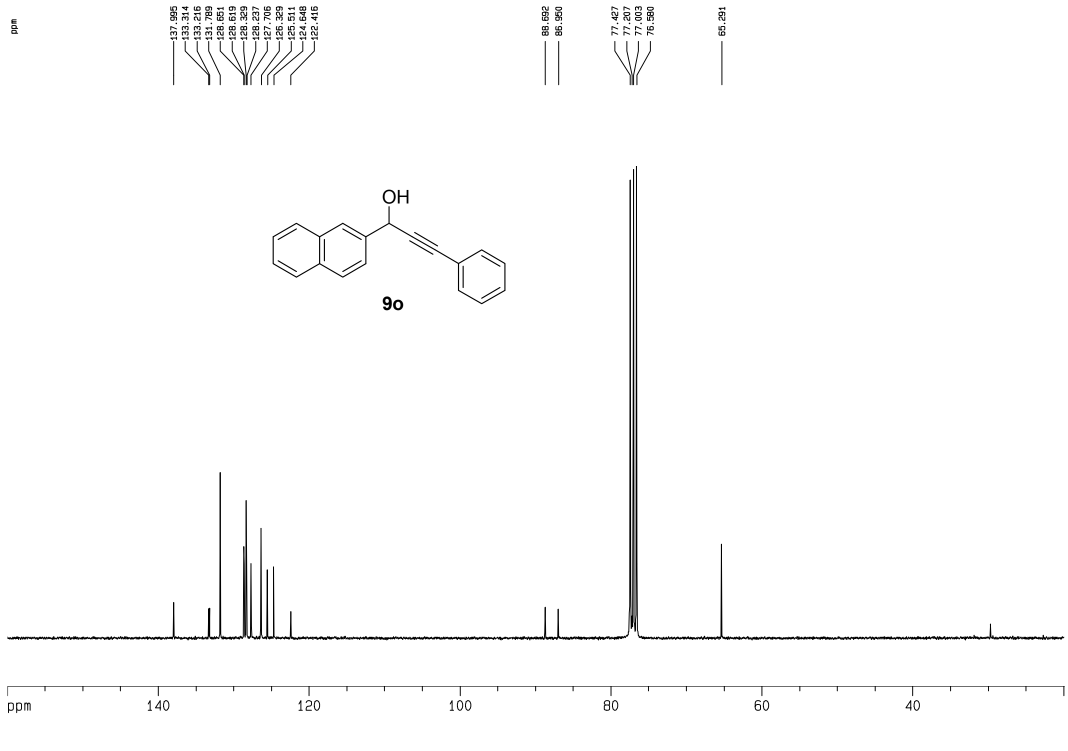

Current Data Parameters	
NAME	lizhiyuan-13C
EXPNO	10
PROCNO	1
**F2 - Acquisition Parameters**	
Date_	20090227
Time	2.27
INSTRUM	dpx300
PROBHD	5 mm PABBO BB-
PULPROG	zgdc30
TD	65536
SOLVENT	CDCl3
NS	8192
DS	8
SWH	22675.736 Hz
FIDRES	0.346004 Hz
AQ	1.4451188 sec
RG	9216
DW	22.050 usec
DE	6.00 usec
TE	299.2 K
D1	1.50000000 sec
d11	0.03000000 sec
MCREST	0.00000000 sec
MCWRK	0.01500000 sec
**======== CHANNEL f1 ========**	
NUC1	13C
P1	12.00 usec
PL1	0.00 dB
SFO1	75.4752958 MHz
**======== CHANNEL f2 ========**	
CPDPRG2	waltz16
NUC2	1H
PCPD2	80.00 usec
PL2	-0.50 dB
PL12	17.70 dB
SFO2	300.1315007 MHz
**F2 - Processing parameters**	
SI	32768
SF	75.4677499 MHz
WDW	EM
SSB	0
LB	1.00 Hz
GB	0
PC	0.50
**1D NMR plot parameters**	
CX	22.00 cm
CY	10.00 cm
F1P	160.000 ppm
F1	12074.84 Hz
F2P	20.000 ppm
F2	1509.35 Hz
PPMCM	6.36364 ppm/cm
HZCM	480.24930 Hz/cm

1H NMR of 21#

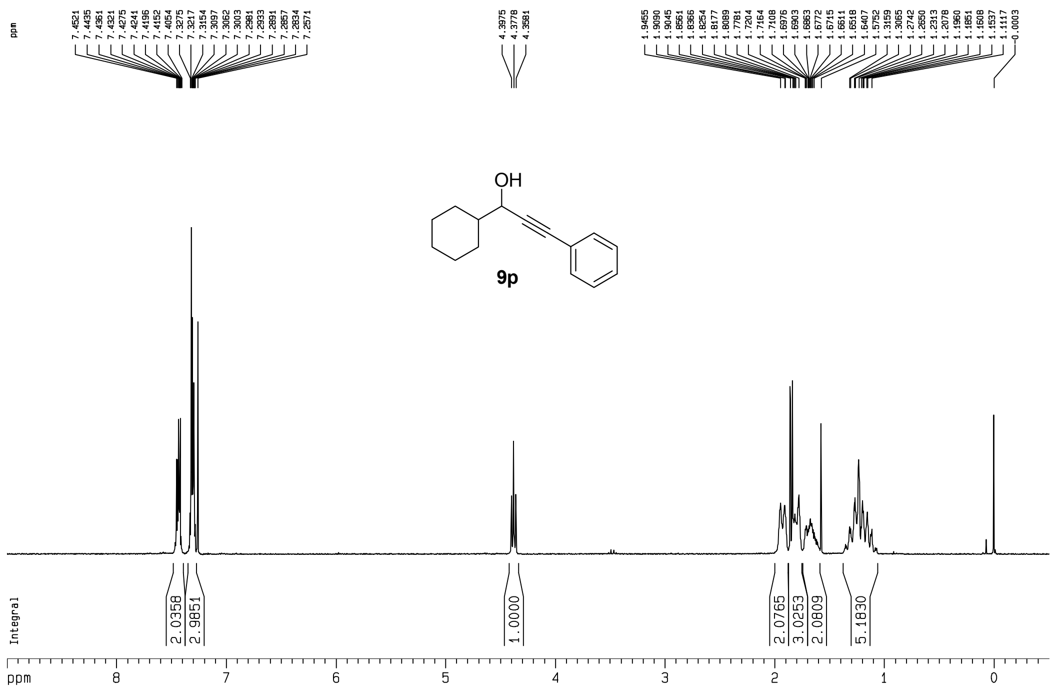

Current Data Parameters
NAME lizhiyuan-H
EXPNO 45
PROCNO 1

F2 - Acquisition Parameters
Date_ 20090415
Time 9.59
INSTRUM dpx300
PROBHD 5 mm PABBO BB-
PULPROG zg30
TD 65536
SOLVENT CDCl3
NS 4
DS 0
SWH 5995.204 Hz
FIDRES 0.091480 Hz
AQ 5.4657526 sec
RG 258
DW 83.400 usec
DE 6.00 usec
TE 299.2 K
D1 2.00000000 sec
MCREST 0.00000000 sec
MCWRK 0.01500000 sec

======== CHANNEL f1 ========
NUC1 1H
P1 10.70 usec
PL1 -0.50 dB
SFO1 300.1318008 MHz

F2 - Processing parameters
SI 32768
SF 300.1300072 MHz
WDW EM
SSB 0
LB 0.10 Hz
GB 0
PC 1.00

1D NMR plot parameters
CX 22.00 cm
CY 7.00 cm
F1P 9.000 ppm
F1 2701.17 Hz
F2P -0.500 ppm
F2 -150.07 Hz
PPMCM 0.43182 ppm/cm
HZCM 129.60159 Hz/cm

13C NMR of 21#

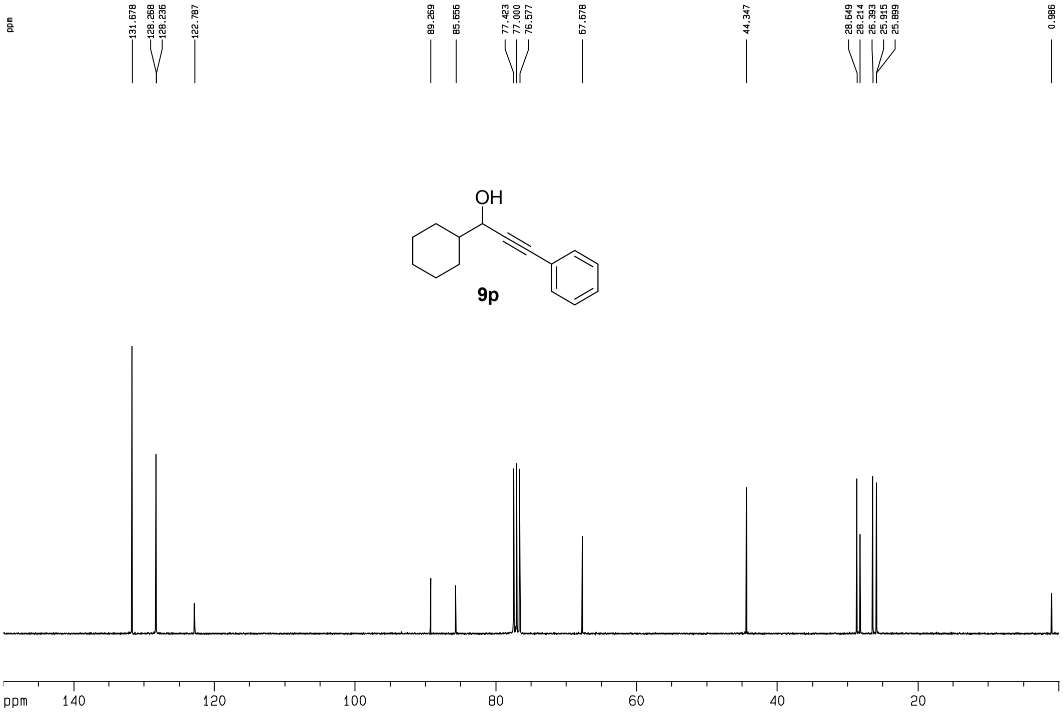

Current Data Parameters
NAME lizhiyuan-13C
EXPNO 29
PROCNO 1

F2 - Acquisition Parameters
Date_ 20090415
Time 22.13
INSTRUM dpx300
PROBHD 5 mm PABBO BB-
PULPROG zgdc30
TD 65536
SOLVENT CDCl3
NS 4222
DS 8
SWH 22675.736 Hz
FIDRES 0.346004 Hz
AQ 1.4451188 sec
RG 6144
DW 22.050 usec
DE 6.00 usec
TE 300.2 K
D1 1.50000000 sec
d11 0.03000000 sec
MCREST 0.00000000 sec
MCWRK 0.01500000 sec

======== CHANNEL f1 ========
NUC1 13C
P1 12.00 usec
PL1 0.00 dB
SFO1 75.4752958 MHz

======== CHANNEL f2 ========
CPDPRG2 waltz16
NUC2 1H
PCPD2 80.00 usec
PL2 -0.50 dB
PL12 17.70 dB
SFO2 300.1315007 MHz

F2 - Processing parameters
SI 32768
SF 75.4677506 MHz
WDW EM
SSB 0
LB 0.50 Hz
GB 0
PC 0.50

1D NMR plot parameters
CX 22.00 cm
CY 6.00 cm
F1P 150.000 ppm
F1 11320.16 Hz
F2P -0.000 ppm
F2 -0.00 Hz
PPMCM 6.81818 ppm/cm
HZCM 514.55286 Hz/cm

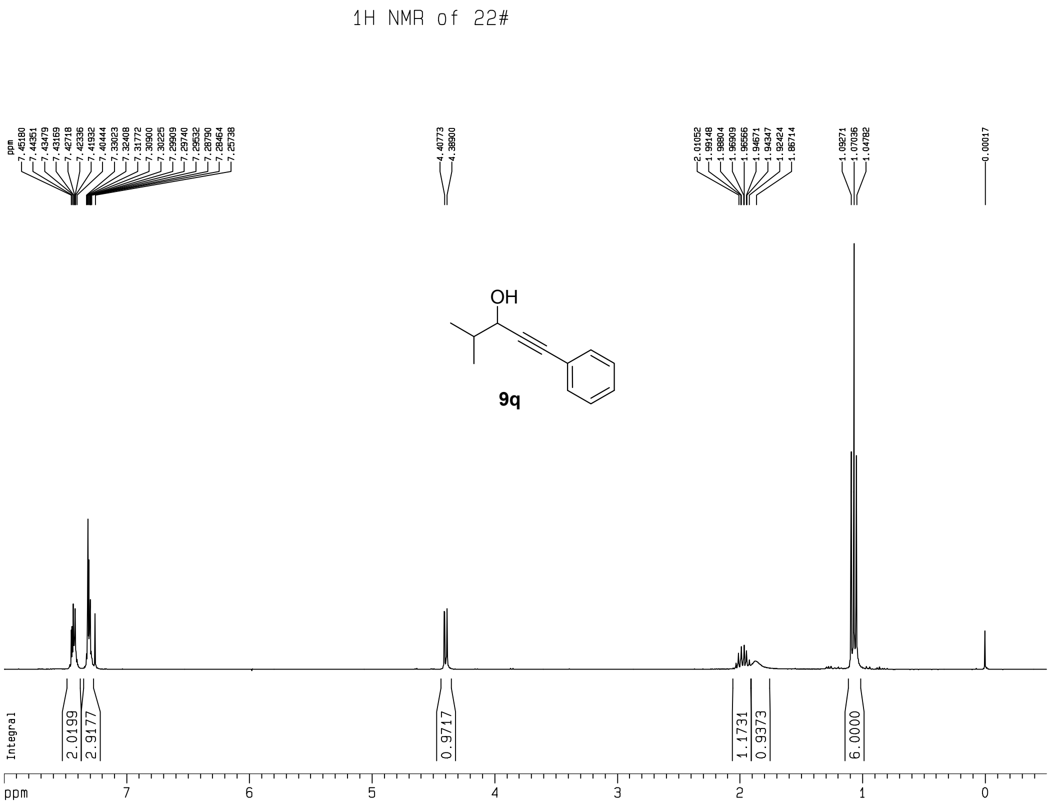


```
Current Data Parameters
NAME        lizhiyuan-H
EXPNO                40
PROCNO                1

F2 - Acquisition Parameters
Date_          20090409
Time              11.34
INSTRUM          dpx300
PROBHD   5 mm PABBO BB-
PULPROG            zg30
TD                65536
SOLVENT           CDCl3
NS                    4
DS                    0
SWH            5995.204 Hz
FIDRES         0.091480 Hz
AQ            5.4657526 sec
RG                  128
DW               83.400 usec
DE                 6.00 usec
TE                297.2 K
D1           2.00000000 sec
MCREST       0.00000000 sec
MCWRK        0.01500000 sec

======== CHANNEL f1 ========
NUC1                 1H
P1                10.00 usec
PL1               -2.50 dB
SFO1        300.1318008 MHz

F2 - Processing parameters
SI                32768
SF          300.1300070 MHz
WDW                  EM
SSB                   0
LB                 0.10 Hz
GB                    0
PC                 1.00

1D NMR plot parameters
CX                22.00 cm
CY                 9.00 cm
F1P               8.000 ppm
F1              2401.04 Hz
F2P              -0.500 ppm
F2              -150.06 Hz
PPMCM           0.38636 ppm/cm
HZCM          115.95932 Hz/cm
```

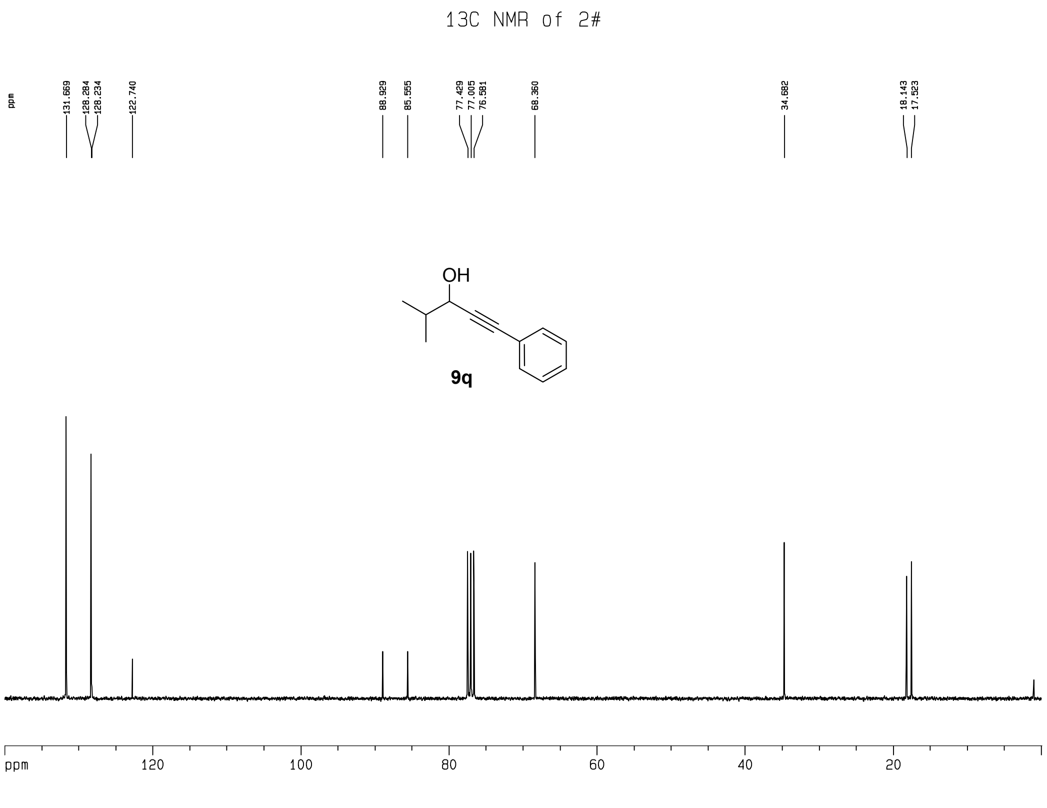


```
Current Data Parameters
NAME      lizhiyuan-13C
EXPNO                25
PROCNO                1

F2 - Acquisition Parameters
Date_          20090411
Time              12.28
INSTRUM          dpx300
PROBHD   5 mm PABBO BB-
PULPROG         zgdc30
TD                65536
SOLVENT           CDCl3
NS                  298
DS                    8
SWH           22675.736 Hz
FIDRES         0.346004 Hz
AQ            1.4451188 sec
RG                 2048
DW               22.050 usec
DE                 6.00 usec
TE                299.2 K
D1           1.50000000 sec
d11          0.03000000 sec
MCREST       0.00000000 sec
MCWRK        0.01500000 sec

======== CHANNEL f1 ========
NUC1                13C
P1                12.00 usec
PL1                0.90 dB
SFO1         75.4752958 MHz

======== CHANNEL f2 ========
CPDPRG2         waltz16
NUC2                 1H
PCPD2             80.00 usec
PL2               -2.50 dB
PL12              18.00 dB
SFO2        300.1315007 MHz

F2 - Processing parameters
SI                32768
SF           75.4677520 MHz
WDW                  EM
SSB                   0
LB                 1.00 Hz
GB                    0
PC                 0.50

1D NMR plot parameters
CX                22.00 cm
CY                 6.00 cm
F1P             140.000 ppm
F1             10565.49 Hz
F2P               0.000 ppm
F2                 0.00 Hz
PPMCM           6.36364 ppm/cm
HZCM          480.24930 Hz/cm
```

Figure S2. HPLC Spectra of the Propargylic Alcohols Products.

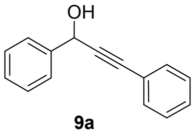

9a

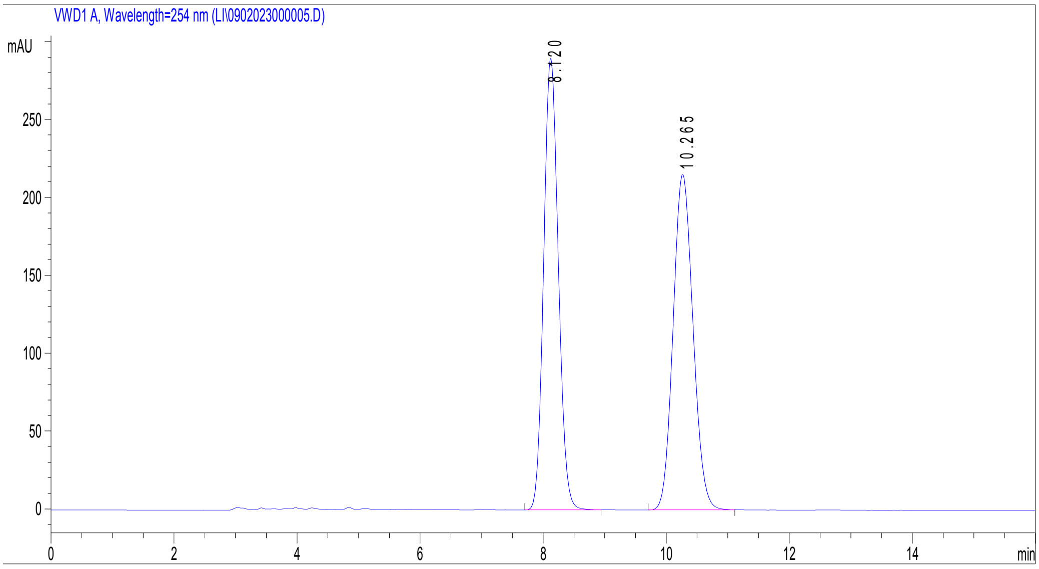

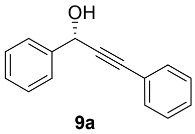

9a

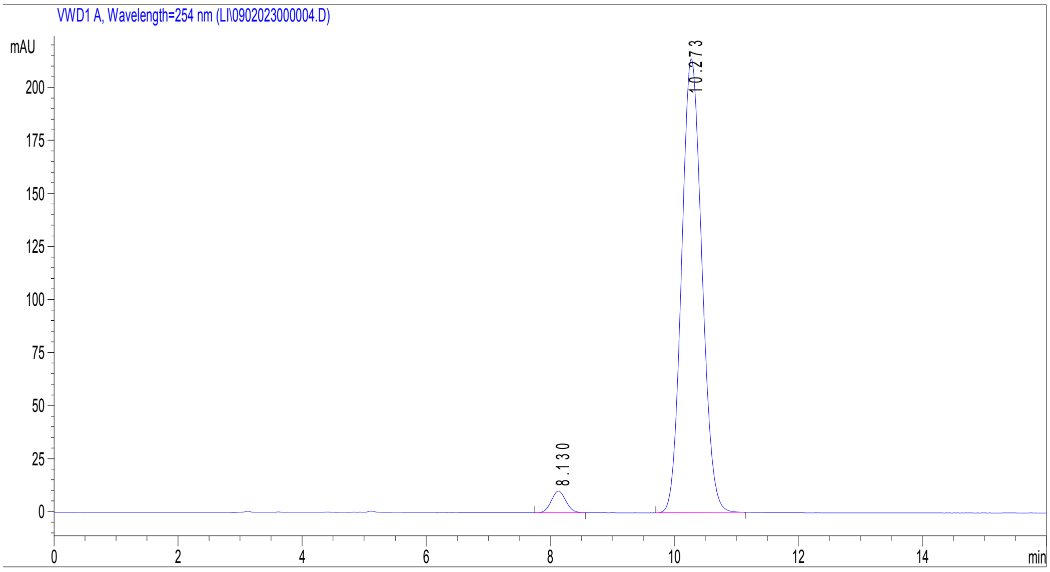

Peak RetTime Type Width Area Height Area
[min] [min] mAU *s [mAU] %
------|-----------|-----|-----------|---------------|---------------|-------------|
 1 8.130 BB 0.2539 167.02376 10.18796 3.3201
 2 10.273 BB 0.3569 4863.61865 213.96878 96.6799
Totals: 5030.64241 224.15674

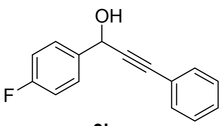

9b

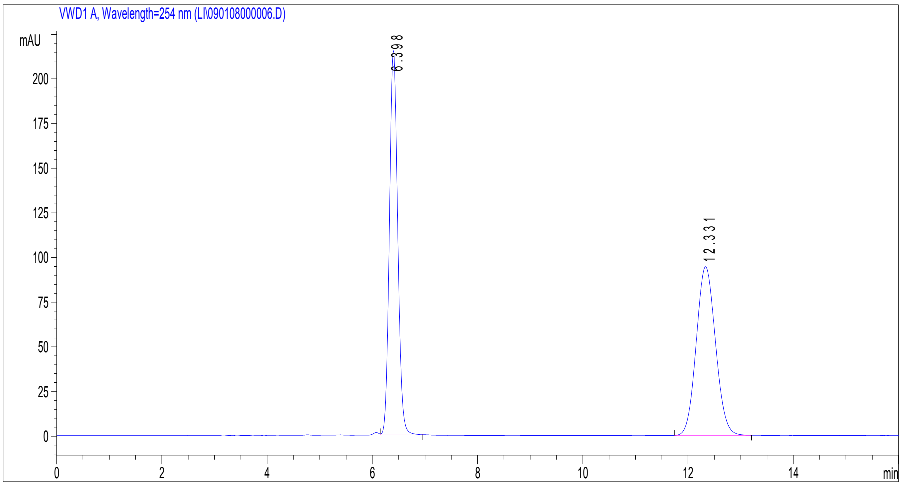

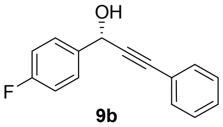

9b

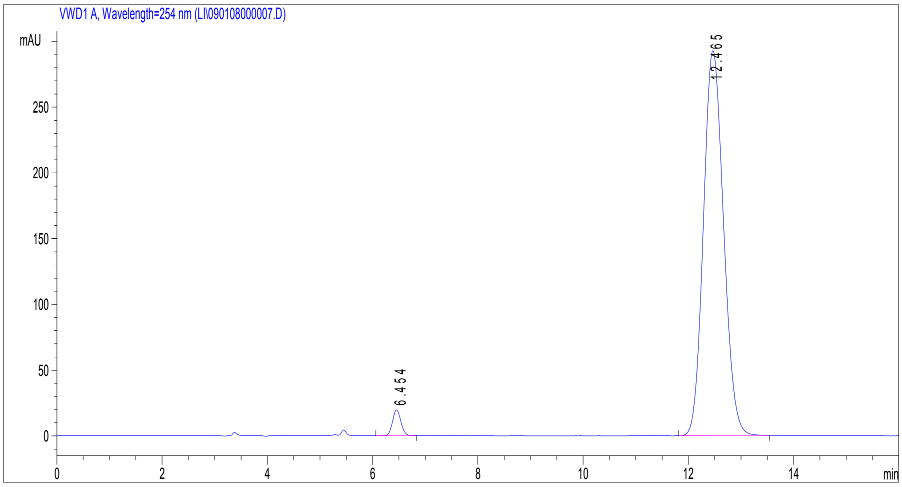

Peak #	RetTime [min]	Type	Width [min]	Area mAU *s	Height [mAU]	Area %
1	6.454	BB	0.1741	219.45233	19.63624	2.7447
2	12.465	BB	0.4161	7775.95850	292.79892	97.2553
Totals:				7995.41083	312.43516	

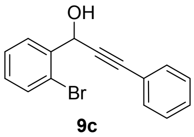

9c

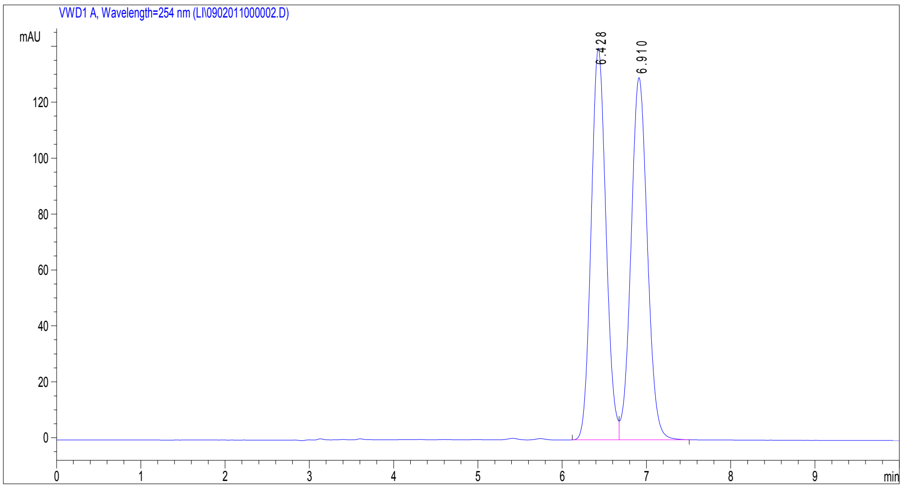

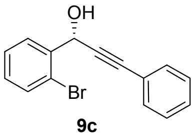

9c

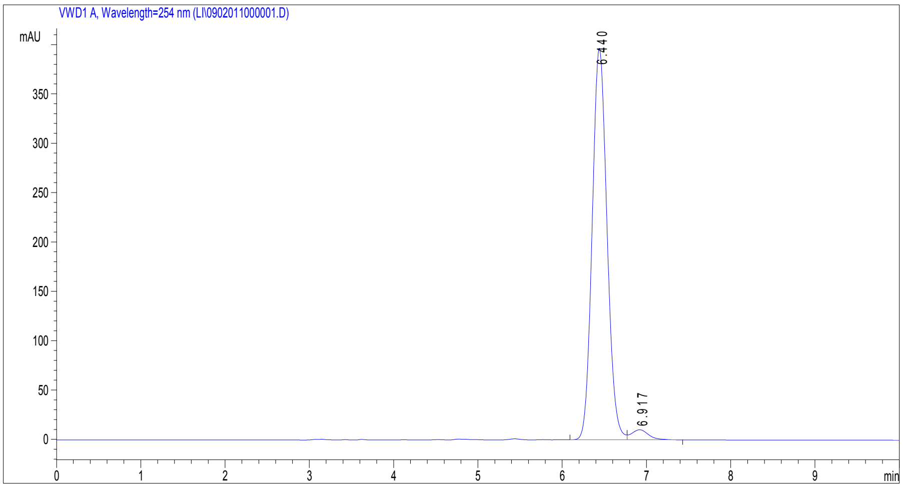

Peak #	RetTime [min]	Type	Width [min]	Area mAU *s	Height [mAU]	Area %
1	6.440	VV	0.1908	4856.67627	397.02255	97.0249
2	6.917	BB	0.2160	148.92174	10.34672	2.9751

Totals: 5005.59801 407.36927

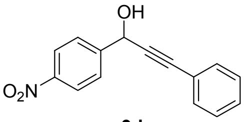

9d

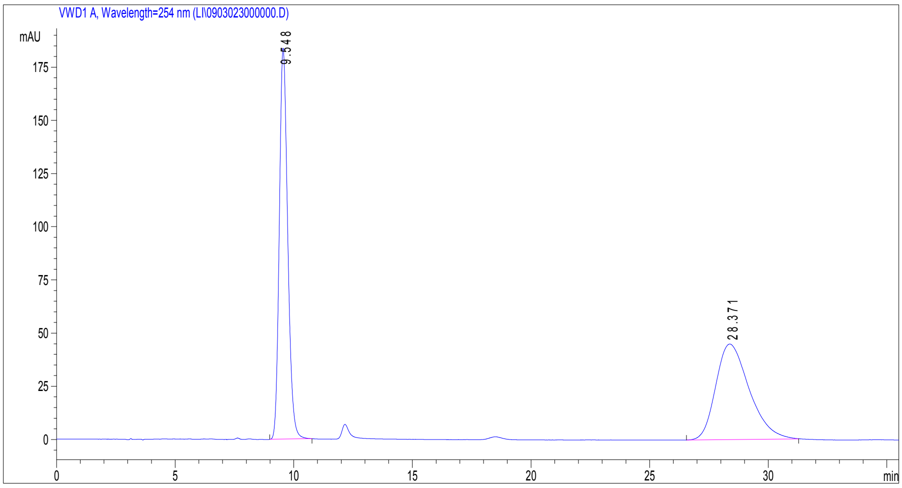

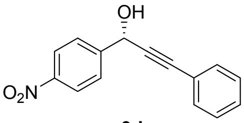

9d

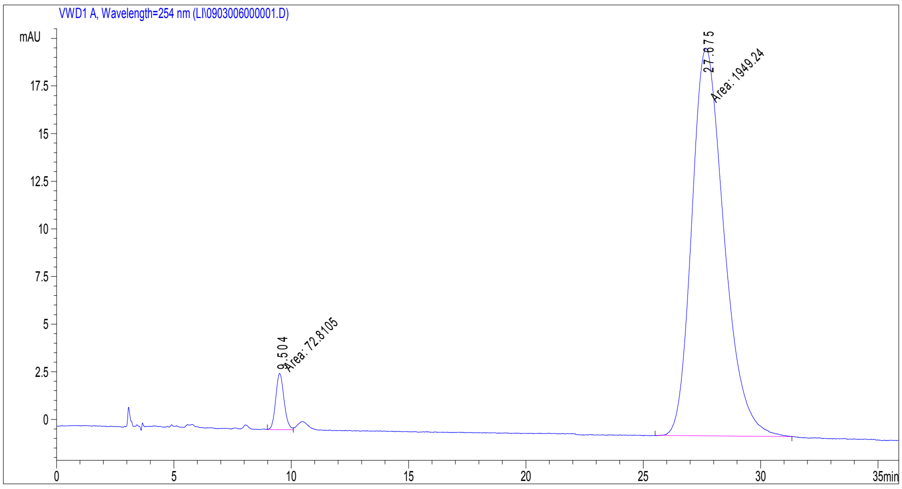

Peak #	RetTime [min]	Type	Width [min]	Area mAU *s	Height [mAU]	Area %
1	9.504	BV	0.3701	72.11470	2.94545	3.6785
2	27.675	BB	1.1559	1888.30469	20.17732	96.3215
Totals:				1960.41939	23.12277	

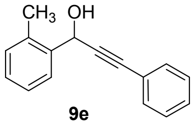

9e

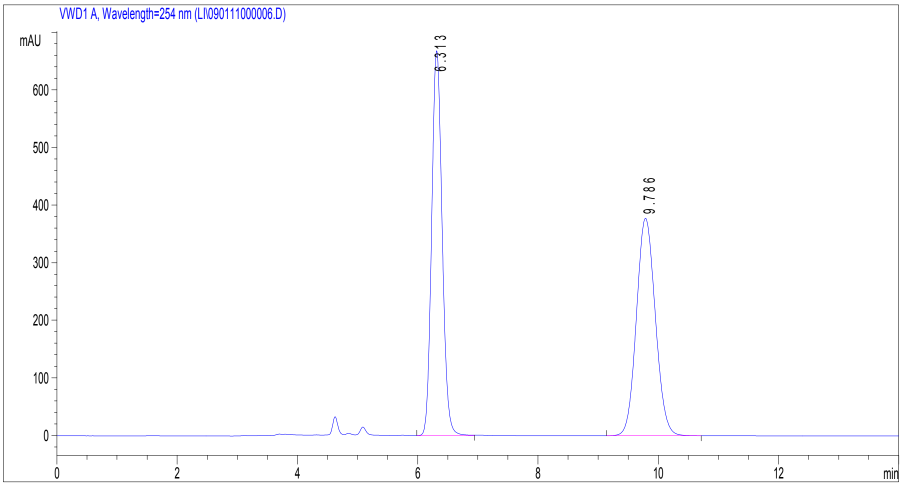

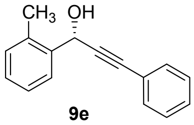

9e

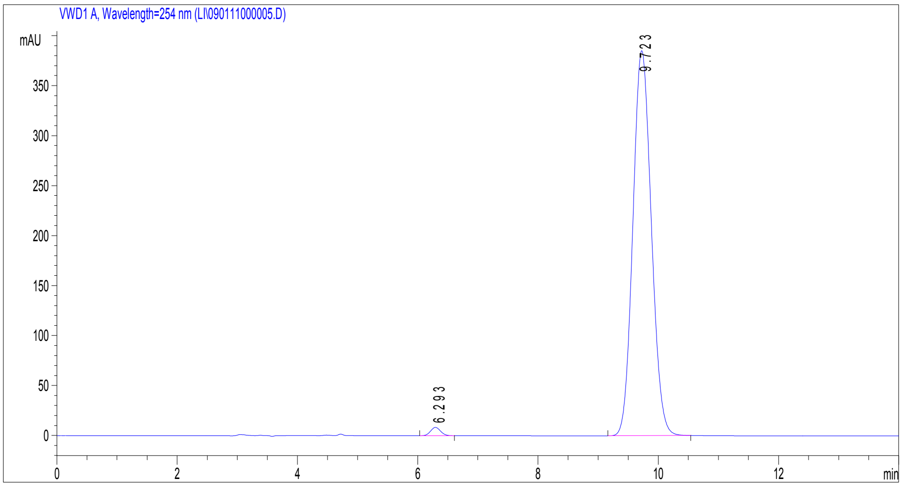

Peak RetTime Type Width Area Height Area
[min] [min] mAU *s [mAU] %
------|-----------|-----|------------|---------------|---------------|-------------|
1 6.293 BB 0.1805 96.56470 8.32010 1.1636
2 9.723 BB 0.3328 8202.46777 385.29794 98.8364
Totals: 8299.03247 393.61805

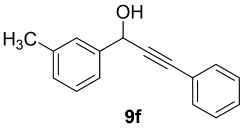

9f

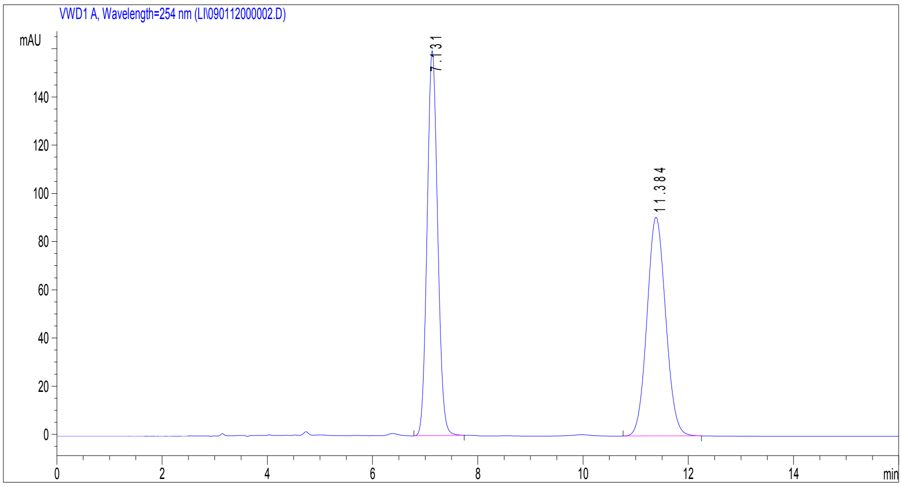

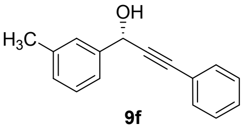

9f

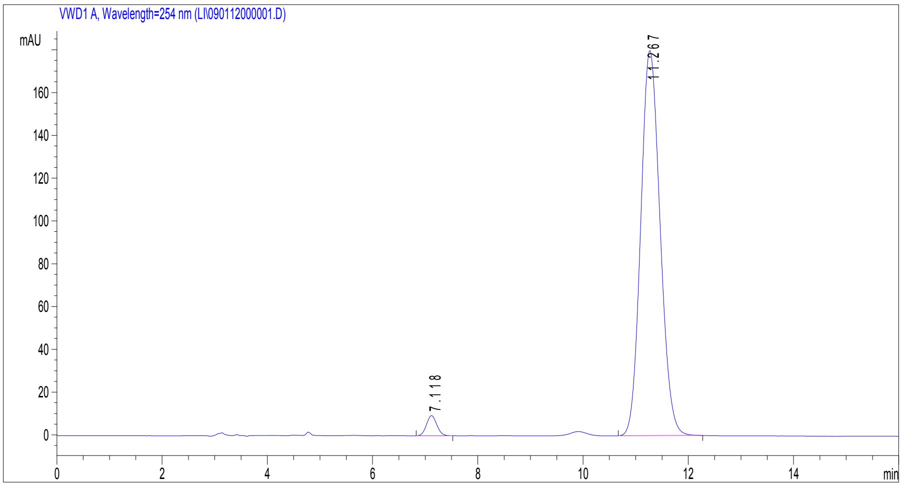

Peak RetTime Type Width Area Height Area
[min] [min] mAU *s [mAU] %
------|-----------|-----|------------|---------------|---------------|-------------|
1 7.118 BB 0.2128 129.48990 9.41662 2.7356
2 11.267 BB 0.3988 4604.02734 180.01799 97.2644
Totals: 4733.51724 189.43461

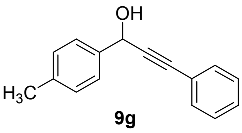

9g

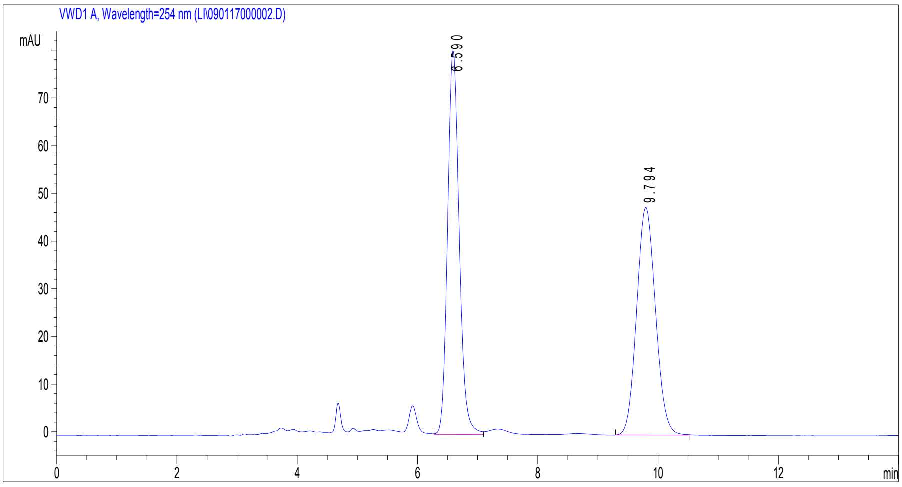

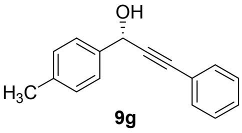

9g

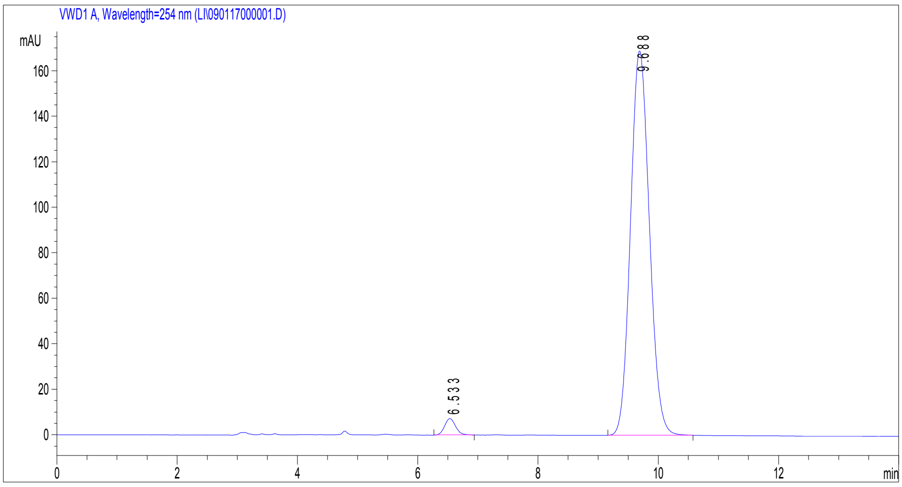

Peak RetTime Type Width Area Height Area
[min] [min] mAU *s [mAU] %
------|-----------|-----|------------|---------------|---------------|-------------|
1 6.533 BB 0.1980 92.27942 7.24765 2.4403
2 9.688 BB 0.3421 3689.23779 168.98087 97.5597
Totals: 3781.51721 176.22851

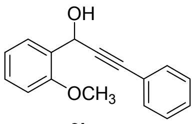

9h

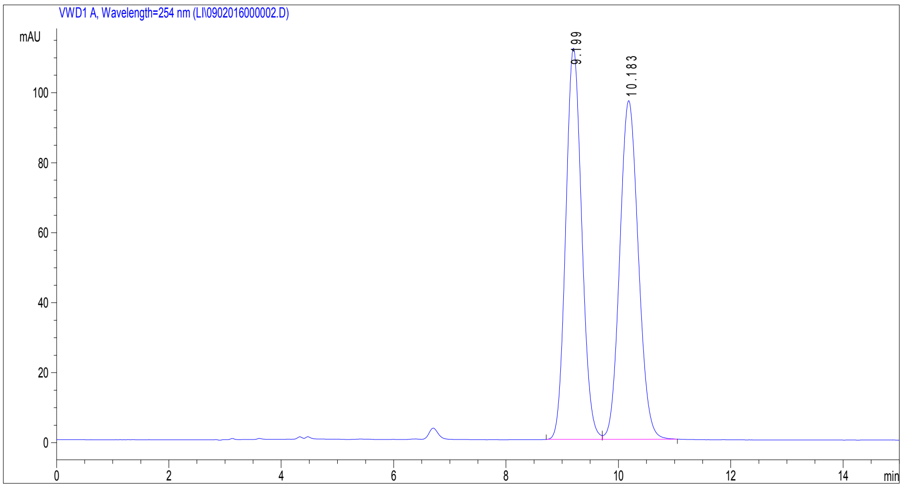

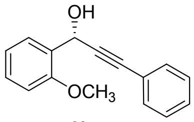

9h

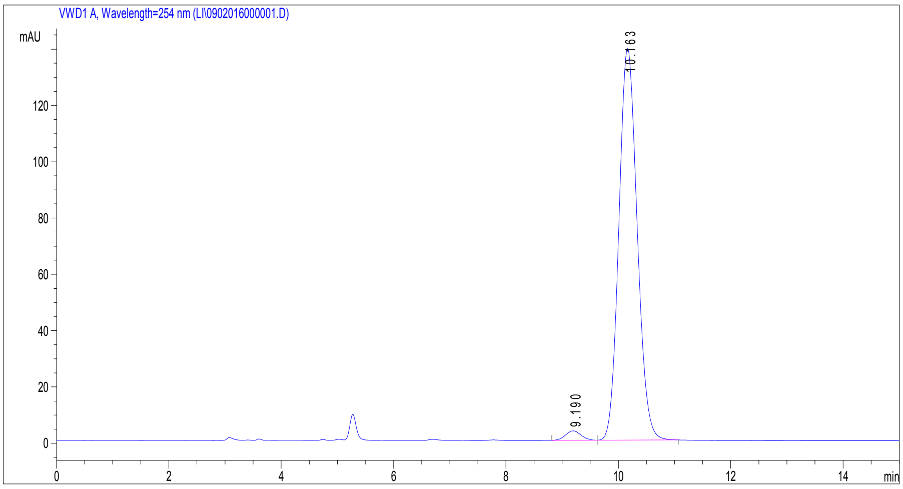

Peak #	RetTime [min]	Type	Width [min]	Area mAU *s	Height [mAU]	Area %
1	9.190	BV	0.3002	65.34696	3.37397	2.0347
2	10.163	VB	0.3526	3146.21997	139.17323	97.9653
Totals:				3211.56693	142.54721	

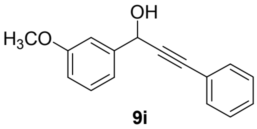

9i

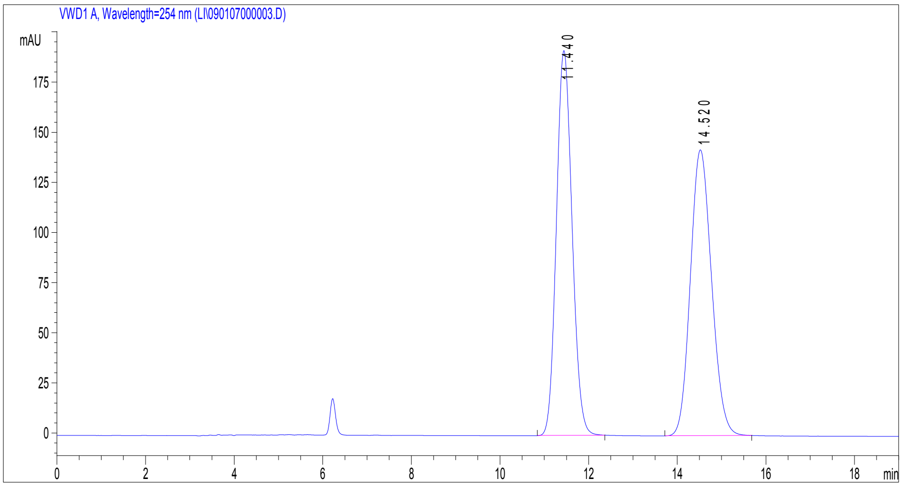

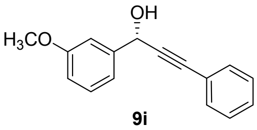

9i

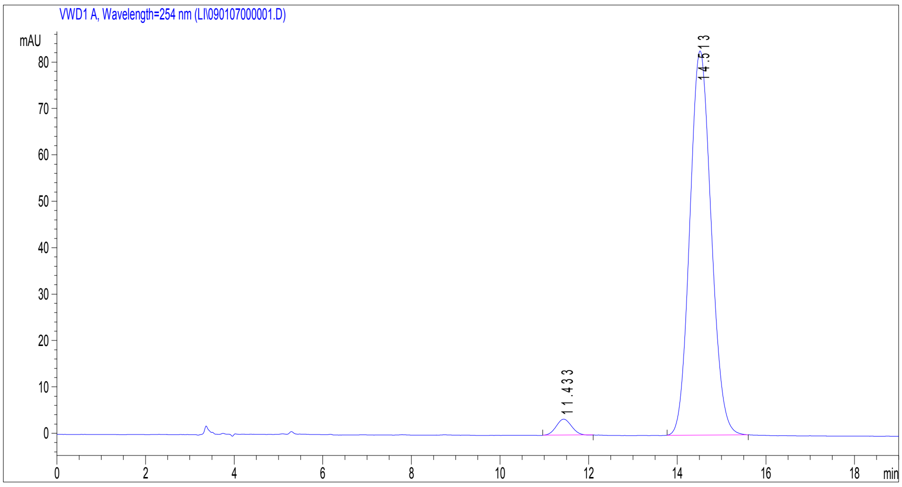

Peak #	RetTime [min]	Type	Width [min]	Area mAU *s	Height [mAU]	Area %
1	11.433	BB	0.3651	84.38023	3.47189	2.9429
2	14.513	BB	0.5229	2782.89478	82.85305	97.0571

Totals: 2867.27500 86.32494

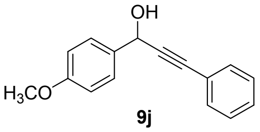

9j

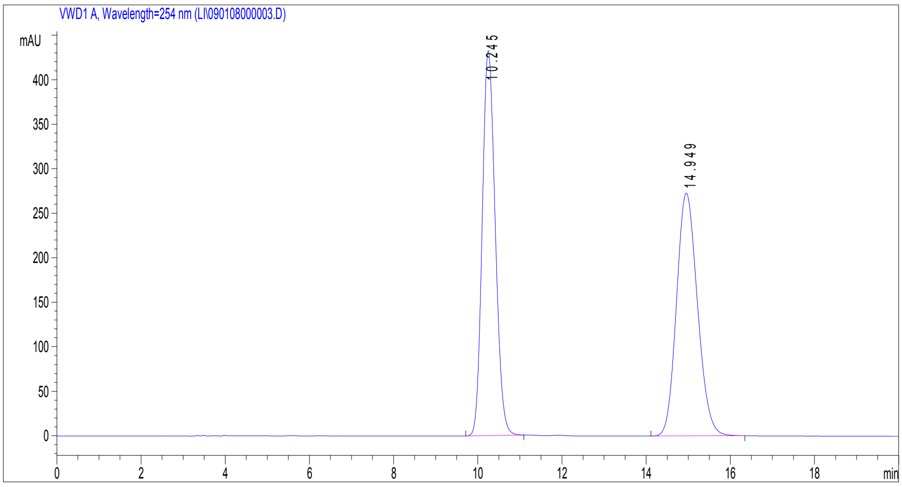

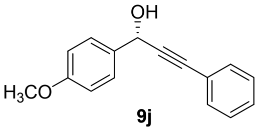

9j

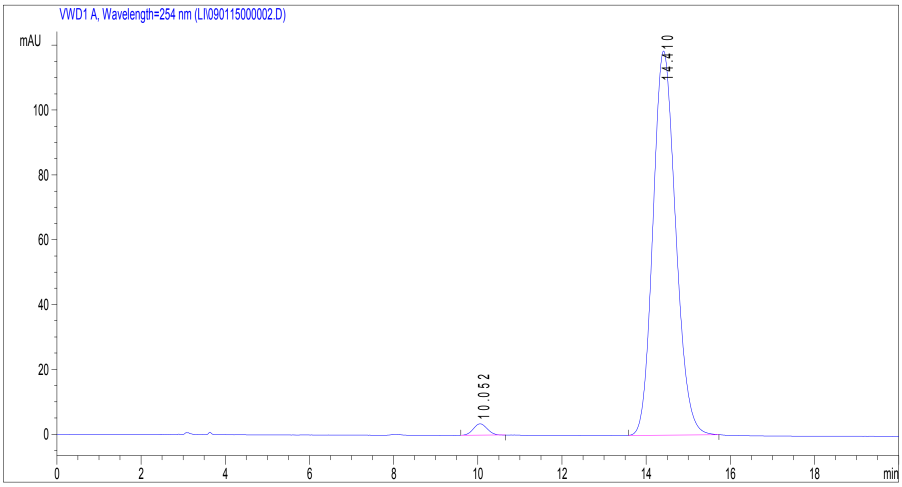

Peak RetTime Type Width Area Height Area
[min] [min] mAU *s [mAU] %
------|-----------|-----|-----------|---------------|---------------|-------------|
1 10.052 BB 0.3574 80.70335 3.50592 1.7836
2 14.410 BB 0.5828 4443.92822 118.57702 98.2164
Totals: 4524.63157 122.08293

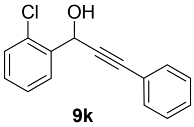

9k

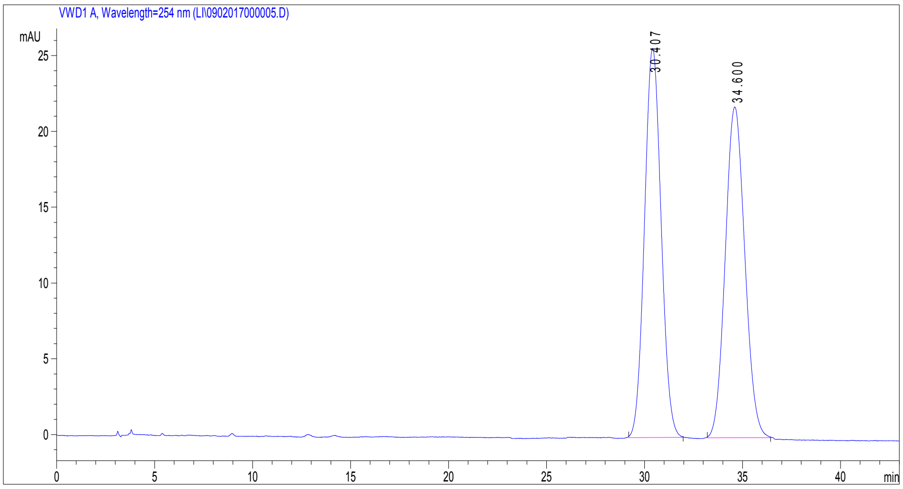

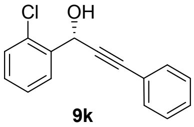

9k

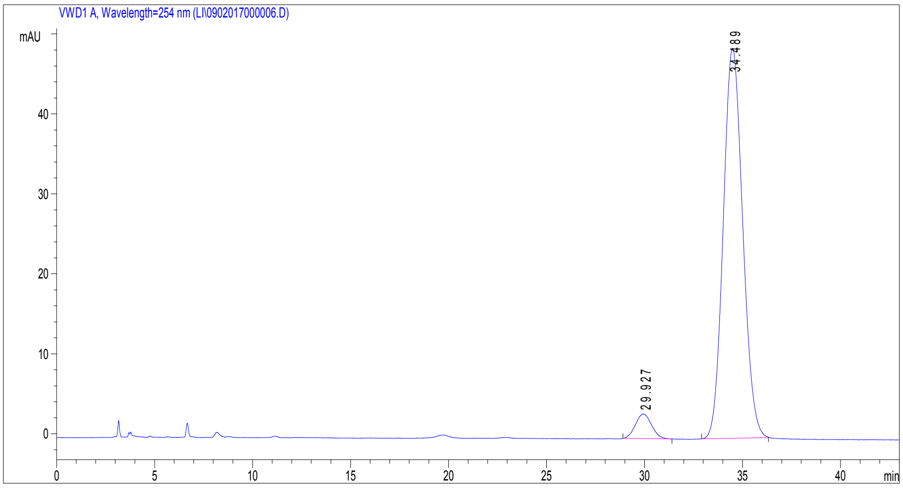

Peak RetTime Type Width Area Height Area
[min] [min] mAU *s [mAU] %
------|-----------|-----|-----------|---------------|---------------|-------------|
1 29.927 BB 0.6856 178.26758 3.09936 5.0314
2 34.489 BB 1.0546 3364.80078 48.81406 94.9686
Totals: 3543.06836 51.91342

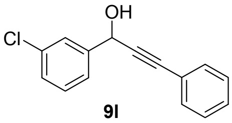

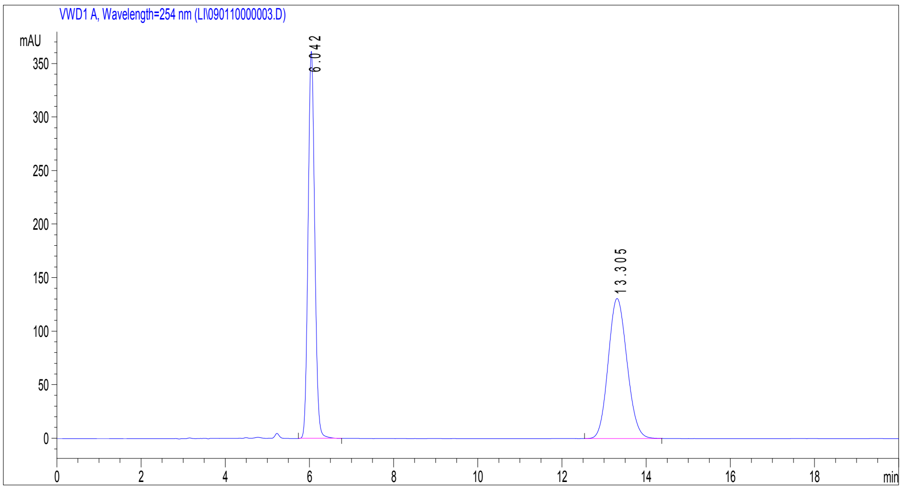

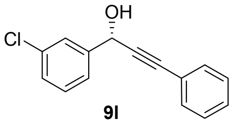

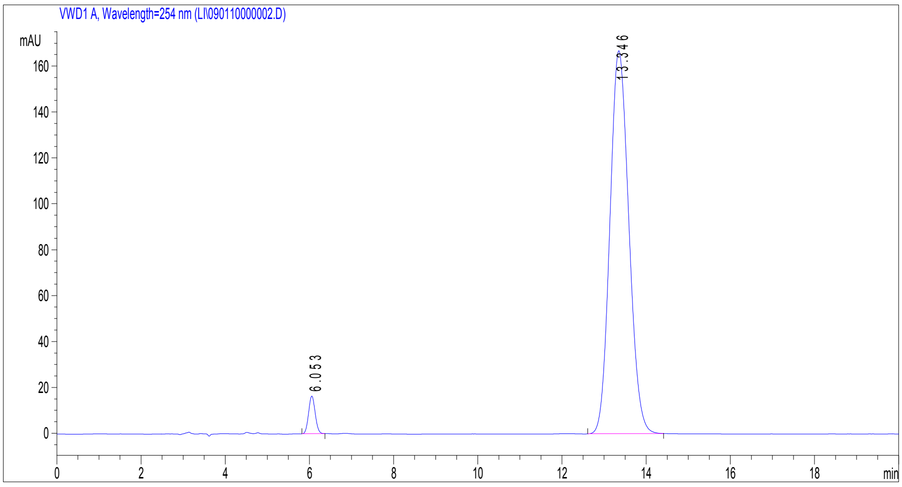

Peak #	RetTime [min]	Type	Width [min]	Area mAU *s	Height [mAU]	Area %
1	6.053	BB	0.1713	180.19814	16.47809	3.3396
2	13.346	BB	0.4898	5215.59277	166.92085	96.6604
Totals:				5395.79091	183.39894	

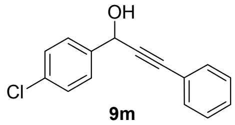

9m

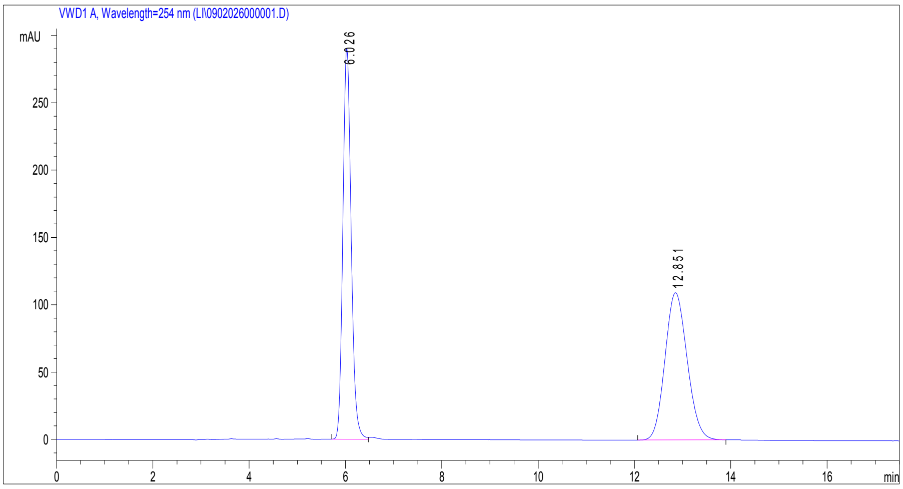

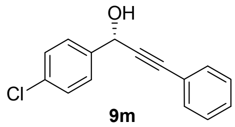

9m

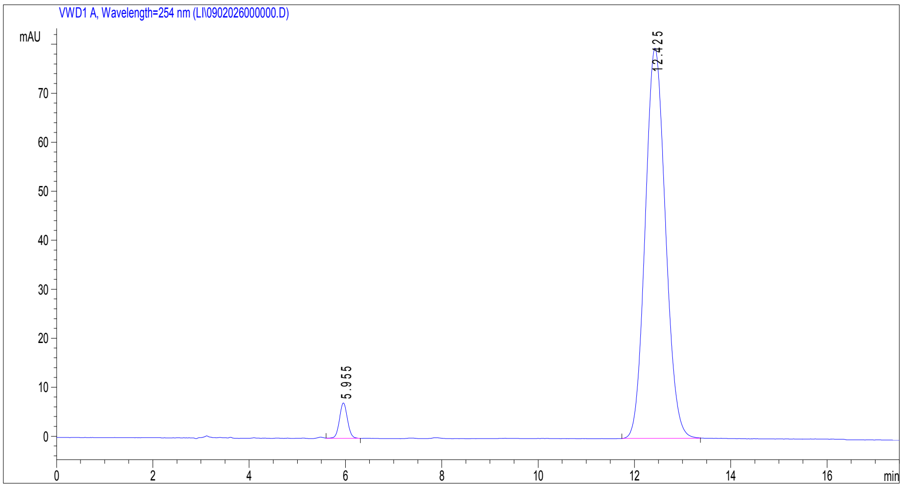

Peak RetTime Type Width Area Height Area
[min] [min] mAU *s [mAU] %
------|-----------|-----|-----------|---------------|---------------|-------------|
1 5.955 VB 0.1813 84.62672 7.24935 3.5058
2 12.425 BB 0.4562 2329.29492 79.66242 96.4942
Totals: 2413.92165 86.91177

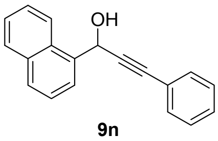

9n

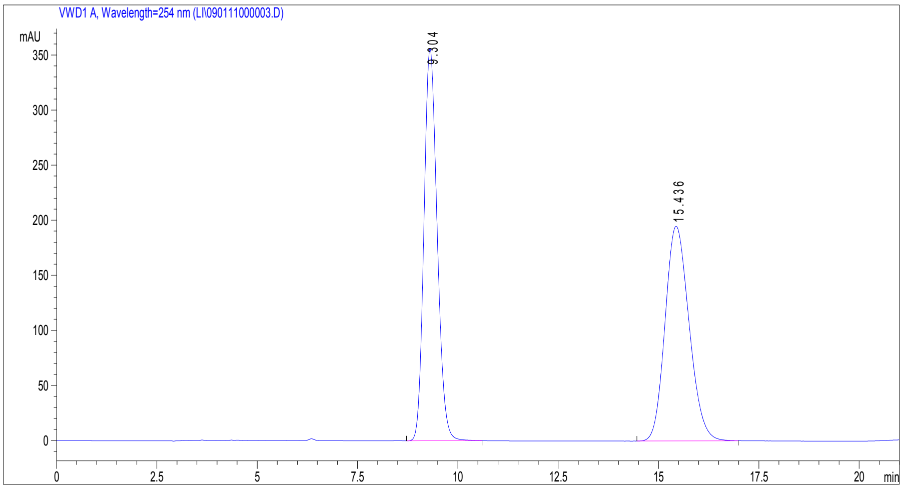

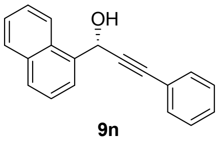

9n

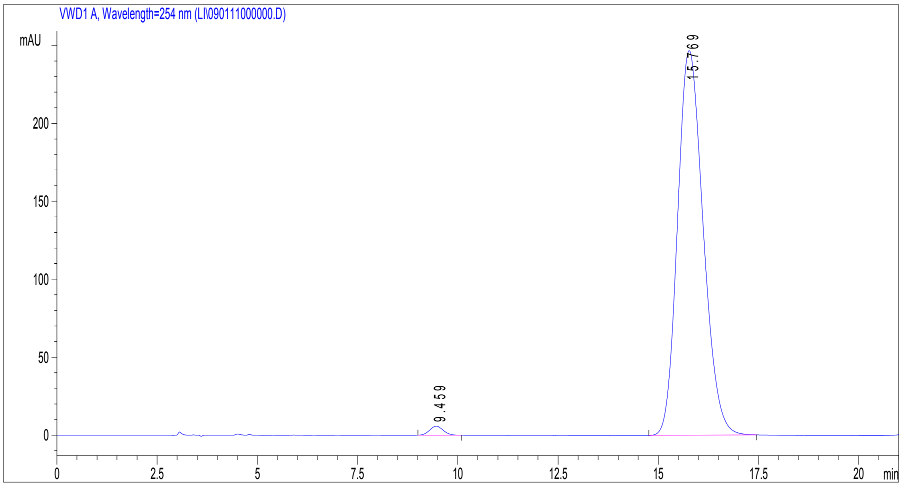

Peak RetTime Type Width Area Height Area
[min] [min] mAU *s [mAU] %

------|-----------|-----|-----------|---------------|---------------|-------------|
1 9.459 BB 0.3679 167.02376 5.82216 1.2526
2 15.769 BB 0.6908 1.09811e4 246.83197 98.7474
Totals: 1.11204e4 252.65413

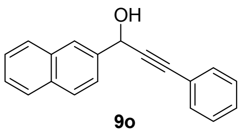

9o

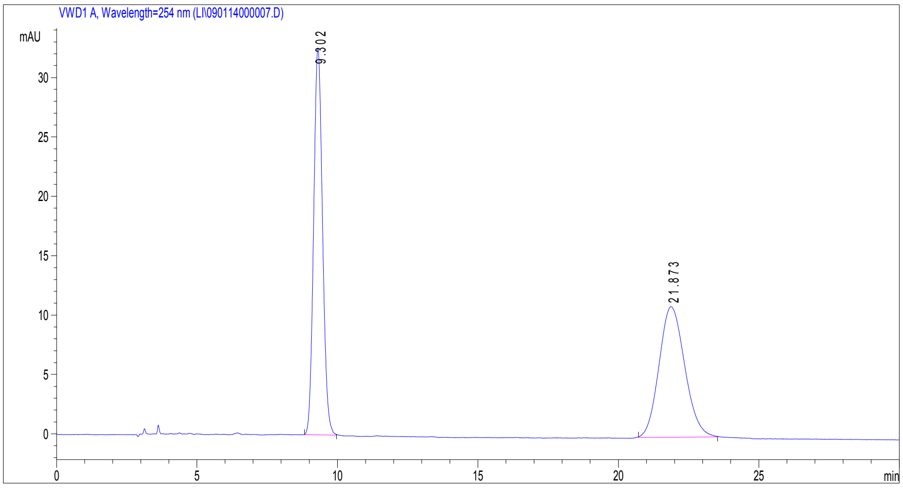

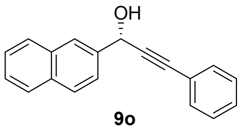

9o

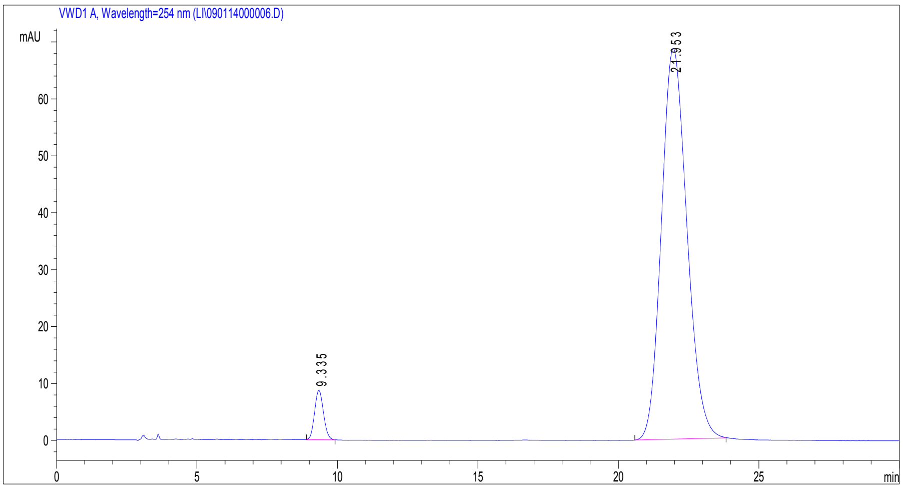

Peak RetTime Type Width Area Height Area

[min] [min] mAU *s [mAU] %

----|-------|---|-------|----------|----------|--------|

1 9.335 BB 0.3430 191.04559 8.66960 4.2188

2 21.953 BB 0.9746 4337.38818 68.63472 95.7812

Totals: 4528.43378 77.30432

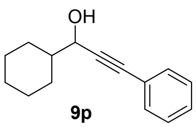

9p

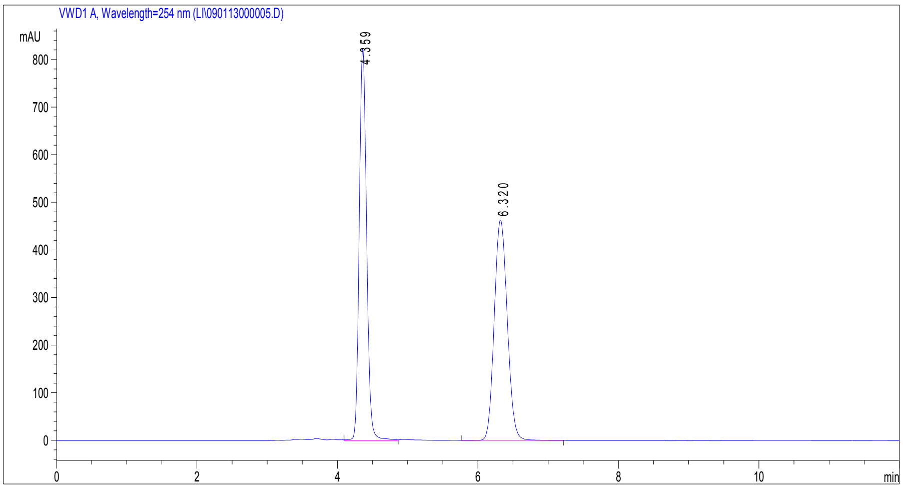

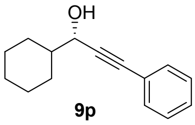

9p

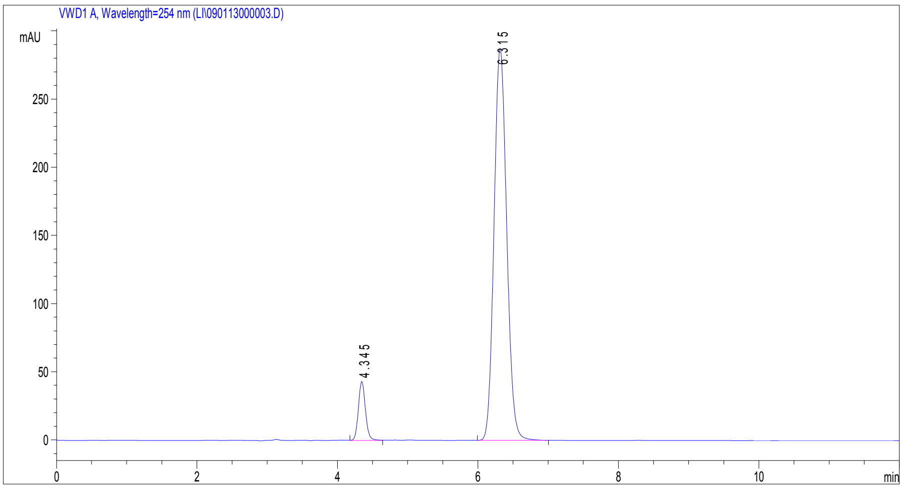

Peak RetTime Type Width Area Height Area

[min] [min] mAU *s [mAU] %

------|-----------|-----|-----------|---------------|----------------|-------------|

1 4.345 BB 0.1086 305.69379 43.29863 7.8295

2 6.315 BB 0.1956 3598.67700 287.41974 92.1705

Totals: 3904.37079 330.71836

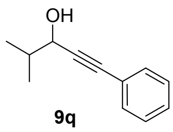

9q

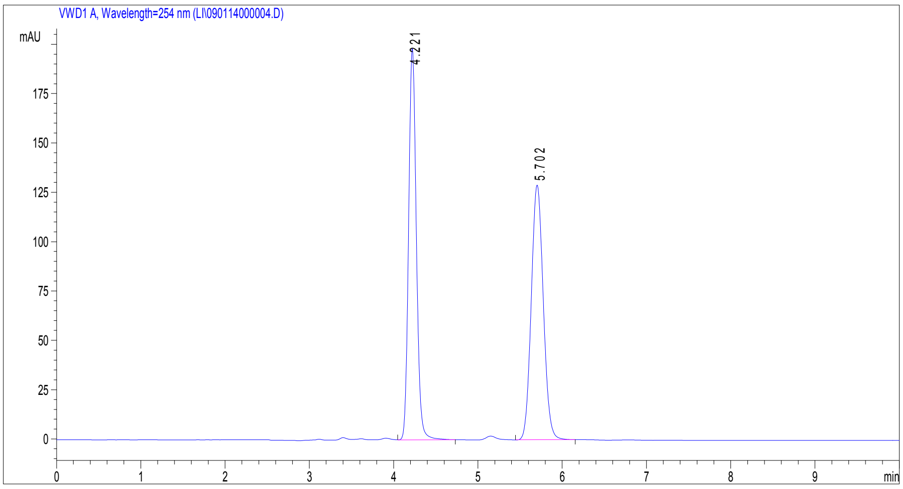

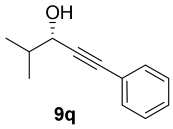

9q

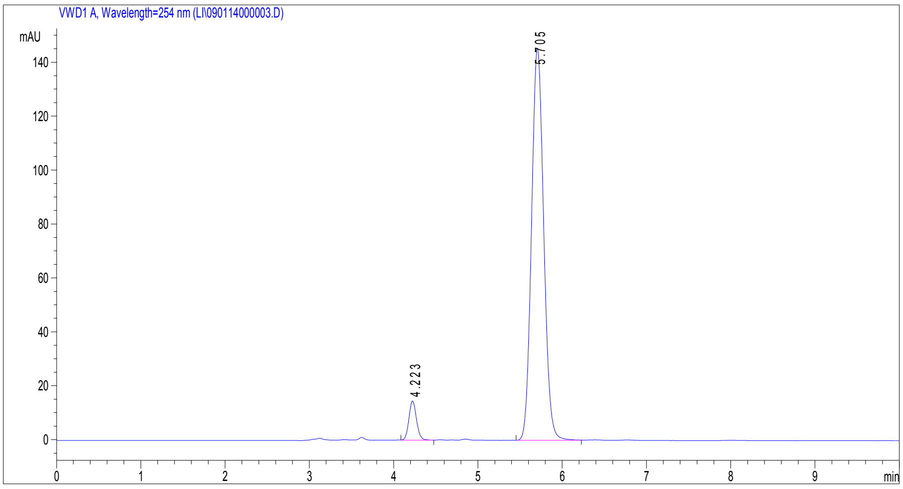

Peak RetTime Type Width Area Height Area
[min] [min] mAU *s [mAU] %
------|-----------|-----|-----------|---------------|---------------|-------------|
1 4.223 BB 0.0975 90.75805 14.55558 5.8361
2 5.705 BB 0.1567 1464.34680 145.50090 94.1639
Totals: 1555.10485 160.05648
